# Transcriptome profiling of granulosa cells from bovine ovarian follicles during atresia

**DOI:** 10.1186/1471-2164-15-40

**Published:** 2014-01-18

**Authors:** Nicholas Hatzirodos, Katja Hummitzsch, Helen F Irving-Rodgers, Margaret L Harland, Stephanie E Morris, Raymond J Rodgers

**Affiliations:** 1 Research Centre for Reproductive Health, Discipline of Obstetrics and Gynaecology, School of Paediatrics and Reproductive Health, Robinson Institute, University of Adelaide, Adelaide 5005SA, Australia; 2 Current Address: School of Medical Science, Griffith University, Gold Coast 4222 QLD, Australia

**Keywords:** Ovary, Microarray analysis, Bovine, Granulosa cells, Atresia, Follicles

## Abstract

**Background:**

The major function of the ovary is to produce oocytes for fertilisation. Oocytes mature in follicles surrounded by nurturing granulosa cells and all are enclosed by a basal lamina. During growth, granulosa cells replicate and a large fluid-filled cavity (the antrum) develops in the centre. Only follicles that have enlarged to over 10 mm can ovulate in cows. In mammals, the number of primordial follicles far exceeds the numbers that ever ovulate and atresia or regression of follicles is a mechanism to regulate the number of oocytes ovulated and to contribute to the timing of ovulation. To better understand the molecular basis of follicular atresia, we undertook transcriptome profiling of granulosa cells from healthy (n = 10) and atretic (n = 5) bovine follicles at early antral stages (< 5 mm).

**Results:**

Principal Component Analysis (PCA) and hierarchical classification of the signal intensity plots for the arrays showed primary clustering into two groups, healthy and atretic. These analyses and size-frequency plots of coefficients of variation of signal intensities revealed that the healthy follicles were more heterogeneous. Examining the differentially-expressed genes the most significantly affected functions in atretic follicles were cell death, organ development, tissue development and embryonic development. The overall processes influenced by transcription factor gene *TP53* were predicted to be activated, whereas those of *MYC* were inhibited on the basis of known interactions with the genes in our dataset. The top ranked canonical pathway contained signalling molecules common to various inflammatory/fibrotic pathways such as the transforming growth factor-β and tumour necrosis factor-α pathways. The two most significant networks also reflect this pattern of tissue remodelling/fibrosis gene expression. These networks also contain molecules which are present in the canonical pathways of hepatic fibrosis/hepatic stellate cell activation and transforming growth factor-β signalling and were up regulated.

**Conclusions:**

Small healthy antral follicles, which have a number of growth outcomes, exhibit greater variability in gene expression, particularly in genes associated with cell division and other growth-related functions. Atresia, on the other hand, not only involves cell death but clearly is an active process similar to wound healing.

## Background

The function of the ovary is to produce and release oocytes to be fertilised, leading to the production of offspring. Oocytes develop within ovarian follicles which in most mammals are formed during fetal life. These primordial follicles consist of an oocyte arrested in meiosis, and therefore not capable of mitosis. The oocyte is surrounded by a single layer of inactive pregranulosa cells [1]. These primordial follicles comprise the ‘ovarian reserve’ from which a number of follicles are activated each day to commence growth and maturation. During this process of folliculogenesis, the oocyte enlarges substantially, pregranulosa cells differentiate into granulosa cells and replicate, and a large fluid-filled antrum develops in the middle of the follicle [2]. The growth of antral follicles is largely under the influence of Follicle-Stimulating Hormone (FSH) [3]. During follicle growth granulosa cells produce increasingly more of the hormone oestradiol. After the surge release of Luteinising Hormone (LH) from the anterior pituitary gland which results in ovulation of the oocyte, the remaining granulosa cells of the follicle wall transform into luteal cells of the corpus luteum and produce progesterone [4]. Hence both the numbers and maturation of granulosa cells in any given follicle are important and both processes are regulated by gonadotrophic hormones from the anterior pituitary.

In mammals, the number of primordial follicles far exceeds the numbers that ovulate over a lifetime. For example in humans, millions of primordial follicles are formed in the fetal gonad but only about 500 will be ovulated [5]. Since the numbers of follicles at menopause is practically nil [5], the vast majority of follicles undergo atresia and regress. The incidence of follicular atresia [6] is a normal process of ovarian function and its occurrence across species appears to have increased, with the evolution of viviparity in which a reduced number of female gametes are required when compared to mass-spawning species. Atresia in any species can regulate the number of oocytes ovulated and contribute to the timing of ovulation in a reproductive cycle.

The process of atresia in follicles large enough to have developed an antral cavity is characterised initially by death of the mural granulosa cells with the presence of pyknotic nuclei followed by loss of these layers into the antrum [7]. The entire follicle wall then begins to breakdown at the basal lamina and inflammatory cells migrate from the surrounding stromal theca layers, phagocytosing remnants of the granulosa cells and eventually the oocyte. Atresia leads eventually to death of all the granulosa cells within a follicle. The cell death processes can involve apoptosis, necrosis, autophagy and cornification, and any of the major cell types of the follicle can be involved, depending upon the stage of follicular development when atresia occurs [8]. Atresia also involves active cellular processes including macrophage infiltration, phagocytosis, migration of fibroblasts from the theca and the production of collagen. Interestingly, these are some of the processes also observed in wound healing [9,10].

We hypothesise that apart from cell death, other signalling and pathways will be associated with the process of atresia. Therefore, to advance our knowledge of atresia we undertook transcriptome profiling of granulosa cells from small antral follicles before and during atresia. There have been several studies published, which investigate granulosa gene expression in developing bovine antral follicles by microarray [11-14]. Evans et al [11] studied granulosa from small follicles using self–generated arrays of approximately 1,300 genes. Other studies have focused on follicles at larger sizes, comparing follicles with differences in oestradiol production due to selectively accelerated development (dominance). Here we examined individual morphologically-classified healthy (n = 10) and atretic (n = 5) follicles at the small antral stage of less than 5 mm in diameter, prior to size deviation due to dominant selection. The Bovine Affy arrays we used contain more than 11,000 annotated genes, thereby expanding the power to reveal networks and pathways involved in follicle regression. The healthy follicles were further classified into two phenotypes based upon the shape of the basally-situated granulosa cells, as either columnar or rounded [15]. These follicle types also differ in the quality of their oocytes when cultured *in vitro*[16]. The atretic follicles were of the type called antral atretic [7,8]. This is the classic type of atresia commonly observed across species in which the antrally-situated granulosa cells are the first to undergo cell death.

## Results and discussion

**Figure 1:**
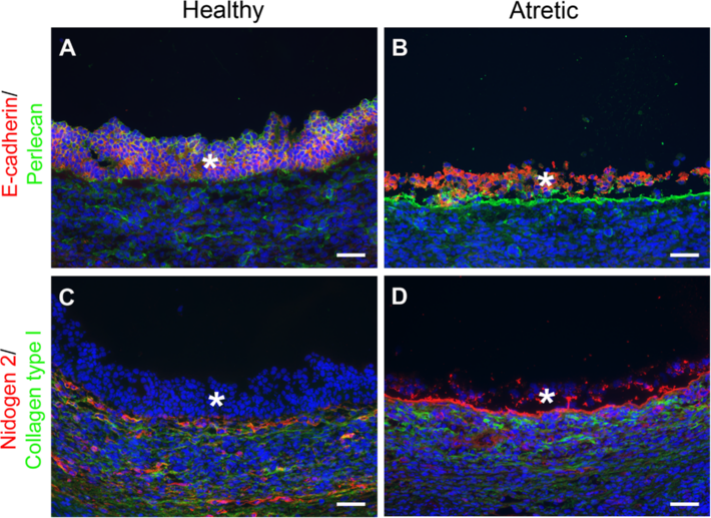
**Localisation of E-cadherin, perlecan, nidogen 2 and collagen type I in small healthy and atretic follicles. (A, B)** E-cadherin (red) is restricted to the membrana granulosa and is strongly expressed in atretic follicles. Perlecan (green) marks the follicular and sub-endothelial basal lamina and is expressed in the cytoplasm of granulosa cells of healthy and atretic follicles. **(C, D)** Nidogen-2 (red) is localised to the basal lamina of both follicle types, but is only expressed in the granulosa layer of small atretic follicles. Collagen type I (green) is not localised to the granulosa cells. It is restricted to the thecal and medullar stroma. The star indicates the granulosa layer. Bar = 50 μm.

In this study we have identified major differences in gene expression pathways and networks that develop in granulosa cells of small antral follicles during the process of atresia. To achieve this, granulosa cells from small healthy (3.1 ± SEM 0.2 mm diameter; n = 10) and atretic (4.2 ± 0.5 mm; n = 5) follicles were selected for the microarray gene expression analysis. To ensure that the granulosa cells isolated were not contaminated with any thecal cells, no follicles with more than a 1% level of expression of *CYP17A1* found in thecal samples were included. *CYP17A1* is expressed exclusively in thecal cells [17]. We also validated that our microarray analyses could detect differentially-expressed genes here by immunohistochemistry and elsewhere [18] by real time reverse transcription polymerase chain reaction (real time RT-PCR). Table 1 shows the selected genes and their signal intensities and fold differences between healthy and atretic follicles. *CDH1*, the gene for the cell-cell adhesion molecule E-cadherin, and *NID2*, the gene for nidogen 2, were both increased in atretic follicles. By immunohistochemistry, the levels of both E-cadherin (Figure 1A, B) and nidogen 2 (Figure 1C, D) were elevated in the membrana granulosa of atretic follicles. Collagen type I was also examined by immunohistochemistry on the basis that *COL1A2* was elevated in atretic follicles. However, no collagen type 1 was detected in the membrana granulosa of healthy or atretic follicles but it was identified in the thecal layers at higher levels in atretic follicles. Collagen type I contains both α1 and α2 subunits and whilst *COL1A2* was elevated *COL1A1* was not. Thus expression of collagen type I could not be validated, but both *CDH1* and *NID2* were.

**Table 1:** Expression of selected genes from the microarray analysis used for validation by immunohistochemistry

**Gene symbol**	**Log **_**2 **_**mean signal intensity ± SD†**		**Fold increases in atretic follicles‡**
	**Healthy follicles**	**Atretic follicles**	
	**(n = 10)**	**(n = 5)**	
*CDH1*	6.5 ± 0.7	8.9 ± 1.4	4.3
*COL1A1*	7.9 ± 0.3	7.0 ± 0.7	-1.5
*COL1A2*	6.6 ± 0.2	8.7 ± 1.9	5.3
*NID2*	5.0 ± 0.6	9.2 ± 1.4	18.4

† SD = standard deviation.

‡ fold change calculated on non-log transformed data.

### Statistical analysis of differentially-expressed genes

**Figure 2:**
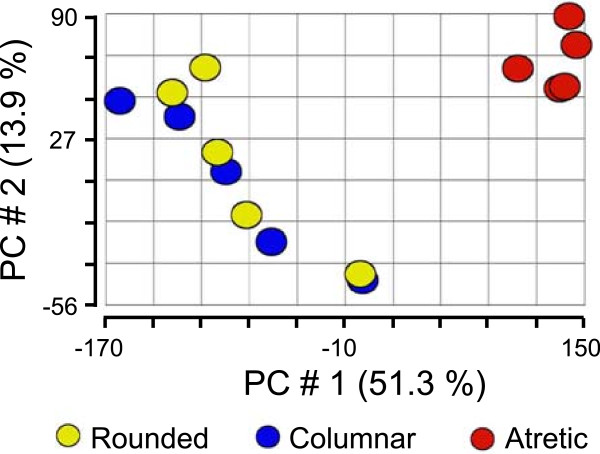
**Unsupervised PCA of arrays for small healthy (n = 5 rounded phenotypes in yellow and n = 5 columnar phenotypes in blue) and small atretic (n = 5, in red) follicles.** The graph is a scatter plot of the values for the first (X) and second (Y) principal components based on the correlation matrix of the total normalised array intensity data.

**Figure 3:**
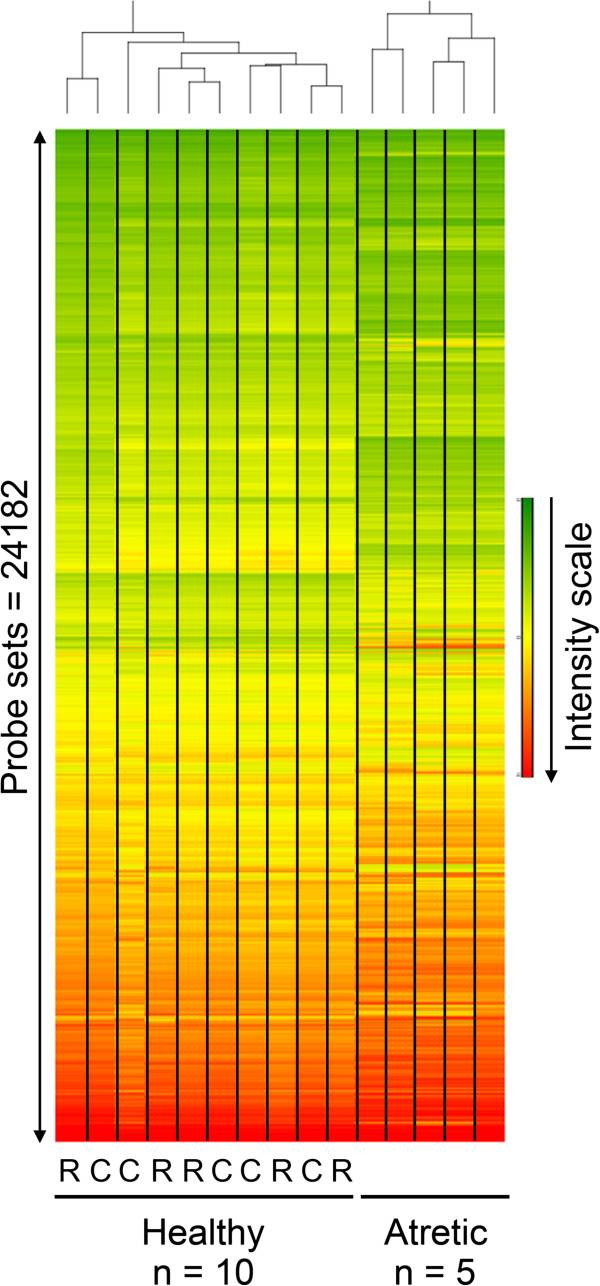
**Unsupervised hierarchical clustering across all probe sets (n = 24,182) for 15 arrays using the Euclidian dissimilarity algorithm with the average linkage method in Partek Genomics Suite.** The heatmap represents the distribution of normalised signal intensity, grouping by pattern similarity for both probe set and array. (R = rounded and C = columnar phenotypes).

Small healthy follicles were classified as either columnar (n = 5) or rounded (n = 5) on the basis of the shape of the basally-situated granulosa cells [15,16] as described in the Methods. PCA for the first three components (Figure 2) and hierarchical clustering (Figure 3) for the total number of probe sets (n = 24,182) of all arrays in this study were conducted. Neither of these unsupervised analytical methods separated the small healthy follicle arrays into the rounded and columnar groups, and in fact no genes were shown to be more than 2-fold differentially expressed between the two subgroups with a Benjamini-Hochberg False Discovery Rate (FDR) of *P* < 0.05 by ANOVA. Therefore, the small healthy follicle arrays were treated as a single group for further analyses (n = 10) and compared with the small atretic follicle group.

Before statistical analysis, PCA for all arrays revealed that the first principal component which accounted for 51% of the variation in the data, could separate the atretic and healthy follicle groups (Figure 2). Hierarchical classification of the signal intensity plot for these arrays similarly also showed primary clustering of the arrays into these two groups (Figure 3). The numbers of genes enriched 2-, 3-, and -4 fold by ANOVA in either healthy or atretic follicles are shown in Table 2. Follicular atresia was characterized by considerable change in the transcriptional processes of the granulosa cells as expected with over 22% of the total genes on the array being affected at least 2 fold or more (5,439 from 24,182). Four hundred and forty probesets were up regulated and 265 were down regulated more than 4 fold in atretic follicles relative to small healthy follicles. Using thresholds of 3- and 4-fold differential-expression levels with *P* < 0.05 and < 0.005 respectively, then 1,595 and 690 differentially-expressed probe sets were identified, respectively (Table 2). The larger dataset (1,595 probe sets) was tabulated with gene and fold change details added as Table 3 (up regulated in atretic) and Table 4 (down regulated) and in Additional file 1: Table S1.

**Table 2:** Number of probe sets differentially expressed in atretic follicles with respect to healthy follicles

**False discovery rate**	**Fold change**	**Up regulated**	**Down regulated**	**Total**
*P* < 0.05	>2	2573	2866	5439
	>3	824	771	1595
	>4	440	265	705
*P* < 0.005	>2	2397	2736	5133
	>3	801	732	1533
	>4	433	257	690

Determined by ANOVA and the step-up Benjamini Hochberg False Discovery Rate method for multiple corrections using Partek Genomics Suite Software.

**Table 3:** **Genes which were up regulated in small atretic follicles with respect to healthy follicles**^**†**^

**Gene symbol**	**Fold change**	**Gene symbol**	**Fold change**	**Gene symbol**	**Fold change**
**Cell Cycle and DNA replication**					
*CDKN1C*	40.2	*RGCC*	5.1	*IFITM3*	3.3
*MICAL1*	8.9	*TOP1*	5.1	*DYNLT3*	3.1
*CDC37L1*	5.9	*RAD52*	3.5	*BAZ1A*	3.0
*TOPORS*	5.7	*KLC1*	3.4		
**Cell Death**					
*TNFRSF12A*	10.4	*PHLDA1	4.7	*TNFRSF25*	3.4
*CFLAR*	6.0	*PDCD4*	3.9	*TNFRSF1A*	3.2
*TNFRSF6B*	5.9	*FAIM*	3.8	*PDCD10*	3.1
**Cell Morphology**					
*MAP1LC3C*	16.7	*APBB1IP*	4.6	*SNTB2*	4.0
*CAPG*	11.1	*MAP2*	4.5	*MTMR3*	3.8
*TAGLN*	10.8	*CNN1*	4.4	*LMNA*	3.5
*DBNDD2*	9.2	*AIF1L*	4.4	*ABLIM1*	3.5
*NDRG1*	9.0	*VCL*	4.3	*FHOD3*	3.4
*SYNE1*	7.6	*WASL*	4.3	*MARCKSL1*	3.2
*CLDN5*	6.3	*CDH1*	4.3	*MAP9*	3.2
*SVIL*	6.1	*ACTA2*	4.3	*MICAL2*	3.1
*MICALL2*	6.1	*TAGLN2*	4.3	*ELMO1*	3.1
*FILIP1L*	5.6	*FSCN1*	4.2	*ELMO2*	3.1
*ARPC1B*	5.5	*FNBP1L*	4.1	*ARF5	3.1
*FERMT2*	5.4	*TLN1*	4.1	*TPGS1*	3.0
*GSN*	5.3	*ACTN1*	4.1	*B9D2*	3.0
*TUBB6*	5.1	*DSTN*	4.0	*FBLIM1*	3.0
*CAV1*	4.6	*RILPL2*	4.0		
**Cytokines, Hormones and Receptors**					
*PDGFRA*	26.5	*IGF2R*	6.2	*CD302*	3.7
*SCG2*	24.4	*ELTD1*	6.1	*JAG1*	3.6
*CTGF*	23.4	*TSPO*	5.7	*IL10RA*	3.6
*DKK3*	17.4	*IGFBP6*	5.1	*PTPRK*	3.6
*XCL1*	16.7	*IGF2*	5.0	*PLXND1*	3.6
*SPP1*	14.1	*TGFBR2*	5.0	*CMTM3*	3.5
*OLR1*	11.7	*MIA3*	4.6	*IL17RA*	3.5
*MDK*	9.9	*IFI30*	4.5	*NR4A2*	3.5
*IL18*	8.9	*IGFBP5*	4.3	*ANGPTL4*	3.4
*PRICKLE1*	8.2	*EFNA4*	4.2	*EFNA5*	3.3
*BAMBI*	7.6	*NR2F1*	4.1	*GPRC5B*	3.3
*NDP*	7.6	*ADM*	3.9	*GPR155*	3.3
*GNG2*	7.3	*PLXNB2*	3.8	*ACVR1*	3.1
*PLAUR*	6.7	*PVRL2	3.8	*SCG5*	3.1
*BMP2*	6.6				
**Directional Cell Growth**					
*SLITRK2*	4.3	*ROCK2*	3.8	*ROCK1*	3.3
**Extracellular Matrix and Synthesis**					
*SPOCK2	23.8	*TNFAIP6*	7.1	*COL18A1*	4.0
*NID2	18.5	*LOXL4*	6.5	*FN1	3.9
*LEPREL1	12.8	*LUM*	6.1	*FBN1*	3.8
*LAMB1*	12.0	*COL3A1*	5.5	*COL4A5*	3.5
*DCN*	11.1	*COL1A2*	5.3	*SPON2*	3.5
*MGP*	9.5	*PCOLCE2*	4.6	*SDC4*	3.4
*SDC2*	9.0	*COL4A1*	4.5	*COL12A1*	3.4
*FBLN2*	8.7	*AGRN*	4.1	*COL6A1*	3.2
*COL5A2*	8.3				
**Intercellular and Cell to Matrix Adhesion**					
*KRT18*	62.4	*CLDN1*	10.2	*MAGI3*	4.6
*KRT8*	53.2	*CD9*	7.1	*AMIGO2*	4.5
*PTX3*	24.1	*PCDH7*	7.0	*ANTXR2*	3.7
*THBS2*	23.9	*FLNB*	6.6	*SPARC*	3.6
*LGALS3*	22.9	*MAGI1*	6.0	*CADM1*	3.3
*CD24	17.1	*VMP1*	5.3	*JAM3*	3.2
*CYR61*	14.1	*CLDN11*	4.9	*CCDC80*	3.0
*CALD1*	11.6				
**Ion Transport**					
*KCNMB4*	5.7	*CACNB3*	4.0	*KCTD10*	3.8
*GPM6A*	4.8	*CLCA4*	3.9	*SEPP1*	3.7
*CLIC4*	4.5	*SCN5A*	3.9	*KCNJ2*	3.7
**Protein Trafficking**					
*ANXA1*	10.9	*SCG3*	4.0	*PLEKHB2*	3.6
*GABARAPL1*	6.2	*CHMP5*	3.7	*SCAP*	3.4
*GIT2*	4.4	*MVP*	3.7	*PLEKHG2*	3.3
*SH3RF1*	4.0				
**Proteolysis or Inhibition**					
*TIMP1*	68.9	*CAST*	6.7	*CTSZ*	3.5
*PLAT*	26.5	*ADAMTS1*	6.1	*FBXO32*	3.3
*SERPINE1*	19.3	*C1S*	5.9	*TIMP2*	3.3
*PRSS23*	10.9	*CTSH*	4.7	*CTSK*	3.2
*CSTB*	9.3	*MMP11*	4.4	*CTSB*	3.2
*RELN*	7.7	*CTSS*	4.4	*KLHL36*	3.2
*MMP23B*	7.7	*CFD*	3.8	*SERPINB2*	3.1
*SERPINB8*	7.4	*PSMB1*	3.5	*LYZ*	3.0
**RNA Processing**					
*PRPF38B*	7.1	*RBM25*	3.9	*SNRNP200*	3.2
*RBMS2*	6.3	*DDX5*	3.7	*HNRNPH3*	3.2
*RBM24*	4.6	*SYNCRIP*	3.6	*CPEB4*	3.2
*RBM17*	4.5	*DDX42*	3.6	*EBNA1BP2*	3.1
*TDRD7*	4.3	*INTS7*	3.3	*GEMIN8*	3.1
*REXO2*	4.0	*CWC22*	3.2		
**Transcription Regulation**					
*ANKRD1*	48.9	*TAX1BP3*	4.8	*LEF1*	3.4
*FOSL2*	11.7	*MLL3	4.7	*TGIF1*	3.4
*EGR1*	11.3	*MTPN*	4.6	*MPHOSPH8*	3.4
*ATF3*	9.7	*EPAS1*	4.6	*MORF4L2*	3.3
*BCL6*	8.6	*FOXP1*	4.5	*MAFK*	3.3
*ATRX*	8.4	*ING4*	4.4	*CSRNP1*	3.3
*ID1*	7.4	*GTF2F2*	4.4	*LARP7*	3.3
*USF1*	6.8	*SMAD3*	4.3	*GTF2E2*	3.3
*KLF6*	6.5	*STAT3*	4.3	*GTF2H4*	3.3
*RBFOX2*	6.5	*SERTAD1*	4.3	*BASP1*	3.3
*JUN*	6.4	*HHEX*	4.2	*LEO1*	3.3
*CEBPD*	6.0	*ZNF398*	4.2	*TCF3*	3.3
*ID3*	5.8	*HMBOX1*	4.0	*ZMIZ2*	3.2
*TCF7L2	5.8	*PDLIM1*	3.9	*NFKBIA*	3.2
*HDAC7*	5.8	*ZFP36L1*	3.9	*ZC3H8*	3.2
*NMI*	5.7	*JUNB*	3.9	*OSTF1*	3.2
*SNAI2*	5.7	*E4F1*	3.9	*CIR1*	3.2
*ZNF292*	5.7	*ZNF281*	3.8	*ZNHIT3*	3.1
*LITAF*	5.6	*FOS*	3.7	*GPS2*	3.1
*CITED2*	5.6	*FHL2*	3.7	*CSDC2*	3.1
*CTR9*	5.5	*CYLD*	3.7	*KHDRBS1*	3.0
*CEBPD	5.4	*TRANK1*	3.6	*ILF3*	3.0
*KANK2*	5.1	*FOXO1*	3.6	*ANKRD10*	3.0
*NFIL3*	5.0	*CPEB2	3.6		
*JARID2*	4.9	*SCAND1*	3.5		
*MXI1*	4.9	*HDAC5*	3.5		
**Translation Regulation**					
*MRPS28*	3.6	*EIF4G3*	3.2	*EIF2AK4*	3.2
*EIF2AK4*	3.5				
**Transport**					
*APOD*	37.5	*SLC25A29*	4.2	*RBP1*	3.4
*STAR*	22.5	*STAU1*	4.1	*TCN2*	3.4
*SLC17A5*	9.0	*VPS13B*	4.1	*SLC25A17*	3.4
*FABP5*	8.9	*STEAP1*	4.0	*CYTH3*	3.4
*A2M*	8.1	*RABEP1*	3.9	*SYT11*	3.2
*NNAT*	7.8	*ABCB1	3.8	*FABP4*	3.1
*SLC40A1*	7.2	*CRABP2*	3.7	*AP1S2*	3.1
*VAT1*	6.8	*STARD3NL*	3.7	*NPC2*	3.0
*SLC39A8*	5.2	*ANKH*	3.6	*SNX9*	3.0
**Other Enzymes**					
*VNN1	15.1	*PNMT*	4.1	*RAB31*	3.2
*PDK4	9.7	*HSP90AA1*	4.0	*PTPRN2*	3.2
*GFPT2*	7.8	*RAP2C*	4.0	*RIOK2*	3.2
*QSOX1*	5.9	*SETD7*	4.0	*RNF20*	3.2
*DDAH2*	5.5	*CHST2*	4.0	*LHFPL2*	3.2
*GLRX*	5.4	*SMS*	3.8	*PTPN21*	3.2
*PLSCR4*	5.4	*NT5E*	3.8	*RAB7A*	3.1
*ENPP5*	4.9	*MARCKS*	3.7	*DDAH1*	3.1
*PYGL*	4.9	*GDPD1*	3.6	*PPIE*	3.1
*CA2*	4.8	*DUSP7*	3.6	*ARF5*	3.1
*RPIA*	4.7	*EGLN1*	3.5	*RBKS*	3.1
*PELI1*	4.7	*ZNRF1*	3.4	*UST*	3.1
*MYCBP2*	4.6	*NAGK*	3.4	*ENO2*	3.1
*CPT1A*	4.5	*MGST2*	3.4	*RNF8*	3.1
*GEM*	4.4	*B4GALT5*	3.4	*PLCL2*	3.0
*PIGT*	4.3	*RAB22A*	3.3	*DPH5*	3.0
*DUSP1*	4.3	*ALDH1A1*	3.3	*IP6K2*	3.0
*PXDN*	4.2				
**Other Signalling**					
*RND3	35.7	*EFHD2*	5.8	*SHISA2*	3.9
*DCLK1	19.5	*JAK1*	5.7	*RALA*	3.9
*GAL*	10.6	*VAV2*	5.6	*CSNK2A1*	3.7
*POSTN*	9.9	*INSIG2*	5.4	*BMPER*	3.7
*ARHGEF11*	9.9	*WDR44*	5.0	*ARHGAP32*	3.5
*TP53INP1*	8.9	*STK17A*	5.0	*S100A11*	3.3
*LMTK2*	8.1	*PTPN5*	4.6	*UACA*	3.2
*SH3BP5*	7.5	*WHSC1L1*	4.5	*ERBB2*	3.2
*GIMAP8*	7.5	*IRAK1BP1*	4.4	*FYN*	3.2
*SH3KBP1*	7.4	*CFH*	4.4	*CORO1A*	3.2
*ARHGEF3*	7.1	*IFITM1*	4.3	*BCAR1*	3.1
*RSPO3*	7.1	*PIK3CA*	4.3	*DOCK5*	3.1
*CD200*	7.0	*DAB2*	4.3	*PIK3CD*	3.1
*PLIN2*	6.6	*CSNK1G2*	4.2	*STK38L*	3.1
*PKIB*	6.5	*MERTK*	4.1	*MAP2K3*	3.1
*FBXO33*	6.2	*RASA2*	4.1	*TYRO3*	3.1
*CD55*	6.2	*PIM1*	4.0	*CARD10*	3.1
*ERRFI1*	6.1				
*CD99*	6.0				
*DAPP1*	5.8				
**Other**					
*C8orf4*	37.2	*RCN2*	4.4	*CBLB*	3.6
*SATL1*	15.2	*KIAA1598*	4.4	*ASCC2*	3.5
*WDFY4*	11.1	*FAM126B*	4.4	*FAM32A*	3.5
*PLXDC2*	9.1	*ZNF317*	4.2	*OCIAD2*	3.5
*LUC7L3*	8.9	*C1orf35*	4.2	*SAFB2*	3.5
*ZNF462*	7.5	*RASAL2*	4.2	*ALKBH4*	3.4
*NTN4*	7.3	*AKAP8L*	4.1	*TSC22D3*	3.4
*KIAA0408*	7.2	*DENND3*	4.1	*IRGQ*	3.4
*RSRC2*	7.1	*C1QTNF6*	4.1	*AUTS2*	3.4
*C10orf10*	7.0	*CCDC85B*	4.1	*GATSL2*	3.4
*PHF3*	6.4	*ARHGEF2*	4.0	*COMMD4*	3.4
*SDE2*	6.3	*MAP1B*	4.0	*MLLT11*	3.3
*BTBD3*	6.1	*C3orf19*	4.0	*ZFYVE1*	3.3
*TMEM14A*	5.9	*CUL9*	4.0	*PPFIBP1*	3.3
*ODF2L*	5.8	*MAP7D1*	3.9	*FAM129B*	3.3
*MXRA8*	5.8	*MTA3*	3.9	*FLRT2*	3.3
*GLIPR2*	5.6	*LIMCH1*	3.9	*PRRC2C*	3.2
*NDRG4*	5.6	*KLHL24*	3.9	*GPN3*	3.2
*TMEM176A*	5.4	*CXorf26*	3.9	*MESDC1*	3.2
*MXRA5*	5.3	*SHF*	3.9	*ARMCX3*	3.2
*CRYAB*	5.1	*YPEL5*	3.9	*SNN*	3.2
*C7orf41*	4.9	*PQLC3*	3.9	*KIAA0922*	3.2
*ZNF608*	4.9	*TMTC2*	3.9	*TCP11L2*	3.2
*KLHL28*	4.9	*HECA*	3.8	*ANKRD11*	3.2
*PLEKHO1*	4.8	*C9orf3*	3.8	*C1orf212*	3.1
*TACC2*	4.8	*LRCH1*	3.8	*TMEM176B*	3.1
*GOLPH3L*	4.8	*DPH3*	3.7	*SYAP1*	3.1
*ERRFI1*	4.7	*CD83*	3.7	*METRNL*	3.1
*SRP14*	4.7	*CTTNBP2NL*	3.7	*FAM160A2*	3.1
*ZDHHC23*	4.7	*TES*	3.7	*MEF2BNB*	3.1
*EML1*	4.7	*GLTSCR2*	3.7	*C5orf30*	3.1
*ANXA2*	4.7	*CLU*	3.6	*ATG14*	3.1
*ZNF521*	4.6	*LHFP*	3.6	*BOD1L1*	3.1
*PDLIM4*	4.6	*PKIG*	3.6	*DEFB4A*	3.0
*KIAA0232*	4.4	*LSG1*	3.6	*C17orf101*	3.0
*C12orf75*	4.4				

Differentially regulated genes (> 3 fold, *P < 0.05*) were annotated based on the Entrez Gene database. Genes are listed in descending order of fold change within each functional category.

† Benjamini-Hochberg post-hoc test for multiple corrections following one way ANOVA.

* Indicates genes determined from the Partek analysis based on the Affymetrix annotations which were not assigned identities by IPA.

**Table 4:** Genes which were down regulated in small atretic follicles with respect to healthy follicles†

**Gene symbol**	**Fold change**	**Gene symbol**	**Fold change**	**Gene symbol**	**Fold change**
**Cell Cycle**					
*HAUS4*	9.7	*CASC5*	4.1	*FAM83D*	3.4
*KIFC1*	7.2	*PTTG1	4.0	*CDCA2*	3.3
*TOP2A*	6.3	*BUB1*	3.9	*KIF23*	3.3
*HJURP*	5.6	*SKA3*	3.9	*CENPF*	3.3
*CDCA8*	4.6	*TPX2*	3.8	*MCM10*	3.3
*MDC1*	4.5	*SGOL1*	3.8	*CDCA3*	3.3
*RAD51AP1*	4.5	*CDC20*	3.8	*OIP5*	3.2
*CENPE*	4.5	*GINS3*	3.7	*ASPM*	3.2
*ANAPC5*	4.4	*ASF1B*	3.7	*NCAPH*	3.2
*NUSAP1*	4.3	*CDC6*	3.7	*MAD2L1*	3.1
*MYO1D*	4.3	*ERCC6L*	3.6	*CKAP2*	3.1
*KLC2*	4.3	*CCNB1*	3.6	*CENPA	3.1
*BUB1B*	4.2	*PRCC*	3.5	*AURKB*	3.0
*CNNM2*	4.2	*CENPN*	3.5		
**Cell Death**					
TRIB2	6.5	RIPK3	4.8		
**Cell Morphology**					
*CKAP2L*	4.7	*SRGN*	4.0	*MAPT*	3.3
*PLP1*	4.6	*HMMR*	3.8	*PRC1*	3.3
*JAKMIP1*	4.6	*RSPH9*	3.7	*TNNC1*	3.2
*TMEM138*	4.5	*SYNPO*	3.6	*PHACTR1*	3.1
*ECT2*	4.2	*LEMD2*	3.5	*MYO5C*	3.0
*TMEM216*	4.0	*CDH26*	3.5		
**Cytokines, Hormones and Receptors**					
*FGFR2*	8.4	*INHA*	3.8	*CCR3*	3.2
*F2RL2*	5.9	*ADRA1A*	3.7	*CHRM4*	3.2
*VEGFA*	5.8	*IL7*	3.6	*P2RY10*	3.2
*RHO*	5.8	*CD5*	3.6	*NR5A1*	3.2
*IL17RE*	5.5	*GPR128*	3.6	*OPN1LW*	3.2
*LTA*	4.8	*GPR61*	3.6	*AHR*	3.2
*IL18R1*	4.6	*GPR77*	3.5	*IL20RA*	3.2
*FSHR*	4.4	*FGF10*	3.4	*HTR1D*	3.2
*HTR2A*	4.4	*CCL11*	3.4	*FGFBP1*	3.1
*AMH*	4.2	*OPCML*	3.4	*TSHB*	3.1
*GPRC5A*	4.2	*CCL25*	3.4	*ASGR1*	3.1
*OPRM1*	4.2	*CCL28*	3.4	*HEG1*	3.1
*INHBA*	4.2	*FIGF*	3.4	*IL1A*	3.1
*NTRK3*	4.1	*CD72*	3.3	*IGF1*	3.1
*FST*	4.0	*ABP1*	3.3	*CASR*	3.0
*FSHB*	4.0	*EDNRA*	3.3	*GMFG*	3.0
*IL21*	4.0	*ITGB3*	3.2	*HTR4*	3.0
*BMP15*	3.8	*NGFR*	3.2		
**Directional Cell Growth**					
*FAT1*	3.6				
**Extracellular Matrix and Synthesis**					
*LAMC2*	6.4	*COL6A6*	3.7	*COL10A1*	3.5
*EPYC*	4.3	*TRAM2*	3.6	*AMELX//AMELY	3.3
**Intercellular and Cell to Matrix Adhesion**					
*MUC15*	5.7	*CLDN6*	3.8	*DSG1*	3.1
*PRELP*	4.5	*CD33*	3.5	*PECAM1*	3.1
*MCAM	4.0	*SMAGP*	3.5	*EPDR1*	3.1
*GLYCAM1	3.9	*BOLA-DQ2	3.3	*CLEC4E*	3.0
*SELL*	3.9	*CD96*	3.2		
**Ion Transport**					
*TTYH1*	4.8	*KCNE1*	3.4	*SLC25A34*	3.1
*FXYD7*	4.3	*P2RX5*	3.2	*CACNA1D*	3.1
*KCNJ15*	3.5	*TRPM6*	3.2	*KCNIP2	3.0
**Protein Trafficking**					
INSIG1	3.8	VPS52	3.2		
**Proteolysis or Inhibition**					
*PTI	58.8	*TROAP*	3.5	*DBC1*	3.2
*CPXM2*	5.2	*MMP7*	3.4	*TASP1*	3.2
*TFPI*	5.1	*ACE2*	3.3	*PGA5*	3.1
*PRSS22*	4.4	*CPB1*	3.3	*SERPINI2*	3.1
*CUL7*	4.3	*SPPL2B*	3.2	*MMP3*	3.0
*KLK4*	4.1	*SPAG5*	3.2	*TRIM8*	3.0
*USP28*	3.8				
**RNA Processing**					
DCP1A	3.6	U2SURP	3.0		
**Transcription Regulation**					
*ZFHX3*	6.3	*HAND1*	3.8	*BCORL1*	3.2
*HOXB2*	4.6	*NFE2L2*	3.6	*NCOA6*	3.2
*ZNF385A*	4.5	*SP100*	3.6	*EHMT2*	3.2
*CCNT1*	4.5	*UHRF1*	3.5	*CC2D1B*	3.2
*SRCAP*	4.3	*BCOR*	3.5	*IRF2BP1*	3.2
*LDB1*	4.2	*RFX5*	3.4	*NSD1*	3.1
*POLR3D*	4.0	*NOTCH4*	3.4	*TFAP2A*	3.1
*ELK1*	4.0	*ASB12*	3.3	*ZBTB4*	3.1
*VSX1*	4.0	*NFIA*	3.3	*POU1F1*	3.1
*VGLL1*	3.8	*HOXD9*	3.2	*ZNF274*	3.0
**Translation Regulation**					
*YBX2*	4.9	*EEFSEC*	3.3	*EEF1A1*	3.1
*NARS*	3.3				
**Transport**					
*AQP1*	7.0	*SLC12A8*	4.0	*SLC8A1*	3.4
*TRPA1*	6.1	*SLC5A9*	4.0	*EPB42*	3.4
*ATP10A	6.1	*PDYN*	4.0	*SYNGR3*	3.3
*TNPO1*	6.0	*SLC24A1*	4.0	*SLC6A9*	3.2
*ALB*	5.7	*RHBG*	4.0	*AP4B1*	3.2
*SLC27A3*	5.5	*ATP13A2*	3.8	*GOSR1*	3.2
*SLCO2B1*	5.1	*KIF20A*	3.6	*ATP4A*	3.2
*APOB*	5.0	*STRA6*	3.6	*PLLP*	3.2
*MAL2*	4.6	*CLDN16*	3.5	*FLVCR2*	3.1
*SLC4A2*	4.5	*SLC7A1*	3.5	*SLC37A2*	3.0
*ATP2B4*	4.4	*GC*	3.4	*SLC13A2*	3.0
*ABCD1*	4.2	*ENSA*	3.4	*SLC38A11*	3.0
*SLC16A3*	4.1				
**Other Enzymes**					
*CYP19A1*	19.8	*CYP4F2*	4.2	*PNLIP*	3.4
*AOAH*	15.1	*PDE4D*	4.1	*PDE6G*	3.3
*PDK4	9.7	*GPT*	4.1	*TRNAU1AP*	3.3
*NOS2*	8.8	*PDP2*	3.9	*ALDH1L2*	3.3
*ISG20*	8.4	*DBT*	3.9	*PJA2*	3.3
*ALG3*	7.8	*LPO*	3.9	*WDR46*	3.3
*CHST8*	7.0	*ALOX12B*	3.8	*IPMK*	3.2
*HMGCS1*	6.9	*METTL7B*	3.7	*ST6GAL1*	3.2
*GCLC*	6.9	*IGL@	3.7	*ALG5*	3.2
*CA14*	6.8	*IGHG	3.7	*CA5B*	3.2
*UGT2B17*	6.5	*ETNK2*	3.6	*ACAD10*	3.2
*AKR1C3*	5.9	*PIPOX*	3.6	*DIO1*	3.2
*GYLTL1B*	5.7	*RNF168*	3.6	*ACSM1*	3.1
*SCD*	5.6	*LHPP*	3.6	*GSTM4*	3.1
*GPX3*	5.4	*CYP21A2*	3.6	*RSAD2*	3.1
*CYP2C19*	5.2	*NQO1*	3.6	*ACSM2A*	3.1
*GPX2*	5.0	*METTL21B*	3.5	*SH3GL2*	3.1
*HSD17B1*	4.8	*GCNT1*	3.5	*SEPT4*	3.1
*FHL3	4.7	*LPPR2*	3.5	*UBE2C*	3.1
*LPL*	4.6	*BCAT1*	3.5	*RBBP8*	3.1
*PLA2G1B*	4.5	*BBOX1*	3.5	*B3GNT3*	3.1
*PPP3CC*	4.5	*PNLIPRP2*	3.5	*DUSP14*	3.0
*PDE6A*	4.4	*METTL2A*	3.4	*GNA14*	3.0
*FMO2*	4.4	*P4HA2*	3.4	*CPS1*	3.0
*TYRP1*	4.3	*GALNT13*	3.4		
*CMBL*	4.2	*DCT*	3.4		
**Other Signalling**					
*IHH*	7.1	*MAP4K1*	4.0	*LAT*	3.3
*HLA-A*	6.2	*RIC3*	3.8	*PILRA*	3.3
*TBKBP1*	6.2	*CD84*	3.8	*GPSM3*	3.2
*HSPA1A	5.7	*SHCBP1*	3.6	*DNAJB1*	3.1
*TESPA1*	5.4	*GUCA1A*	3.6	*KIR2DL5A*	3.1
*JAK3*	5.1	*MTUS1*	3.4	*PEX11B*	3.1
*GUCY2F*	4.6	*DOK2*	3.4	*UPK1A*	3.1
*SKAP1*	4.5	*RPS6KA4*	3.3	*LY6G6C*	3.1
*RASGRP4*	4.2	*RGS3*	3.3	*FIGNL1*	3.0
*BCL9*	4.0				
**Other**					
*CSN2*	17.7	*EMID1*	4.3	*GIMAP7*	3.5
*MZB1*	10.1	*CYLC1*	4.3	*GUCA2A*	3.4
*STAC3*	6.8	*CCDC159*	4.3	*PLEKHF2*	3.4
*CCDC3*	6.3	*C21orf62*	4.2	*IFI44L*	3.4
*C9orf152*	5.9	*C1orf170*	4.2	*ODZ3*	3.4
*AGR2*	5.8	*APOBEC4*	4.2	*MICA*	3.3
*STAC*	5.6	*VWA8*	4.1	*TMEM139*	3.3
*EFHD1*	5.5	*PEAR1*	4.1	*UBN2*	3.3
*MTHFSD*	5.4	*MPDZ*	4.0	*C6orf47*	3.3
*NXPH2*	5.4	*IFIT2*	4.0	*Btnl1*	3.3
*SPEF1*	5.3	*ELMOD3*	4.0	*CXorf64*	3.3
*CRISPLD2*	5.2	*PLEK*	4.0	*DENND2D*	3.3
*KLHL29*	5.2	*SOWAHA*	3.9	*CHAC1*	3.3
*MEX3C*	5.0	*GCA*	3.8	*BIRC5*	3.2
*CAMP*	4.7	*LY9*	3.8	*CAPSL*	3.2
*KIAA0101*	4.6	*CCDC132*	3.7	*RSPH10B	3.2
*HRG*	4.6	*CRP*	3.6	*ANKRD17*	3.1
*KLHL33*	4.6	*C16orf53*	3.6	*RNFT1	3.1
*CEP85*	4.5	*HYOU1*	3.6	*HYDIN*	3.1
*PTGFRN*	4.5	*RASGEF1A*	3.6	*ASPHD1*	3.1
*TMIGD2*	4.5	*PIP*	3.6	*CCDC97*	3.0
*C6*	4.4	*OLFML1*	3.5	*CCDC43*	3.0
*WDR76*	4.4	*FAM178A*	3.5	*PA1	3.0
*MOB3B*	4.4	*TAGLN3*	3.2	*MLKL*	3.0
*RBL2*	3.8	*TMED6*	3.2	*WDR87*	3.0

† Differentially expressed genes (≥ 3 fold, *P <* 0.05) as annotated based on the Entrez Gene database and categorised by function using the Benjamini-Hochberg post-hoc test for multiple corrections following one way ANOVA.

*indicates genes determined from the Partek analysis based on the Affymetrix annotations which were not assigned identities by IPA.

### Variability of gene expression between follicles

**Figure 4:**
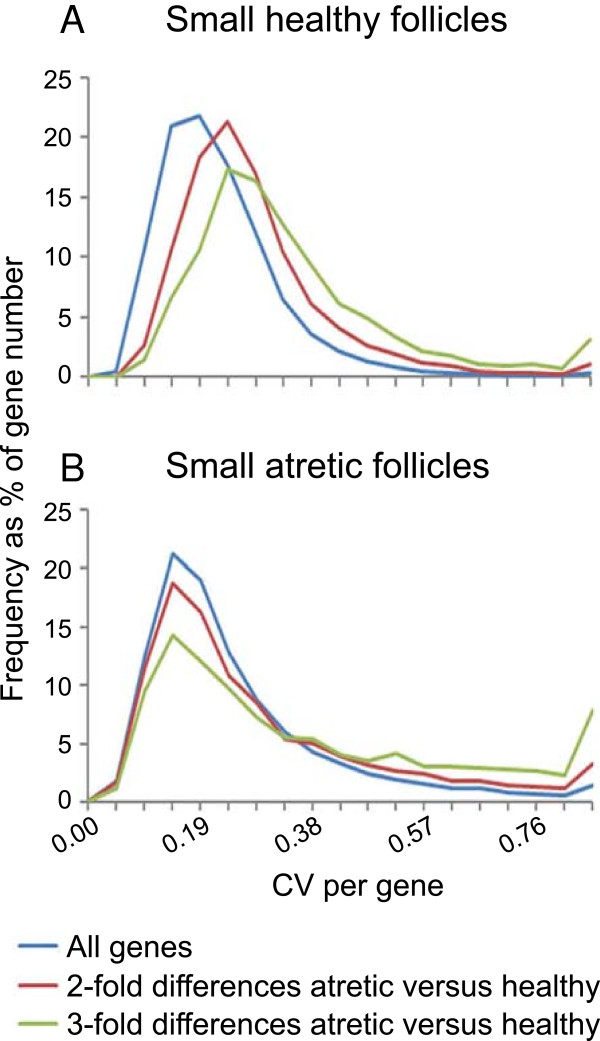
**Frequency coefficients of variation (CV) distributions in selected datasets from healthy follicles (n = 10) in A and atretic (n = 5) follicles in B.** ‘All genes’ include all the probe sets present on the array (n = 24,128). ‘2 fold’ or ‘3 fold’ or more represent all genes which were 2 fold or more (n = 5,475) or 3 fold or more (n = 1,596) differentially-regulated genes between healthy and atretic follicles using Partek Genomics Suite Software.

The PCA indicated that the healthy follicles were a more heterogeneous group than the atretic follicles and we examined this further. In other studies examination of the variably expressed genes has recently been used as a tool to identify differences in the pathways of different neurological diseases [19], therefore we applied a similar approach to our data. The coefficients of variation (SD/Mean × 100) for the healthy and the atretic follicles of each probe set were calculated and the size-frequency distribution plot for healthy and atretic follicles is shown in Figure 4. The healthy follicles (Figure 4A) show increasing gene variation with increasing fold difference for the subset of genes which are differentially regulated between healthy and atretic follicles, which is not seen in the atretic follicle group (Figure 4B). We investigated this variation further and identified the most highly variable genes in small healthy follicles. A group of 682 of the most variable probe sets in small healthy follicles, which had a coefficient of variation value of > 46.8%, was assembled and analysed by Ingenuity Pathway Analysis (IPA) and Gene Ontology (GO) enrichment analysis (Tables 5 and 6). Cell cycle regulation is the most common function associated with the highly variable gene dataset. Thirteen genes were associated with GO terms for this function and cyclin genes such as *CCNB1*, *CCNB2* and *CDK1* were represented in both analyses and in the top canonical pathways. The enrichment analysis produced a number of additional functionally-related gene groups associated with variable expression. These categories included; regulation of vascularity, extracellular matrix, energy metabolism, inflammation, cell migration and MAPK activity. Interestingly, there were 17 extracellular matrix genes found to be highly variable across our healthy follicle arrays, and several of them code for a number of collagen types (1α1, 1α2, 3α1, 4α3, 4α4 and 18). Energy metabolism was identified as an important process with an association of 13 genes from this variable group, particularly glucose metabolism via *ISR2*, *IGFBP2*, *PDK4* and *ASPSCR1*. Molecules known to promote angiogenesis in the ovary such as VEGF and angiopoietin, and an inhibitor thrombospondin, were also associated with our variable dataset.

**Table 5:** The most variable genes in small healthy follicles mapping to networks and pathways in IPA

	**Genes mapped from dataset**			
	**Symbol**	**Biological process**		
Network 1 †(Score = 42)	*PLSCR1, PLSCR4, heparanase, CTSL1, ATP2B1*	apoptosis		
	*UHRF1*	cell cycle regulation, mitosis		
	*TIMP1,*	matrix degradation		
	*EGFR,* *JUNB,* *FOSL2*	differentiation/maturation of granulosa cell through AP-1		
	*CYP19A1, CYP11A1,* *INHBA, BAMBI*	steroidogenesis, regulation of gonadotropin secretion/granulosa cell proliferation		
	*SOD3*	stress response		
	*ADM*	regulation of blood supply		
	*CLDN11*	maintenance of epithelial integrity		
	*VARS*	growth metabolism		
	*AHNAK*	actin cytoskeleton organisation, cell polarization		
Network 2 †(Score = 42)	*CCNB1, ESPL1, H1FX, H2AFX, BUB1,NCAPG, NCAPG2, SEPT4, S100A4, G3BP1*	cell cycle regulation, mitosis		
	*PSMD4, PSMD13, UBQLN1, PDIA4, HSP70*	protein recycling and folding		
	*PEG3*	apoptosis		
	*PRC1*	cell migration		
Canonical pathways	**Cell Cycle: G2/M DNA Damage Checkpoint Regulation, Mitotic roles of Polo-Like Kinases**		***P *** **value††**	
			**Fisher’s**	**B-H FDR**
1	*CCNB1,CCNB2,CDK1,GADD45A, RPRM, TOP2A, YWHAG*	cell cycle regulation, mitosis	3.2 × 10^-4^	5.8 × 10^-2^
2			3.9 × 10^-4^	5.8 × 10^-2^
3	**ATM Signalling**			
	*JUN, SMC2, GADD45A, H2AFX, CCNB2, CDK1, CCNB1*	response to DNA damage	8.2 × 10^-4^	8 × 10^-2^

†The network score is based on the hypergeometric distribution and is calculated with the right-tailed Fisher’s Exact Test. The score is the negative log of this *P* value.

†† Significance of association of genes with canonical pathways was determined by a right tailed Fisher’s Exact Test and the Benjamini-Hochberg False Discovery Rate (B-H FDR) for multiple comparisons. The variability of expression was determined by a frequency distribution of the coefficients of variation for probe sets across the arrays in the small healthy follicle group. The cut-off chosen was a coefficient of variation of > 46.8% (n = 10 arrays, n = 682 probe sets).

Gene symbols which are underlined indicate those genes which interact with a minimum of 4 other molecules within the dataset.

**Table 6:** **GO enrichment analysis of the most variable genes in granulosa cells from small healthy follicles using GOEAST**†

**Biological process**	**Molecular function**	**Cellular component**	**Symbol**	**Entrez gene name**
Regulation of vascularity *Angiogenesis* (*P* = 0.009)			*CYR61*	cysteine-rich angiogenic inducer
			*COL18A1*	collagen 18
			*THBS1*	thrombospondin 1
			*CXCR4*	chemokine receptor 4
			*ANGPT2*	angiopoietin 2
			*VEGFA*	vascular endothelial growth factor A
Energy metabolism *Glycogen biosynthetic process* (*P* = 0.044)	Insulin-like growth factor binding, two component sensor activity	microsome	*ISR2*	insulin receptor 2
			*EGFR*	epidermal growth factor receptor
			*HSP90AA1*	heat shock protein 90 kDa alpha (cytosolic), class A member 1
			*PDK4*	pyruvate dehydrogenase kinase, isozyme 4
			*IGFBP2*	insulin-like growth factor binding protein 2
			*CTGF*	connective tissue growth factor
			*CYR61*	cysteine-rich angiogenic inducer
			*MGST1*	glutathione transferase 1
			*CALR*	calreticulin
			*PTGES*	prostaglandin E synthase
			*PLC4*	phospholipase C, beta 4
			*OPTN*	optineurin
			*ASPSCR1*	alveolar soft part sarcoma chromosome region, candidate 1
Cell Cycle *Mitosis* (*P* = 2.3 × 10^-5^)		Condensed chromosome kinetochore, spindle	*CCNA2*	cyclin A2
			*CCNB1*	cyclin B1
			*CCNB2*	cyclin B2
			*UBE2*	ubiquitin conjugating enzyme 2
			*CDK1*	cyclin-dependent kinase 1
			*NUSAP*	nucleolar and spindle associated protein 1
			*SKA1*	spindle and kinetochore associated complex subunit 1
			*NUP85*	nucleoporin 85 kDa
			*CENPN*	centromere protein N
			*CENPO*	centromere protein O
			*ZW10*	kinetochore associated homolog
			*BUB1*	budding inhibited by benzimidazoles 1 homolog (yeast)
			*STK*	serine/threonine kinase
Extracellular matrix *Extracellular matrix organisation* (*P* = 0.01)	Heparin binding, extracellular matrix binding	Basement membrane	*COL1A1*	collagen type I, alpha 1
			*COL1A2*	collagen type I, alpha 2
			*COL3A1*	collagen type III, alpha 1
			*COL4A3*	collagen type IV, alpha 3
			*COL4A4*	collagen type IV, alpha 4
			*COL18*	collagen type XVIII
			*LAMB1*	laminin, beta 1
			*VCAN*	versican
			*FMOD*	fibromodulin
			*ADAMTS1*	ADAM metallopeptidase with thrombospondin type 1 motif, 1
			*ADAMTS6*	ADAM metallopeptidase with thrombospondin type 1 motif, 6
			*MMP2*	matrix metallopeptidase 2
			*TIMP1*	TIMP metallopeptidase inhibitor 1
			*OGN*	osteoinductive factor/osteoglycin
			*OSF-2*	osteoblast-specific factor 2
			*SPARCL1*	SPARC-like 1 (hevin)
			*ASPN*	asporin
Inflammation		Fibrinogen complex	*THBS1*	thrombospondin 1
*NOS biosynthesis* (*P* = 0.044)			*CALR*	calreticulin
*Antigen processing* (*P* = 0.026)			*BOLA-A*	major histocompatibility complex (MHC), class I, A
*TGFβ receptor signalling pathway* (*P* = 0.05)			*BOLA-N*	MHC class I antigen
*Fibrinolysis* (*P* = 0.09)				
Cell migration			*CXCR4*	chemokine receptor 4
*Axon guidance* (*P* = 0.0008)			*ABLIM1*	actin binding LIM protein 1
			*COL18*	collagen type XVIII
			*EFNA5*	ephrin A5
			*THBS1*	thrombospondin 1
			*EGFR*	epidermal growth factor receptor
			*TDGF*	teratocarcinoma-derived growth factor 1
			*F2RL1*	coagulation factor II (thrombin) receptor-like 1
			*INSR*	insulin receptor
MAPK activity (*P* = 0.002)			*THBS1*	thrombospondin 1
			*TDGF*	teratocarcinoma-derived growth factor 1
			*INSR*	insulin receptor

†The significance of association for the GO enrichment analysis was determined by the Benjamini-Yekuteli FDR method for multiple comparisons. The variability of expression was determined by a frequency distribution of the coefficients of variation for probe sets across the arrays in the small healthy follicle group. The cut-off chosen was a coefficient of variation of > 46.8% (n = 10 arrays, n = 682 probe sets).

The large variability of gene expression across healthy follicles is probably not unexpected since small growing follicles have a number of possible growth trajectories: 1. continued growth to become a dominant follicle, with the likelihood of a) ovulation or b) atresia, 2. continued growth as a subordinate follicle with atresia as the ultimate fate or 3. atresia at an earlier stage. Whether this variability reflects early commitment or predisposition of follicles to one of the three outcomes, or whether it reflects flexibility without a predetermined outcome is not clear at this stage. However, our identification of the pathways and genes involved is an important first step towards understanding the underlying mechanisms responsible for the growth and atresia of follicles.

### Pathway, network and upstream regulator analyses of healthy versus atretic follicles

**Figure 5:**
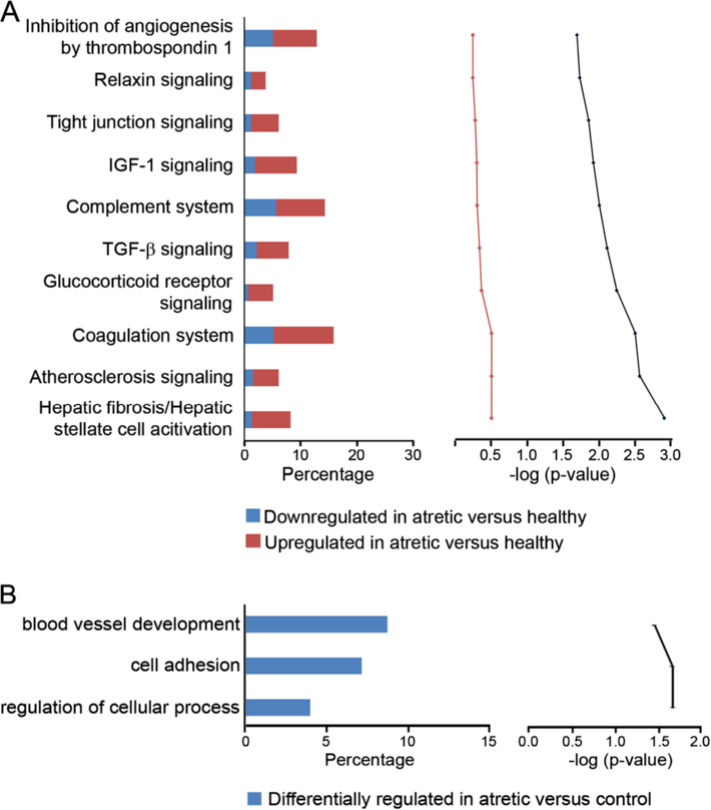
**Top canonical pathways mapped in Ingenuity Pathway Analysis (A) and GO terms classified under biological process (B) for a selected set of genes differentially regulated between healthy and atretic follicles.** In **A** the bar chart on the left represents the percentage of genes that map to each canonical pathway, showing those which are up regulated (in red) and down regulated (in blue) in atretic with respect to healthy follicles. The line chart on the right ranks these pathways derived for the same dataset, from the highest to lowest degree of association based on the value of a right-tailed Fishers exact *t* test (black), and the Benjamini-Hochberg False Discovery rate test for multiple comparisons (red) (top to bottom in graph on right). In **B** the significance of association was determined by the Benjamini-Yuketeli test for multiple comparisons. The bar chart indicates the percentage of genes that map to a GO term which are differentially regulated (in blue). Only those significantly enriched GO terms associated with a subset of genes of the most specific function were presented, to avoid terms which were too general and of limited value.

**Figure 6:**
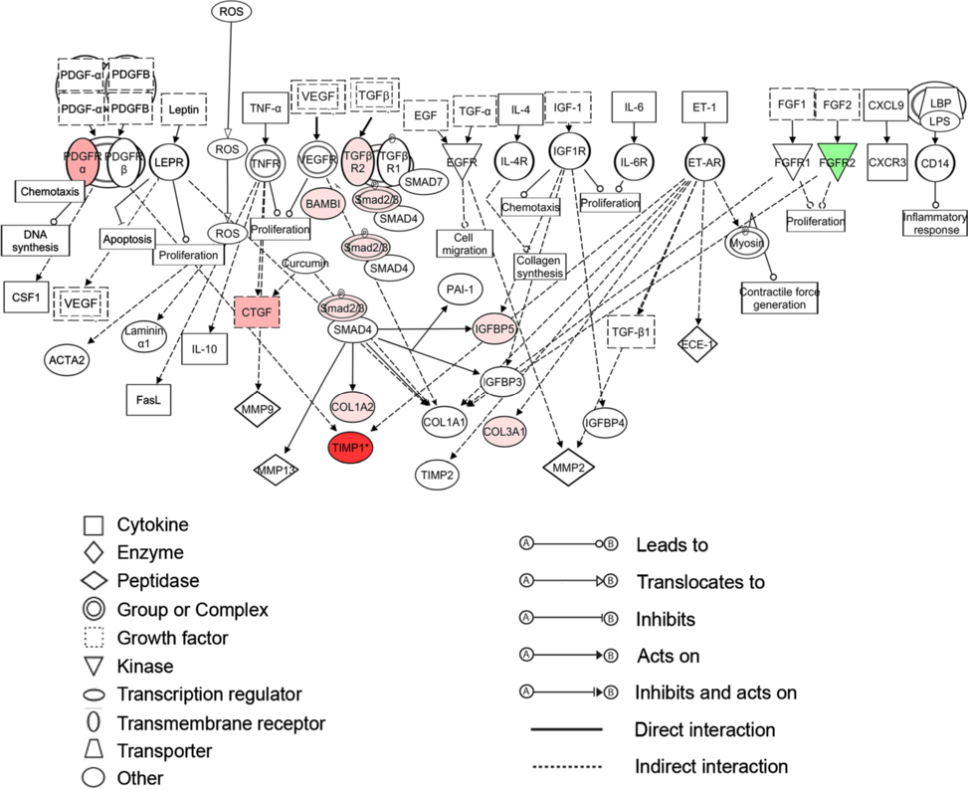
**The hepatic fibrosis/hepatic stellate cell activation canonical pathway in IPA.** Interactions between molecules are shown as explained in the legend, with focus molecules (those from our dataset) highlighted in colour, based on up (red) or down regulation (green) and increasing colour intensity with degree of fold change.

**Figure 7:**
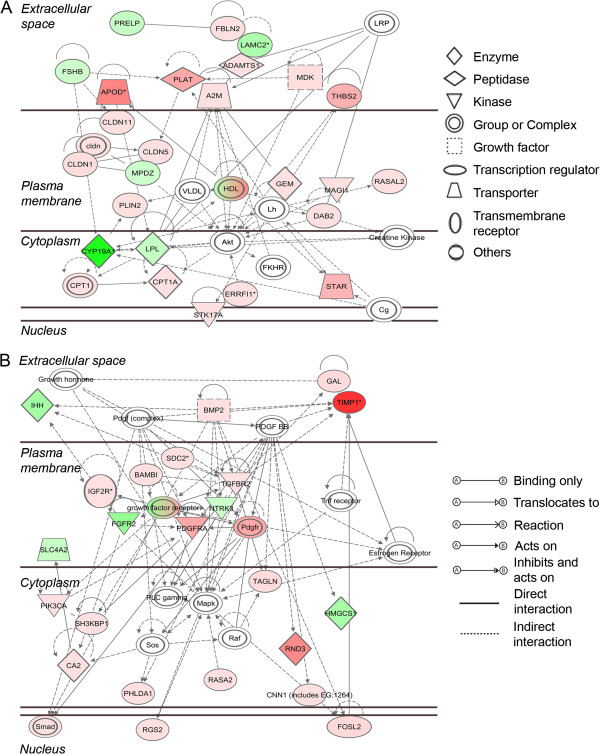
**Two statistically significant networks (A and B) produced by mapping differentially expressed genes between healthy and atretic follicles to molecules in the IPA database.** The networks are generated in IPA using triangle connectivity based on focus genes (those present in our dataset) and built according to the number of interactions between a single prospective gene and others in the existing network, and the number of interactions the prospective gene has outside this network with other genes as determined by IPA [20]. Interactions between molecules, and the degree and direction of regulation are indicated similarly as in Figure 6 with up (red) or down regulation (green) and increasing colour intensity with degree of fold change.

A set of 690 probe sets, which were more than 4-fold differentially regulated in signal intensity between atretic and healthy follicles with a FDR of *P* < 0.005, were subjected to pathway analyses in IPA. Of these, 456 were mapped to known identities in the Ingenuity Knowledge database and only 428 were eligible for network generation, due to the presence of replicate probe sets with specificity for the same gene on the chip. This group contained 288 probe sets which were up regulated in atretic with respect to healthy follicles and 140 which were down regulated. The most significantly affected functions associated with this dataset were found to be cell death, organ development, tissue development and embryonic development, which were all predicted to be negatively regulated in atretic follicles (Table 7). Upstream Regulator analysis revealed that the transcription factor genes *TP53*, *FOXO4* and *CEBPB* are predicted to be activated, whereas those of *RXRA*, *HNF1A* and *MYC* are inhibited on the basis of known interactions with the genes in our dataset (Table 8). The most significant canonical pathways represented in our analysis are shown in Figure 5. The top ranked canonical pathway (Figure 6), contains signalling molecules from our dataset which are common to various inflammatory/fibrotic pathways such as the transforming growth factor-β (TGFβ) (Additional file 2: Figure S1) and tumour necrosis factor-α (TNFα) pathways. The two most significant networks (Figure 7A and 7B) also reflect this pattern of tissue remodelling/fibrosis gene expression. These networks also contain molecules which are present in the canonical pathways of hepatic fibrosis/hepatic stellate cell activation and TGFβ signalling and are up regulated in our analysis and should therefore positively stimulate these networks. These include genes such as *THBS2*, *PLAT*, *BAMBI*, *TGFBR2*, *BMP2*, *SMAD3*, *FGFR2*, *PDGFRA* and *TIMP1*.

**Table 7:** Biological functions determined in IPA for genes differentially regulated between atretic and small healthy follicles

**Category**	**Functions annotation**	***P *** **Value**		**Bias-corrected z-score**	**Genes**
		**Fisher’s**	**(B-H) FDR**		
Cancer	tumorigenesis of organ	8.31E-04	1.57E-02	-2.907	*CAV1, CDKN1C, COL18A1, CYP19A1, CYR61, FST, GADD45A, ING4, JUN, MMP11, NDRG1, SMAD3, STAR, TGFBR2, TIMP1, VEGFA*
Cancer	hyperproliferation	5.94E-05	9.22E-03	-2.889	*AXL, BCL6, CAV1, CDKN1C, CEBPD, CYP19A1, FGFR2, FST, GPX3, IGF2R, ING4, MERTK, MGP, MXI1, NOS2, PLAUR, PLP1, POSTN, SERPINE1, SMAD3, SPP1, STAR, STAT3, TGFBR2, TIMP1, TOP1, VEGFA*
Cell Death	cell death of organ	1.32E-10	3.34E-08	-2.868	*A2M, AMH, ATF3, AXL, BCL6, BMP2, C8orf4, CAMP, CAST, CCNT1, CD200, CDKN1C, CFH, CFLAR, CLIC4, CTGF, CYP19A1, CYP2C9, DCN, DUSP1, EGR1, GAL, GCLC, GLRX, GNG2, GSN, GTF2F2, ID3, IER3, IGF2, IL18, JUN, KRT8, LGALS3, LTA, MDK, MERTK, MTPN, MZB1, NOS2, NTRK3, OPRM1, PIK3CA, PIM1, PLAT, PLAUR, PTPN5, RND3, SERPINA3, SERPINE1, SH3BP5, SMAD3, SPP1, STAR, STAT3, THBS2, TIMP1, TNFRSF6B, TOP2A, TP53INP1, VEGFA*
Cell-To-Cell Signalling and Interaction	activation of blood cells	5.83E-04	1.21E-02	-2.800	*AGRN, ANXA1, ANXA2, AXL, C6, CAMP, CD200, CFH, DUSP1, F2RL2, HDAC7, HLA-A, HRG, IGF2R, IL18, IL18R1, LTA, MERTK, NDRG1, NOS2, OPRM1, PDGFRA, PELI1, PLAT, PLP1, SMAD3, SPP1, STAT3, TNFRSF12A, TNFRSF6B, VEGFA, WASL*
Cancer	hyperplasia	2.82E-04	7.20E-03	-2.753	*AXL, CAV1, CDKN1C, CEBPD, CYP19A1, FGFR2, FST, GPX3, IGF2R, ING4, MGP, MXI1, NOS2, PLAUR, POSTN, SERPINE1, SPP1, STAR, STAT3, TGFBR2, TIMP1, TOP1, VEGFA*
Tissue Development	development of organ	1.95E-09	3.70E-07	-2.665	*ADAMTS1, ALB, AMH, ANKRD1, AQP1, ATF3, ATRX, AXL, BCL6, BMP2, CAMP, CAV1, CDKN1C, CFLAR, CITED2, CLDN1, CLDN11, COL18A1, COL1A2, COL3A1, CTGF, CTSH, CYP19A1, CYR61, DCN, EGR1, EPAS1, ERRFI1, FABP5, FGFR2, FOSL2, FOXP1, FSHB, FST, GAL, HHEX, HRG, ID1, ID3, IGF2, IGF2R, IGFBP5, IHH, INSIG2, JARID2, JUN, KLF6, KRT18, LAMC2, LDB1, LGALS3, LTA, MDK, MERTK, MGP, NDP, NOS2, NR2F1, NTRK3, PDGFRA, PIM1, PLAT, RHO, SERPINE1, SLC40A1, SLC4A2, SMAD3, SNAI2, STAT3, TACC2, TDRD7, TGFBR2, THBS2, TIMP1, TNFRSF12A, TYRP1, VCL, VEGFA, YBX2*

All predicted to be decreased.

The predicted activation state is inferred from the bias-corrected z-score, (+ = increased, - = decreased). The bias-corrected z-score is computed based on the proportion of target genes present in the dataset which are directionally regulated as expected according to known associations with functions compiled from the literature.

The *P* value of overlap measures the statistical significance of overlap using Fisher’s exact *t*-test or the Benjamini-Hochberg False Discovery Rate for multiple comparisons (B-H FDR), between genes from the dataset and those known to be associated with a function.

**Table 8:** Upstream regulators predicted by target gene expression in the atretic versus healthy dataset

**Upstream regulator**	**Predicted activation state**	**Bias-corrected z-Score**	***P *** **value of overlap**	**Target molecules in dataset**
TP53	activated	4.272	5.78E-11	*ALB, ANXA1, ANXA2, ARHGEF2, ARPC1B, ATF3, CALD1, CAV1, CFLAR, CLIC4, CNN1, COL18A1, COL1A2, COL3A1, COL4A1, CTGF, CTSH, CYR61, DKK3, DUSP1, EGR1, FBLN2, FERMT2, GADD45A, GLRX, GPX3, GSN, HMGCS1, ID1, ID3, IER3, IFI30, IGF2, IGFBP5, JUN, KRT8, LGALS3, MAP4K1, MMP23B, MPDZ, NDRG1, NOS2, NR2F1, PCDH7, PDE6A, PGFRA, PHLDA1, PIM1, PLAUR, POSTN, RAD51AP1, SAT1, SERPINE1, SGK1, SNAI2, SPP1, STAU1, STK17A, TAGLN2, TGFBR2, THBS2, TOP2A, TP53INP1, VCL, VEGFA*
FOXO4	activated	2.203	5.41E-05	*BCL6, CTGF, GADD45A, HMGCS1, IER3, SERPINE1, SGK1, VEGFA*
CEBPB	activated	2.142	1.59E-07	*ALB, CEBPD, COL1A2, CPT1A, CSN2, CYP19A1, DAB2, DCN, GADD45A, GLIPR2, ID1, IFIT, M3, MGP, MMP11, NDRG4, NOS2, PDGFRA, PDK4, PLAUR, SAT1, SERPINE1, SGK1, SPP1, STAR, TMEM176A, TNFAIP6, TOP1*
RXRA	inhibited	-2.100	3.52E-04	*CEBPD, CPT1A, CYP2C9, FABP5, GCLC, GPT, IER3, LPL, MGP, MMP11, OLR1, PNMT, SAT1, SLC10A2, SPP1, STAR, VEGFA*
HNF1A	inhibited	-2.168	4.07E-01	*ALB, BCL6, C1S, CD55, COL3A1, IHH, SERPINE1, SLC10A2, SLC40A1, SLC4A2, UGT2B4*
MYC	inhibited	-3.197	7.80E-07	*ACTN1, ALB, AQP1, BCL6, CALD1, CAST, CAV1, CD9, CEBPD, CFLAR, CLIC4, COL1A2, COL3A1, COL4A1, CPT1A, CSTB, DSTN, DUSP1, FABP5, FBLN2, GADD45A, GCLC, GTF2F2, HLA-A, ID1, ID3, IER3, KLF6, LUM, NDRG1, PLAUR, PLP1, POLR3D, SGK1, SPP1, TAGLN2, TGFBR2, THBS2, TIMP1, TLN1, TSPO, VEGFA*
MYCN	inhibited	-3.202	8.50E-03	*ARPC1B, CAV1, CCNT1, CITED2, COL18A1, COL4A1, CTGF, DKK3, FGFR2, HLA-A, SDC2, SERPINE1, TAGLN*

The predicted activation state is inferred from the bias-corrected z-score, (+ = activated, - = inhibited). The bias-corrected z-score is computed based on the proportion of target genes present in the dataset which are directionally regulated as expected according to known effects of the regulator on the target compiled from the literature. The *P* value of overlap measures the statistical significance of overlap using Fisher’s exact *t*-test, between genes from the dataset and those known to be acted upon by an upstream regulator.

GO enrichment analyses of this dataset determined associations by the categories of biological process, molecular function and cellular component with atresia in granulosa cells from small follicles (Figure 5 and Additional file 3: Table S2). These analyses revealed that blood vessel development and cell adhesion processes were affected through genes such as *CTGF* and *VEGFA*, and *VNN1* and *PIK3CA*, respectively. The affected products of genes were localised in terms of this analysis to the extracellular matrix and the genes *LAMC2*, *COL1A2*, *COL3A1*and *COL18A1* were found in this group. Other functionally related genes of interest such as *IGF2*, *IGFBP5* and *IGFBP6* indicate that insulin growth factor activity appeared to be modulated during the atretic process.

### Transcriptional processes of atresia

#### Apoptosis

Apoptosis is believed to be the major process of cell death occurring in the antral atretic follicle [7,21]. There was a significant association in our study between atretic follicles and molecules which are influenced by the p53 transcription factor. p53 is an activator of apoptotic pathways in response to cell stress and functions by regulating cell cycle, DNA repair and metabolic genes [22]. Contrary to expectations, many genes which were up regulated in atretic follicles in this study are actually anti-apoptotic or protective against apoptosis such as *CFLAR*[23,24], *PIK3CA*[25,26] and *VNN1*[27]. None of the caspases or Fas genes which are known to be involved with apoptotic pathways were found to be differentially expressed to the same extent as those genes mentioned above. Previous studies in cattle and humans have focused on antral follicles of a larger size [11,14,28,29], than those used in this experiment. It is possible that cell death mechanisms that operate in larger follicles are different from those at an earlier stage, where cells are under different hormonal control, LH in addition to FSH, and in the presence of focimatrix [28]. An alternative theory is that only those granulosa cells in the atretic follicle with elevated expression of anti-apoptotic genes are capable of surviving longer during the process of apoptosis. This theory would fit with the granulosa stem cell model as proposed previously [30]. This model proposes that different types of granulosa cells within the same follicle are derived from stem cells which grow and divide and eventually differentiate into antral, basal and cumulus granulosa cells in the antral follicle. Hence, at any one time not all granulosa cells would be equal and some could be more susceptible to apoptosis than others, as stem cells in muscle [31], neural [32] and bone marrow [33] are (reviewed in [34]).

#### Intercellular junctions

We found increased expression of the claudin tight junction genes *CLDN1*, *CLDN5* and *CLDN11* and the E-cadherin gene, *CDH1*, in atretic follicles. The increase in E-cadherin expression was also verified by immunohistochemistry. Tight junctions act as a critical barrier to the passage of substances between the vasculature and the interior lumen of glands [35] and together with gap junctions help maintain the overall stability of the epithelial layer structure [36]. They are also important for establishing polarity within epithelial cells in general [37]. Properly formed tight junctions have not been observed in the membrana granulosa, though they do exist between Sertoli cells, the analogous cell type in the testis [38]. Gap junctions are present in the granulosa of several species with predominant expression of connexins 43 and 37 (reviewed by [39]). E-cadherin is another protein found at intercellular junctions which is important for cell recognition and adhesion [36], and has been demonstrated in human ovarian follicles [40]. Claudin 1 gene expression has previously been shown to be up regulated during apoptotic insult induced by tamoxifen in some breast tumour cells [41]. Again it is possible that those granulosa cells which have increased expression of these intercellular junction proteins may be able to modulate the effects of apoptotic mechanisms on the cell by stabilising the overall membrana granulosa structure.

#### Inflammation/TGFβ signalling/Tissue remodelling

There have been a number of studies examining TGFβ signalling molecules in follicle development, though not from the aspect of atresia. This is perhaps surprising given that follicular atresia necessitates a cyclic process of tissue remodelling, and the well-known involvement of the TGFβ superfamily in tissue repair. In both IPA and GO enrichment analyses, there were a number of genes found to be associated with inflammatory/TGFβ signalling fibrosis pathways or processes. When we examined the hepatic fibrosis signalling pathway in IPA, with respect to the molecules affected from our dataset, there was increased expression of *TGFBR2* and the downstream SMAD genes and subsequently *COL1A2* and *COL3A1*. The TGFβ receptor has been localised immunohistochemically to the granulosa cells of antral follicles of certain species [42,43], though only in large antral follicles which maybe differentiating as they luteinise. A focus on those genes in our study which are differentially expressed in small follicles, and are expressed at a high level in either the healthy or atretic state, reveals that the inhibin-activin-follistatin axis was most important. *INHA* and *INHBA*, which encode activins and inhibin, and *FST* which produces follistatin, were all down regulated in atretic follicles in our study, which is in agreement with previously published work, as all are required for proper development of the follicle through the antral stage of development [44,45]. *BAMBI* expression in our atretic follicles was increased relative to healthy follicles; the encoded protein is known to bind and inhibit activin and BMP2 (bone morphogenetic protein-2) [46] thus antagonising FSH-induced follicle growth.

Anti-mullerian hormone (AMH) expression was also lower in the atretic follicle. AMH has been shown to delay recruitment of primary follicles to the next stage of growth or possibly atresia [47]. Additionally, AMH has been shown to be up regulated in large dominant bovine follicles versus subdominant and it probably has a survival effect [48]. GDF-9 and BMP-15 are growth factors which have been studied in specific knockouts in mice or mutations in sheep and are known to be important for antral follicle growth (reviewed by [49]). *BMP15* was up regulated 3 fold in small healthy follicles in our study, whereas *GDF9* was relatively unaffected, the proteins encoded by these genes are secreted specifically by the oocyte [50,51] which makes up a small proportion of the total RNA in our samples, so clearly *BMP15* expression was activated but the status of *GDF9* was not apparent. *BMPR2* (bone morphogenetic protein receptor-2) encodes a receptor for GDF-9 and BMP-15 and is therefore also critical for follicle expansion. There was a slight increase in *BMPR2* in the atretic follicle expression in our study, however, this was not significant. Expression of *BMP2* was elevated more than 6 fold in atretic follicles and this member of the BMP family has been implicated in activation of FSH-induced follicle growth and in suppression of luteinisation in human cultured granulosa cells [46]. However, this result was demonstrated in cells obtained from preovulatory follicles and it is unclear whether BMP-2 plays the same role earlier in follicle development.

#### Angiogenesis

*VEGFA* was down regulated in the atretic follicles and *MDK* (midkine; neurite growth-promoting factor 2) and *THBS2* were up regulated in our study. VEGF is a key pro-angiogenic factor and its expression is known to correlate with the size of healthy antral follicles [52] and is weakly expressed in atretic follicles [53]. Thrombospondins-1 and -2 are anti-angiogenic and are expressed highly during tissue remodelling following injury. Thrombospondin-2 in particular appears during the late proliferative phase and is expressed during the remodelling period in conjunction with MMP-2 [54]. Thrombospondin-1 has been shown to promote follicular atresia in rats [55], and together with thrombospondin-2 is expressed inversely with VEGF in a cyclical fashion during folliculogenesis in cows [52]. *MDK* is expressed in many tissues throughout embryonic development, and it has been shown to antagonise VEGF signalling *in vitro* and *in vivo*[56]. Atresia in small antral follicles predictably appears to involve a decrease in blood vessel formation which agrees with previous data.

#### Insulin metabolism

We found increased levels of expression of *IGF2R*, and the binding protein genes *IGFBP5* and *IGFBP6* in atretic follicles with respect to healthy follicles. A number of studies have determined the importance of insulin-like growth factor metabolism at antral stages of follicle development [57-60]. IGF-2 acts similarly to IGF-1 to promote proliferation and growth of granulosa cells, but it is specifically bound by IGF-2 receptor which does not signal by the same pathways as the IGF-1 receptor, but rather acts as a clearance mechanism for IGF-2 [61]. The IGF binding proteins bind IGF-1 and -2, and thus locally regulate their bioavailability in the follicle [62]. IGFBP-5 has previously been shown to increase in atretic follicles in ruminants [63] perhaps due to increased expression and changing levels of degradative enzymes. Therefore it seems that antral atretic follicles have reduced IGF signalling contributing to lower proliferation and decreased metabolism by granulosa cells as has been previously published.

#### Extracellular matrix and matrix proteases

There are several matrix genes which were differentially regulated in our arrays in the atretic follicles including *COL1A2*, *COL3A1*, *COL4A1*, *NID2*, *LAMB1* and *LAMC2*. Immunostaining confirmed that nidogen-2 protein was increased in atretic follicles. Collagens type I and IV were not detectable in the granulosa cells of small follicles at the small antral stage. Previous immunohistochemical studies [64,65] found that the composition of the extracellular matrix did not change, apart from the presence of laminin α2 in atretic but not healthy follicles, and that laminin β1 expression was very weak or nearly absent in small antral atretic follicles [66]. Unfortunately, the laminin α2 gene probe set was not present on the array so this could not be confirmed by the present analysis. It is well known that collagens 1 and 3 are synthesised during the tissue remodelling phase following inflammation [67]. Lee and Dunbar showed an increasing accumulation of laminin β1/γ1 in between granulosa and theca cells in progressively atretic follicles in the pig [68], although a similar study by ourselves in bovine follicles did not indicate differences in laminin β1 in the follicular basement membrane and membrana granulosa [66]. Recently, laminin γ2 was found to be secreted by cultured epithelial cells in response to wounding and may act as a scaffold for cell migration [69]. The expression of these genes in our current study probably represents a step in the eventual regression of the atretic follicle.

*Annexin A2* has been shown to be actively involved in endocytosis and formation of adherens junctions [70]. There is phagocytosis of necrotic cellular debris during atresia and we observed an up regulation of E-cadherin expression in the atretic follicular granulosa cells.

## Conclusions

Small healthy antral follicles, which can undergo a number of growth options, exhibit greater variability in gene expression, particularly in genes associated with cell division and other growth-related functions. It is clear that atresia is associated with transcriptional processes such as the inhibition of blood vessel formation and the differential expression of matrix genes which may signal the surrounding stromal cells to initiate follicular remodelling. Therefore, atresia not only involves changes in expression of genes associated with cell death but it is clearly also an active process not dissimilar to that of wound healing.

## Methods

### Tissues

Ovaries were collected at a local abattoir in South Australia (from non-pregnant *Bos taurus* cows, within 20 min of slaughter and transported to the laboratory in Hank’s balanced-salt solution with Mg^2+^ and Ca^2+^ (HBSS^+/+^; Sigma-Aldrich, Castle Hill, NSW, Australia) on ice. The follicles were dissected from each ovary and the diameter measured. A small piece of the follicle wall, approximately 100 mm^3^, was removed and fixed in 2.5% glutaraldehyde in 0.1 M phosphate buffer (pH 7.25) for subsequent classification of follicle health status. The granulosa cells were removed from the remainder of the follicles by gentle rubbing with a glass Pasteur pipette, previously modified by heat sealing the tip into a rounded smooth surface. The HBSS^-/-^ containing the granulosa cells was centrifuged at 500 ×*g* for 7 min at 4°C, the medium was removed by aspiration and the cells washed twice in phosphate-buffered saline (PBS), pH 7.4. Finally the cells were resuspended in RNAlater (Ambion Life Technologies Australia Pty Ltd., Mulgrave, VIC, Australia), and stored at -20°C for subsequent RNA isolation and microarray analysis. A total of 10 samples of small healthy follicles and 5 small atretic follicles, all < 5 mm and from different animals, were used in this study. Due to limiting RNA, three of the samples from the small healthy follicles were pools of two follicles each from the same animal, whereas the rest were all individual follicles.

### Histological classification of follicles

Following fixation overnight, the follicle wall portions of each follicle were rinsed several times with 0.1 M PBS, pH 7.25, post-fixed in 2% (v/v) aqueous osmium tetroxide for 1 h at 4°C and embedded in epoxy resin as described previously [71]. For light microscopic examination, 0.5 μm thick epoxy sections were cut using a glass knife and a Richert-Jung Ultracut E ultramicrotome (Leica Microsystems Pty Ltd., VIC, Australia), stained with 1% (w/v) aqueous methylene blue and examined using an Olympus BX50 microscope (Olympus Australia Pty Ltd., Mt. Waverly, VIC, Australia). Healthy and atretic follicles were identified as described previously [7,8] wherein healthy follicles had no dead or dying granulosa cells and atretic follicles had substantial numbers of dead and dying granulosa cells. This death in atretic follicles was characterized by a loss of layers closest to the antrum and numerous pyknotic nuclei in the remaining antrally-situated layers [72,73]. The healthy follicle phenotype was sub-classified into two types, rounded or columnar, based on the shape of the basally-situated granulosa cells [15,16]. Additional file 4: Figure S2 shows examples of each of these follicle types.

### RNA isolation

Total RNA was extracted from the granulosa cells using RNeasy mini RNA extraction kits (Qiagen, Hilden, Germany) and RNAqueous-Micro kit™ (Ambion®/Life Technologies Australia Pty Ltd., Mulgrave, VIC, Australia). The concentration of the RNA was determined by spectrophotometric measurement at 260 nm. For each granulosa cell preparation, 5 μg of RNA was treated with DNA-free™ (Ambion Life Technologies). The quality of the RNA was assessed by electrophoresis using an Agilent 2100 Bioanalyser (Agilent Technologies, Santa Clara, CA, USA) and only that with a RNA integrity number exceeding eight was accepted for analysis.

### Real time reverse transcription polymerase chain reaction (real time RT-PCR)

Synthesis of cDNA and real time RT-PCR using plasmid standards were performed as previously [74] and briefly described below. Total RNA (500 ng) was reverse transcribed with SuperScript® III Transcriptase (Life Technologies) using random hexamer primers (Geneworks, Thebarton, SA, Australia) according to the manufacturer’s instructions. Primer Express software (Life Technologies) was used to design primers to the bovine sequences of 18S ribosomal RNA and *CYP17A1* (Table 9). An ABI Prism 7000 Sequence Detection System (Life Technologies) was used for real time reverse transcription RT-PCR detection with SYBR Green (Eppendorf, Hamburg, Germany) and 10 pmoles of forward and reverse primers in a 20 μl reaction. Primer sequences and PCR conditions are shown in Table 9. Plasmid standards were generated by cloning amplified products into pCR2.1-TOPO vector (Life Technologies), then transformed into *E.coli* strain XL1 Blue (Agilent Technologies) and DNA was extracted and purified. These DNA standards were quantitated by absorbance at 260 nm and serially diluted over three logs then amplified together with the diluted sample cDNA in the real time reaction to determine quantities of RNA expressed as fg RNA/ng 18S ribosomal RNA.

**Table 9:** Primers and conditions used for quantitative RT-PCR

**Target**	**Primer sequences, 5′-3′**	**GenBank accession number**	**PCR reaction conditions**
*CYP17A1*	forward accatcagagaagtgctccgaa	NM_174304	2 min 50°C, 10 min 95°C, 40 × cycles of 15 s 95°C and 60 s 60°C
	reverse ccacaacgtctgtgcctttgt		
18S	forward agaaacggctaccacatccaa	DQ2224	2 min 50°C, 10 min 95°C, 40 × cycles of 15 s 95°C and 60 s 60°C
	reverse cctgtattgttatttttcgt		

### Microarray profiling

Following confirmation of the quality of RNA and cDNA synthesis, hybridisations to GeneChip Bovine Genome Arrays (Affymetrix, CA, USA) and scanning were performed according to Affymetrix protocols at the Australian Genome Research Facility (AGRF, Walter & Eliza Hall Institute of Medical Research, Parkville, VIC, Australia) and the Adelaide Microarray Centre (AMC, University of Adelaide, SA, Australia). Between 2 to 5 μg from the small healthy follicles and 250 ng of RNA from small atretic follicles was used per probe preparation with the Affymetrix Genechip 3′ IVT Express kit. Both types of samples followed a similar labelling and hybridisation procedure as detailed below. First-strand cDNA synthesis was performed using a T7-linked oligo-dT primer, followed by second strand synthesis. *In vitro* transcription reactions were performed in batches to generate biotinylated cRNA targets, which were subsequently chemically fragmented at 95°C for 35 min. Ten micrograms of the fragmented, biotinylated cRNA was hybridised at 45°C for 16 h to Affymetrix GeneChip Bovine Genome Arrays, which contain 24,128 probe sets representing over 23,000 transcripts and variants, including 19,000 UniGene clusters. The arrays were then washed and stained with streptavidin-phycoerythrin (final concentration 10 μg/ml). Signal amplification was achieved by using a biotinylated anti-streptavidin antibody. The array was then scanned according to the manufacturer’s instructions. The scanned images were inspected for the presence of any defect on the array.

### Data normalisation and analyses

To minimize discrepancies due to variables such as sample preparation, hybridisation conditions, staining, or array lot, the raw expression data were normalised using RMA background correction (Robust Multi-array Average [75]) with quantile normalisation, log base 2 transformation and mean probe set summarisation with adjustment for GC content which were performed in Partek Genomics Suite Software version 6.5 (Partek Incorporated, St Louis, MO, USA). All samples sent for analysis passed all quality controls. The 15 arrays were analysed as part of a larger set of CEL files (which additionally included samples of granulosa cell RNA from 4 large follicles as discussed elsewhere [18]) uploaded to the Partek GS software program. Before statistical analysis, the data were first subjected to PCA [76] and hierarchical clustering analysis to compare the gene expression patterns of the arrays in terms of our classification. Hierarchical clustering was performed using the Euclidian algorithm for dissimilarity with average linkage. The expression data were analysed by ANOVA using method of moments estimation [77] with post-hoc step-up FDR test for multiple comparisons. The fold change in expression for each gene was based on the non log-transformed values after correction and normalisation. These differentially expressed genes were further annotated and classified based on the Gene Ontology (GO) consortium annotations from the GO *Bos taurus* database (2010/02/24) [78] using GOEAST (Gene Ontology Enrichment Analysis Software Toolkit; [79]). Expression data were also exported to Excel and used to generate size-frequency distributions of the coefficient of variation for each probe set for the two sets of follicles, healthy and atretic. The microarray CEL files, normalised data and experimental information have been deposited in the Gene Expression Omnibus (GEO) database under series record GSE39589.

Pathway analyses of differentially-expressed genes were conducted using IPA software (Redwood City, CA, USA). Network eligible molecules derived from these datasets were overlaid onto a global molecular network developed from information contained in the Ingenuity Knowledge Base. Networks of these molecules were then generated algorithmically based on their connectivity (Ingenuity Systems, 2005). The network score is based on the hypergeometric distribution and is calculated with the right-tailed Fisher’s Exact Test. The score is the negative log of this *P* value. Canonical pathway analysis identified the pathways from the IPA library of canonical pathways that were most significant to the dataset in terms of the ratio of the number of molecules that mapped to the pathway from the dataset and a right-tailed Fisher’s exact *t*-test to determine the probability that the molecules mapped to the pathway by chance alone. We also used IPA Upstream regulator analysis to identify upstream transcriptional regulators. Upstream regulators were predicted using a Fisher’s exact *t*-test to determine the probability that genes from the dataset correspond with targets which are known to be activated or inhibited by those molecules based on current knowledge in the Ingenuity database.

### Immunohistochemistry

Follicles from bovine ovaries were collected and embedded in O.C.T. compound (ProSciTech, Thuringowa, QLD, Australia) and frozen at -80°C. Follicle sections (7 μm) were cut using a CM1800 Leica cryostat (Adeal Pty Ltd., Altona North, Vic, Australia), collected on Superfrost™ glass slides (HD Scientific Supplies, Wetherill Park, Australia), and stored at -20°C until use. Antigen localisation was undertaken on 9 small healthy and 7 small atretic follicles, using an indirect immunofluorescence method as previously described [80]. Frozen follicle sections were dried under vacuum for 5 min, fixed for 5 min and rinsed three times for 5 min in hypertonic PBS (10 mM sodium/potassium phosphate with 0.274 M NaCl, 5 mM KCl, pH 7.2) before treatment with blocking solution [10% normal donkey serum (Sigma-Aldrich) in antibody diluent containing 0.55 M NaCl and 10 mM sodium phosphate (pH 7.1)] for 30 min at room temperature. The sections were incubated with primary antibodies overnight at room temperature. Additional file 5: Table S3 lists the antibodies used for immunofluorescence and relevant fixation conditions. Sections were also treated with the nuclear stain 4′,6′-diamidino-2-phenylindole dihydrochloride (DAPI) solution (Molecular Probes, Eugene, OR, USA) to identify cell nuclei. Coverslips were attached with mounting medium for fluorescence (Dako Corporation, Carpinteria, CA, USA) and photographed with an Olympus BX51TRF microscope with an epifluorescence attachment (Olympus Australia Pty Ltd., Mt Waverley, VIC, Australia) and a Spot RT digital camera (Diagnostic Instruments Inc Pty Ltd., Victora Park, WA, Australia). Negative controls included no primary antisera and non-immune mouse, rabbit or rat serum. No staining of ovarian structures was observed with these controls.

## Abbreviations

FDR: Benjamini-Hochberg False Discovery Rate; FSH: Follicle-stimulating hormone; GO: Gene ontology; GOEAST: Gene Ontology Enrichment Analysis Software Toolkit; GEO: Gene Expression Omnibus; HBBS: Hank’s balanced-salt solution; IPA: Ingenuity Pathway Analysis; LH: Luteinising hormone; PCA: Principal Component Analysis; RMA: Robust multi-array average; RT-PCR: Reverse transcription polymerase chain reaction; TGFβ: Transforming growth factor beta; TNFα: Tumour necrosis factor alpha.

## Competing interests

No conflicts of interest, financial or otherwise, are declared by the authors.

## Authors’ contributions

Conceived and designed the experiments: HFI-R and RJR. Performed the experiments: NH, KH HFI-R, MLH and SEM. Analysed the data: NH, KH and RJR. Contributed reagents/materials/analysis tools: NH, KH and RJR. Wrote the paper: NH, KH, HFI-R and RJR. All authors read and approved the final manuscript.

## Supplementary Material

Additional file 1: Table S1 Probe sets which were three fold or more up regulated in granulosa cells of small atretic follicles with respect to small healthy follicles by ANOVA in Partek, with *P <* 0.0*5* (n = 1595), in alphabetical order. Probe sets which did not have gene assignations are placed at the end of the list. The *P* value for multiple corrections was determined by the step-up FDR method. Click here for file

Additional file 2: Figure S1 The canonical TGFβ signalling pathway as presented in IPA showing genes which were 4 fold differentially expressed with a FDR *P <* 0.005 between atretic and healthy follicles from our dataset. Genes which were up regulated in atretic follicles are indicated in red, and those which were down regulated are green, with the degree of fold difference commensurate with the colour intensity. Click here for file

Additional file 3: Table S2 Genes which were differentially expressed (4 fold, *P <* 0.005) in atretic follicles with respect to healthy follicles and associated with significant GO terms after enrichment analysis by GOEAST. Click here for file

Additional file 4: Figure S2 Histological classification of small antral follicles. Methylene blue stained semi-thin sections of (A) healthy rounded, (B) healthy columnar and (C) atretic small antral follicles. Bar = 50 μm. Click here for file

Additional file 5: Table S3 Primary antibodies, secondary antibodies, labelling and fixation conditions used for immunohistochemistry for each antigen. Secondary antibodies used were either biotin-SP-conjugated AffiniPure donkey-anti-mouse IgG (Cat. no. 715-066-151), followed by Cy3-conjugated streptavidin (SA-Cy3, Cat. # 016-160-084) or fluorescein/DTAF-conjugated streptavidin (SA-DTAF, Cat. # 016-010-084), or Cy3-conjugated AffiniPure donkey-anti-rabbit IgG (Cat. # 711-166-152) or anti-mouse (Cat. # 715-166-151) or FITC-conjugated AffiniPure donkey-anti-rat IgG (Cat. # 712-096-153). All secondary antibodies and conjugated streptavidins were purchased from Jackson ImmunoResearch Laboratories Inc. (West Grove, PA, USA) and used at 1:100 dilutions. Click here for file
