# Supplementary material for: Transcriptome profiling of granulosa cells from bovine ovarian follicles during atresia
Source: BMC Genomics. 2014 Jan 18;15:40. doi: 10.1186/1471-2164-15-40 (PMC3898078; doi:10.1186/1471-2164-15-40)
Supplement: Additional file 1: Table S1 — Probe sets which were three fold or more up regulated in granulosa cells of small atretic follicles with respect to small healthy follicles by ANOVA in Partek, with P < 0.05 (n = 1595), in alphabetical order. Probe sets which did not have gene assignations are placed at the end of the list. The P value for multiple corrections was determined by the step-up FDR method. [file 1471-2164-15-40-S1.pdf]

Probeset ID	Gene Symbol	Entrez Gene Name	Fold-Change	*P*-value
Bt.23514.1.S1_at	A2M	alpha-2-macroglobulin	8.120	3.04E-08
Bt.15958.2.A1_a_at	ABCB1	ATP-binding cassette, sub-family B (MDR/TAP), member 1	3.843	3.13E-06
Bt.16637.1.A1_at	ABCD1	ATP-binding cassette, sub-family D (ALD), member 1	-4.158	1.27E-06
Bt.16041.2.S1_at	ABLIM1	actin binding LIM protein 1	3.502	1.09E-02
Bt.3255.1.A1_at	ABP1	amiloride binding protein 1 (amine oxidase (copper-containing))	-3.277	1.90E-06
Bt.26530.2.S1_at	ACAD10	acyl-CoA dehydrogenase family, member 10	-3.162	1.56E-07
Bt.28278.1.S1_at	ACE2	angiotensin I converting enzyme (peptidyl-dipeptidase A) 2	-3.301	5.15E-05
Bt.4604.1.S1_a_at	ACSM1	acyl-CoA synthetase medium-chain family member 1	-3.149	4.06E-03
Bt.17231.1.A1_at	ACSM2A	acyl-CoA synthetase medium-chain family member 2A	-3.142	1.29E-04
Bt.12825.1.S1_at	ACTA2	actin, alpha 2, smooth muscle, aorta	4.280	2.55E-03
Bt.7700.1.S1_at	ACTN1	actinin, alpha 1	4.055	4.11E-04

Bt.16020.1.A2_at	ACVR1	activin A receptor, type I	3.106	3.92E-06
Bt.12010.1.S1_at	ADAMTS1	ADAM metallopeptidase with thrombospondin type 1 motif, 1	6.142	2.51E-05
Bt.2047.1.S1_at	ADM	adrenomedullin	3.856	8.33E-04
Bt.374.1.S1_at	ADRA1A	adrenergic, alpha-1A-, receptor	-3.685	4.49E-06
Bt.29129.1.S1_at	AGR2	anterior gradient homolog 2 (Xenopus laevis)	-5.753	2.09E-08
Bt.15735.1.S1_at	AGRN	agrin	4.084	4.02E-05
Bt.23331.1.S1_at	AHNAK	AHNAK nucleoprotein	3.559	3.07E-03
Bt.13046.1.S1_at	AHR	aryl hydrocarbon receptor	-3.161	5.16E-08
Bt.10983.1.S1_at	AIF1L	allograft inflammatory factor 1-like	4.376	5.96E-06
Bt.4892.1.A1_at	AKAP8L	A kinase (PRKA) anchor protein 8-like	4.144	5.97E-05
Bt.5130.1.A1_a_at	ALB	albumin	-5.732	5.43E-08
Bt.4732.1.S1_at	ALDH1A1	aldehyde dehydrogenase 1 family, member A1	3.266	8.40E-03

Bt.3284.2.A1_at	ALG3	asparagine-linked glycosylation 3, alpha-1,3-mannosyltransferase homolog	-7.001	1.47E-10
Bt.17638.1.A1_at	ALG5	Asparagine-linked glycosylation 5, dolichyl-phosphate beta-glucosyltransferase	-3.193	9.53E-08
Bt.6653.3.S1_at	ALKBH4	alkB, alkylation repair homolog 4 (E. coli)	3.435	1.49E-06
Bt.18189.1.A1_at	ALOX12B	arachidonate 12-lipoxygenase, 12R type	-3.815	1.98E-08
Bt.28016.1.S1_s_at	AMELX ///AMELY	amelogenin, X-linked /// amelogenin, Y-linked	-3.294	3.83E-09
Bt.7050.1.S1_at	AMH	anti-Mullerian hormone	-4.233	2.11E-03
Bt.12294.2.A1_at	AMIGO2	Adhesion molecule with Ig-like domain 2	4.461	2.77E-05
Bt.833.2.S1_at	ANAPC5	anaphase promoting complex subunit 5	-4.444	7.30E-05
Bt.4816.1.S1_at	ANGPTL4	angiopoietin-like 4	3.361	1.91E-05
Bt.26461.1.A1_at	ANKH	ankylosis, progressive homolog (mouse)	3.597	3.97E-05
Bt.15836.1.S1_at	ANKRD1	ankyrin repeat domain 1 (cardiac muscle)	48.941	9.66E-07
Bt.15836.2.S1_at	ANKRD1	ankyrin repeat domain 1 (cardiac muscle)	30.286	3.92E-07

Bt.14043.2.S1_at	ANKRD10	ankyrin repeat domain 10	3.017	8.49E-04
Bt.1584.1.S1_at	ANKRD11	ankyrin repeat domain 11	3.155	1.30E-03
Bt.17978.1.A1_at	ANKRD17	ankyrin repeat domain 17	-3.326	3.46E-07
Bt.28853.1.A1_at	ANKRD43	Ankyrin repeat domain 43	-3.266	5.45E-03
Bt.23438.1.S1_at	ANTXR2	anthrax toxin receptor 2	3.687	1.98E-05
Bt.16032.1.S1_at	ANXA1	annexin A1	10.895	1.83E-04
Bt.4314.1.S1_at	ANXA2	annexin A2	4.658	6.90E-05
Bt.6775.1.S1_at	ANXA3	annexin A3	9.273	2.58E-05
Bt.18962.1.A1_at	AOAH	acyloxyacyl hydrolase (neutrophil)	-8.796	1.88E-08
Bt.21853.1.S1_at	AP1S2	adaptor-related protein complex 1, sigma 2 subunit	3.127	5.15E-05
Bt.25516.1.A1_at	AP4B1	adaptor-related protein complex 4, beta 1 subunit	-3.232	1.06E-07
Bt.9211.1.S1_at	APBB1IP	amyloid beta (A4) precursor protein-binding, family B, member 1 interacting protein	4.568	1.13E-04

Bt.19756.1.S1_at	APOB	apolipoprotein B	-4.972	1.58E-07
Bt.13975.1.S1_a_at	APOD	apolipoprotein D	37.500	6.08E-07
Bt.13975.2.A1_at	APOD	Apolipoprotein D	20.737	7.01E-06
Bt.12718.1.A1_at	APOD	Apolipoprotein D	5.987	1.02E-02
Bt.29460.1.A1_at	AQP1	aquaporin 1 (Colton blood group)	-7.007	2.46E-07
Bt.26666.1.S1_at	ARF5	ADP-ribosylation factor 5	3.106	1.48E-07
Bt.20269.1.S1_at	ARF6	ADP-ribosylation factor 6	-3.275	6.38E-07
Bt.20527.1.S1_at	ARHGAP32	Rho GTPase activating protein 32	3.504	6.49E-07
Bt.24442.1.A1_at	ARHGEF11	Rho guanine nucleotide exchange factor (GEF) 11	9.896	3.66E-04
Bt.17672.1.S1_at	ARHGEF2	Rho/Rac guanine nucleotide exchange factor (GEF) 2	4.031	5.45E-07
Bt.3236.1.A1_at	ARHGEF3	Rho guanine nucleotide exchange factor (GEF) 3	7.125	7.70E-06
Bt.19965.1.S1_at	ARMCX3	armadillo repeat containing, X-linked 3	3.182	2.45E-05
Bt.23192.1.S1_at	ARPC1B	actin related protein 2/3 complex, subunit 1B, 41kDa	5.475	1.50E-06

Bt.26878.1.S1_at	ASB12	ankyrin repeat and SOCS box-containing 12	-3.300	1.63E-08
Bt.3227.1.S1_at	ASCC2	activating signal cointegrator 1 complex subunit 2	3.528	9.63E-06
Bt.23658.1.A1_at	ASF1B	ASF1 anti-silencing function 1 homolog B (S. cerevisiae)	-3.684	1.58E-03
Bt.22257.1.S1_at	ASGR1	asialoglycoprotein receptor 1	-3.099	1.41E-08
Bt.25685.1.A1_at	ASPHD1	aspartate beta-hydroxylase domain containing 1	-3.303	3.83E-07
Bt.19819.1.S1_at	ASPM	asp (abnormal spindle) homolog, microcephaly associated (Drosophila)	-3.207	1.49E-02
Bt.18533.1.S1_at	ATF3	activating transcription factor 3	9.676	5.45E-07
Bt.9785.1.S1_at	ATG14	ATG14 autophagy related 14 homolog (S. cerevisiae)	3.066	9.40E-06
Bt.28752.1.A1_at	ATP10A	ATPase, class V, type 10A	-6.090	4.79E-07
Bt.889.1.S1_at	ATP13A2	ATPase type 13A2	-3.770	1.43E-07
Bt.12785.1.S1_at	ATP2B4	ATPase, Ca++ transporting, plasma membrane 4	-4.382	1.76E-08
Bt.28365.1.A1_at	ATP4A	ATPase, H+/K+ exchanging, alpha polypeptide	-3.196	1.08E-07

Bt.23985.1.A1_at	ATRX	alpha thalassemia/mental retardation syndrome X-linked	8.428	7.58E-08
Bt.7331.1.S2_at	AURKB	aurora kinase B	-3.034	8.07E-03
Bt.7537.1.S1_at	AUTS2	autism susceptibility candidate 2	3.397	2.80E-06
Bt.6865.1.S1_at	AXL	AXL receptor tyrosine kinase	25.001	5.11E-06
Bt.1272.1.S1_at	B3GNT3	UDP-GlcNAc:betaGal beta-1,3-N-acetylglucosaminyltransferase 3	-3.085	1.85E-04
Bt.13147.1.S1_at	B4GALT5	UDP-Gal:betaGlcNAc beta 1,4- galactosyltransferase, polypeptide 5	3.420	4.39E-06
Bt.1960.2.S1_a_at	B9D2	B9 protein domain 2	3.020	4.74E-06
Bt.5177.1.S1_at	BAMBI	BMP and activin membrane-bound inhibitor homolog (Xenopus laevis)	7.606	1.10E-05
Bt.4898.1.S1_at	BASP1	brain abundant, membrane attached signal protein 1	3.271	1.81E-02
Bt.14017.1.S1_at	BAT2L2	HLA-B associated transcript 2-like 2	3.240	1.25E-07
Bt.16661.1.A1_at	BAZ1A	bromodomain adjacent to zinc finger domain, 1A	3.043	8.05E-04
Bt.21759.2.A1_at	BBOX1	Butyrobetaine (gamma), 2-oxoglutarate dioxygenase	-3.485	2.57E-06

		(gamma-butyrobetaine hydroxylase) 1		
Bt.15826.1.A1_at	BCAR1	breast cancer anti-estrogen resistance 1	3.123	1.21E-08
Bt.17278.3.S1_at	BCAT1	branched chain amino-acid transaminase 1, cytosolic	-3.500	1.21E-05
Bt.7670.1.A1_at	BCL6	B-cell CLL/lymphoma 6	8.556	1.75E-07
Bt.17758.1.S1_at	BCOR	BCL6 co-repressor	-3.502	8.28E-05
Bt.26266.1.A1_at	BCORL1	BCL6 corepressor-like 1	-3.218	1.15E-07
Bt.550.1.S1_at	BDA20	major allergen BDA20	-3.963	1.16E-08
Bt.2353.1.S1_at	BIRC5	baculoviral IAP repeat-containing 5	-3.399	1.06E-02
Bt.22954.1.S1_at	BMP15	bone morphogenetic protein 15	-3.808	6.78E-08
Bt.29956.1.A1_at	BMP2	bone morphogenetic protein 2	6.572	2.71E-04
Bt.27778.1.A1_at	BMPER	BMP binding endothelial regulator	3.663	9.54E-04
Bt.13357.1.S1_at	BOD1L	biorientation of chromosomes in cell division 1-like	3.062	7.44E-06
Bt.29817.1.A1_x_at	BOLA	MHC class I heavy chain	-4.659	2.20E-10

Bt.29817.1.A1_at	BOLA	MHC class I heavy chain	-6.229	1.73E-11
Bt.4751.2.S1_a_at	BOLA-DQA2	major histocompatibility complex, class II, DQ alpha 2	-3.293	8.79E-07
Bt.3805.1.S1_at	BOLA-N /// JSP.1	MHC class I antigen /// MHC Class I JSP.1	-8.255	2.46E-04
Bt.12872.1.S1_at	BTBD3	BTB (POZ) domain containing 3	6.111	3.49E-07
Bt.24946.1.A1_at	BTN3A3	butyrophilin, subfamily 3, member A3	-3.550	2.12E-05
Bt.21513.1.A1_at	BUB1	budding uninhibited by benzimidazoles 1 homolog (yeast)	-3.943	1.06E-02
Bt.25661.1.A1_at	BUB1B	budding uninhibited by benzimidazoles 1 homolog beta (yeast)	-4.235	5.31E-03
Bt.24704.1.S1_at	C11HXORF26	chromosome X open reading frame 26 ortholog	3.900	8.24E-06
Bt.2806.1.S1_at	C13H20ORF12	chromosome 20 open reading frame 12 ortholog	3.493	6.79E-03
Bt.26881.1.S1_at	C16H1orf170	Chromosome 1 open reading frame 170 ortholog	-4.189	3.88E-07
Bt.2391.2.S1_a_at	C16H1ORF55	chromosome 1 open reading frame 55 ortholog	6.335	8.99E-07
Bt.22629.1.A1_at	C1H21orf62	chromosome 21 open reading frame 62 ortholog	-4.215	4.15E-08

Bt.2823.3.S1_a_at	C1orf35	chromosome 1 open reading frame 35	4.153	1.00E-06
Bt.25849.1.S1_at	C1QTNF6	C1q and tumor necrosis factor related protein 6	4.120	7.06E-05
Bt.1491.1.S1_at	C1S	complement component 1, s subcomponent	5.933	4.18E-05
Bt.21809.1.S1_at	C22H3ORF19	chromosome 3 open reading frame 19 ortholog	3.998	6.24E-08
Bt.5192.2.S1_at	C23H6ORF47	chromosome 6 open reading frame 47 ortholog	-3.573	3.30E-08
Bt.3016.1.A1_at	C26H10ORF6	Chromosome 10 open reading frame 6 ortholog	-3.003	5.60E-07
Bt.5598.1.S1_at	C27H8orf4	chromosome 8 open reading frame 4 ortholog	37.183	9.94E-07
Bt.14219.1.S1_at	C28H10ORF10	chromosome 10 open reading frame 10 ortholog	6.997	2.33E-04
Bt.9256.2.S1_a_at	C3H1ORF212	chromosome 1 open reading frame 212 ortholog	3.125	1.11E-05
Bt.17116.1.A1_at	C6	complement component 6	-4.431	8.57E-08
Bt.5164.1.S1_at	CA14	carbonic anhydrase XIV	-6.504	4.65E-04
Bt.22854.1.S1_at	CA2	carbonic anhydrase II	4.765	6.64E-04
Bt.26040.1.A1_at	CA5B	carbonic anhydrase VB, mitochondrial	-3.180	6.46E-07

Bt.13069.2.S1_at	CACNA1D	calcium channel, voltage-dependent, L type, alpha 1D subunit	-3.069	5.33E-06
Bt.5478.1.S1_at	CACNB3	calcium channel, voltage-dependent, beta 3 subunit	4.040	4.72E-06
Bt.3806.1.A1_at	CADM1	cell adhesion molecule 1	3.292	2.09E-04
Bt.23326.2.A1_at	CALD1	Caldesmon 1	11.621	3.68E-07
Bt.5499.1.S1_at	CAPG	capping protein (actin filament), gelsolin-like	11.138	7.55E-06
Bt.20860.3.A1_at	CAPSL	calcyphosine-like	-3.359	2.39E-05
Bt.29902.1.S1_at	CARD10	caspase recruitment domain family, member 10	3.050	1.96E-05
Bt.27531.1.A1_at	CASC5	cancer susceptibility candidate 5	-4.057	2.32E-03
Bt.5121.1.S1_at	CASR	calcium-sensing receptor	-3.018	1.12E-06
Bt.4738.1.S2_at	CAST	calpastatin	6.737	5.09E-06
Bt.4738.1.S1_at	CAST	calpastatin	3.437	4.16E-05
Bt.310.1.S1_at	CATHL1	cathelicidin 1	-4.737	6.25E-07

Bt.3478.1.A1_s_at	CAV1	caveolin 1, caveolae protein, 22kDa	4.594	1.98E-06
Bt.16056.1.A1_at	CBLB	Cas-Br-M (murine) ecotropic retroviral transforming sequence b	3.585	6.11E-06
Bt.21034.1.A1_at	CC2D1B	Coiled-coil and C2 domain containing 1B	-3.154	5.05E-06
Bt.4981.1.S1_at	CCBL1	cysteine conjugate-beta lyase, cytoplasmic	-4.248	1.04E-03
Bt.25465.1.A1_at	CCDC132	Coiled-coil domain containing 132	-3.184	1.62E-07
Bt.17307.1.A1_at	CCDC159	coiled-coil domain containing 159	-4.265	3.25E-07
Bt.27552.2.S1_at	CCDC21	coiled-coil domain containing 21	-4.507	1.27E-08
Bt.3946.2.S1_at	CCDC3	coiled-coil domain containing 3	-5.847	5.13E-06
Bt.3946.3.S1_at	CCDC3	coiled-coil domain containing 3	-6.305	2.55E-06
Bt.13645.2.S1_at	CCDC43	Coiled-coil domain containing 43	-3.281	7.17E-08
Bt.7788.1.S1_at	CCDC80	coiled-coil domain containing 80	3.008	2.81E-03
Bt.6516.1.A1_at	CCDC85B	coiled-coil domain containing 85B	4.092	1.60E-05

Bt.29176.1.S1_at	CCDC97	coiled-coil domain containing 97	-3.295	9.61E-07
Bt.24326.1.S1_at	CCL11	chemokine (C-C motif) ligand 11	-3.415	6.35E-07
Bt.9577.1.S1_a_at	CCL25	chemokine (C-C motif) ligand 25	-3.378	1.77E-04
Bt.16745.1.A1_at	CCL28	Chemokine (C-C motif) ligand 28	-3.370	2.83E-06
Bt.15980.1.A1_at	CCNB1	cyclin B1	-3.555	2.69E-02
Bt.29872.1.S1_at	CCNT1	cyclin T1	-4.543	2.61E-07
Bt.28967.2.A1_at	CCR3	chemokine (C-C motif) receptor 3	-3.186	2.50E-07
Bt.5861.1.S1_at	CD200	CD200 molecule	6.957	1.14E-03
Bt.2577.1.S1_at	CD24	CD24 molecule	17.131	3.75E-08
Bt.3484.1.S1_at	CD302	CD302 molecule	3.661	1.50E-06
Bt.26763.1.A1_at	CD33	CD33 antigen-like	-3.527	1.19E-05
Bt.4497.1.S1_at	CD5	CD5 molecule	-3.578	2.48E-07
Bt.28393.1.S1_at	CD55	CD55 molecule, decay accelerating factor for	6.153	1.41E-07

		complement (Cromer blood group)		
Bt.21980.1.S1_at	CD72	CD72 molecule	-3.319	1.52E-05
Bt.3841.1.S1_at	CD83	CD83 molecule	3.697	9.43E-04
Bt.20521.1.A1_at	CD84	CD84 molecule	-3.769	2.97E-07
Bt.2573.1.S1_at	CD9	CD9 molecule	7.079	3.81E-06
Bt.20940.1.S1_at	CD96	CD96 molecule	-3.162	9.56E-08
Bt.7931.1.S1_at	CD99	CD99 molecule	3.739	4.33E-03
Bt.639.1.S1_at	CDC20	cell division cycle 20 homolog (S. cerevisiae)	-3.759	1.13E-02
Bt.9516.1.S1_at	CDC37L1	cell division cycle 37 homolog (S. cerevisiae)-like 1	5.937	7.83E-07
Bt.24218.1.S1_at	CDC6	cell division cycle 6 homolog (S. cerevisiae)	-3.660	8.83E-04
Bt.29462.1.S1_at	CDCA2	cell division cycle associated 2	-3.324	1.93E-02
Bt.10696.1.S1_at	CDCA3	cell division cycle associated 3	-3.258	1.15E-02
Bt.27319.1.A1_at	CDCA8	cell division cycle associated 8	-3.738	4.58E-03

Bt.27319.2.S1_at	CDCA8	cell division cycle associated 8	-4.610	2.30E-06
Bt.11241.1.S1_at	CDH1	cadherin 1, type 1, E-cadherin (epithelial)	4.290	3.14E-03
Bt.28734.1.S1_at	CDH26	cadherin 26	-3.475	1.45E-06
Bt.4804.2.A1_at	CDKN1C	cyclin-dependent kinase inhibitor 1C (p57, Kip2)	40.184	2.72E-08
Bt.6406.1.S3_at	CEBPD	CCAAT/enhancer binding protein (C/EBP), delta	5.960	6.22E-05
Bt.6406.1.S2_at	CEBPD	CCAAT/enhancer binding protein (C/EBP), delta	5.380	2.88E-05
Bt.21523.1.S1_at	CENP-A	centromere protein-A	-6.208	7.71E-04
Bt.29850.1.S1_at	CENP-A	centromere protein-A	-3.112	1.59E-04
Bt.24844.1.S1_at	CENPE	centromere protein E, 312kDa	-4.461	1.12E-03
Bt.12328.1.S1_at	CENPF	centromere protein F, 350/400kDa (mitosin)	-3.312	2.76E-02
Bt.28797.1.A1_at	CENPN	centromere protein N	-3.082	1.71E-02
Bt.28797.2.S1_at	CENPN	centromere protein N	-3.530	4.15E-03
Bt.4336.1.S1_at	CFD	complement factor D (adipsin)	3.761	2.45E-08

Bt.989.2.S1_at	CFDP2	craniofacial development protein 2	-3.098	1.65E-05
Bt.13556.1.S1_a_at	CFH	complement factor H	4.430	2.66E-05
Bt.18643.2.S1_at	CFLAR	CASP8 and FADD-like apoptosis regulator	6.012	3.35E-06
Bt.28669.1.A1_at	CHAC1	ChaC, cation transport regulator homolog 1 (E. coli)	-3.509	3.43E-06
Bt.14081.2.S1_at	CHMP5	chromatin modifying protein 5	3.735	2.25E-05
Bt.25582.1.A1_at	CHRM4	cholinergic receptor, muscarinic 4	-3.180	3.52E-06
Bt.16831.1.A1_at	CHST2	carbohydrate (N-acetylglucosamine-6-O) sulfotransferase 2	3.974	4.26E-07
Bt.21607.1.S1_at	CITED2	Cbp/p300-interacting transactivator, with Glu/Asp-rich carboxy-terminal domain,2	5.581	1.94E-05
Bt.10007.1.A1_at	CKAP2	cytoskeleton associated protein 2	-3.086	5.62E-03
Bt.5578.1.S1_at	CKAP2L	cytoskeleton associated protein 2-like	-4.730	4.99E-04
Bt.3885.4.S1_x_at	CLCA3P	chloride channel accessory 3 (pseudogene)	3.922	3.11E-04
Bt.24343.2.S1_at	CLDN1	claudin 1	10.226	8.57E-05

Bt.24343.1.A1_at	CLDN1	claudin 1	5.274	5.20E-03
Bt.4817.1.A1_at	CLDN11	claudin 11	4.873	2.80E-04
Bt.483.1.S1_at	CLDN16	claudin 16	-3.501	3.29E-07
Bt.21028.1.S1_at	CLDN5	claudin 5	6.344	4.34E-04
Bt.24617.1.A1_at	CLDN6	claudin 6	-3.772	3.15E-08
Bt.16271.1.S1_at	CLEC4E	C-type lectin domain family 4, member E	-3.013	2.14E-08
Bt.2563.1.S1_at	CLIC4	chloride intracellular channel 4	4.483	2.81E-08
Bt.12504.1.S1_at	CLU	clusterin	3.641	2.73E-04
Bt.3203.1.S1_at	CMBL	carboxymethylenebutenolidase homolog (Pseudomonas)	-4.205	4.57E-04
Bt.13240.1.S1_at	CMTM3	CKLF-like MARVEL transmembrane domain containing 3	3.537	2.42E-06
Bt.6630.1.S1_at	CNN1	calponin 1, basic, smooth muscle	4.411	9.39E-07
Bt.27324.1.A1_at	CNNM2	cyclin M2	-4.188	5.97E-07

Bt.73.1.S1_at	COL10A1	collagen, type X, alpha 1	-3.512	5.14E-07
Bt.10442.2.S1_a_at	COL12A1	collagen, type XII, alpha 1	3.351	1.77E-03
Bt.24962.1.A1_at	COL18A1	collagen, type XVIII, alpha 1	4.026	6.17E-05
Bt.11942.1.S1_at	COL18A1	collagen, type XVIII, alpha 1	3.637	7.52E-04
Bt.8124.1.S1_at	COL1A2	collagen, type I, alpha 2	5.279	1.64E-03
Bt.23318.1.S1_at	COL3A1	collagen, type III, alpha 1	5.476	1.82E-03
Bt.23318.1.S2_at	COL3A1	collagen, type III, alpha 1	4.569	4.26E-03
Bt.12912.1.S2_at	COL4A1	collagen, type IV, alpha 1	4.518	4.27E-06
Bt.16495.2.A1_at	COL4A5	collagen, type IV, alpha 5	3.539	2.42E-05
Bt.16495.1.A1_at	COL4A5	collagen, type IV, alpha 5	3.510	3.97E-04
Bt.23508.1.A1_at	COL6A1	collagen, type VI, alpha 1	3.165	4.62E-04
Bt.28134.1.S1_at	COL6A6	Collagen, type VI, alpha 6	-3.735	2.89E-06
Bt.9576.2.S1_a_at	COMMD4	COMM domain containing 4	3.364	2.03E-07

Bt.351.1.S1_at	CORO1A	coronin, actin binding protein, 1A	3.209	1.43E-05
Bt.28370.1.A1_at	CPB1	carboxypeptidase B1 (tissue)	-3.254	1.83E-07
Bt.14204.1.A1_at	CPEB2	cytoplasmic polyadenylation element binding protein 2	3.597	3.16E-05
Bt.20407.2.A1_at	CPEB4	cytoplasmic polyadenylation element binding protein 4	3.150	1.06E-06
Bt.14726.1.A1_at	CPS1	carbamoyl-phosphate synthase 1, mitochondrial	-3.030	4.78E-06
Bt.9289.1.S1_at	CPT1A	Carnitine palmitoyltransferase 1A (liver)	4.462	3.90E-04
Bt.17317.1.S1_at	CPXM2	carboxypeptidase X (M14 family), member 2	-5.194	3.34E-07
Bt.1151.1.S1_at	CRABP2	cellular retinoic acid binding protein 2	3.746	4.33E-03
Bt.1927.1.S1_at	CRISPLD2	cysteine-rich secretory protein LCCL domain containing 2	-5.208	1.62E-03
Bt.23701.1.A1_at	CRP	C-reactive protein, pentraxin-related	-3.122	1.97E-07
Bt.222.1.S1_at	CRYAB	crystallin, alpha B	5.106	2.37E-03
Bt.21180.1.S1_at	CSDC2	cold shock domain containing C2, RNA binding	3.064	8.15E-04

Bt.5381.2.S1_x_at	CSN2	casein beta	-17.737	8.15E-11
Bt.583.1.S1_a_at	CSN3	casein kappa	-3.375	9.65E-07
Bt.28499.1.S1_at	CSNK1E	casein kinase 1, epsilon	3.287	2.58E-05
Bt.20041.1.S1_at	CSNK1G2	casein kinase 1, gamma 2	4.234	5.78E-05
Bt.1.1.S1_at	CSNK2A1	casein kinase 2, alpha 1 polypeptide	3.676	3.70E-06
Bt.20215.1.S1_at	CSRNP1	cysteine-serine-rich nuclear protein 1	3.317	7.49E-08
Bt.24354.1.S1_at	CSTB	cystatin B (stefin B)	9.280	5.14E-05
Bt.1986.1.S1_at	CSTB	cystatin B (stefin B)	6.657	4.25E-06
Bt.5240.1.S1_at	CTGF	connective tissue growth factor	23.359	1.15E-05
Bt.2943.1.A1_at	CTR9	Ctr9, Paf1/RNA polymerase II complex component, homolog (S. cerevisiae)	5.542	5.54E-08
Bt.393.1.S1_at	CTSB	cathepsin B	3.244	8.21E-07
Bt.1031.1.S1_at	CTSH	cathepsin H	4.722	2.19E-04

Bt.23218.1.S1_at	CTSK	cathepsin K	3.246	1.80E-03
Bt.7938.1.S1_at	CTSS	cathepsin S	4.371	1.46E-04
Bt.4902.1.S1_at	CTSZ	cathepsin Z	3.456	2.90E-05
Bt.17384.2.S1_at	CTTNBP2NL	CTTNBP2 N-terminal like	3.666	3.71E-08
Bt.29097.1.A1_at	CUL7	cullin 7	-4.345	6.29E-07
Bt.25457.1.A1_at	CWC22	CWC22 spliceosome-associated protein homolog (S. cerevisiae)	3.236	3.10E-06
Bt.20635.1.S1_at	CXCL17	chemokine (C-X-C motif) ligand 17	-3.716	1.04E-07
Bt.81.1.S1_at	CYLC1	cylicin, basic protein of sperm head cytoskeleton 1	-4.271	3.42E-07
Bt.27817.1.S1_at	CYLD	cylindromatosis (turban tumor syndrome)	3.657	1.69E-07
Bt.4447.2.S1_a_at	CYP19A1	cytochrome P450, family 19, subfamily A, polypeptide 1	-3.374	5.02E-05
Bt.4447.1.S1_at	CYP19A1	cytochrome P450, family 19, subfamily A, polypeptide 1	-19.776	2.12E-05
Bt.1964.1.S1_at	CYP21A2	cytochrome P450, family 21, subfamily A, polypeptide 2	-3.559	5.43E-08

Bt.27036.1.S1_at	CYP4F2	cytochrome P450, family 4, subfamily F, polypeptide 2	-4.182	6.46E-08
Bt.22000.1.A1_at	CYR61	cysteine-rich, angiogenic inducer, 61	14.062	1.18E-05
Bt.3090.1.S1_at	CYTH3	Cytohesin 3	3.401	2.93E-05
Bt.3814.1.S1_at	DAB2	disabled homolog 2, mitogen-responsive phosphoprotein (Drosophila)	4.258	4.68E-04
Bt.24335.1.S1_at	DAPP1	dual adaptor of phosphotyrosine and 3-phosphoinositides	5.799	1.40E-06
Bt.25949.1.A1_at	DBC1	deleted in bladder cancer 1	-3.181	2.06E-06
Bt.4474.2.S1_a_at	DBNDD2	dysbindin (dystrobrevin binding protein 1) domain containing 2	9.183	2.66E-08
Bt.4474.1.S1_at	DBNDD2	dysbindin (dystrobrevin binding protein 1) domain containing 2	3.047	3.54E-06
Bt.18489.1.A1_at	DBT	dihydrolipoamide branched chain transacylase E2	-3.876	1.80E-05
Bt.14572.1.A1_at	DCLK1	doublecortin-like kinase 1	19.519	1.40E-05
Bt.19199.1.A1_at	DCLK1	Doublecortin-like kinase 1	3.789	4.64E-05

Bt.23178.1.S1_at	DCN	decorin	11.086	1.20E-05
Bt.23178.1.S2_at	DCN	decorin	6.020	4.91E-05
Bt.16757.2.A1_at	DCP1A	DCP1 decapping enzyme homolog A (S. cerevisiae)	-3.595	2.78E-09
Bt.22953.1.S1_at	DCT	dopachrome tautomerase (dopachrome delta-isomerase, tyrosine-related protein 2)	-3.374	7.80E-08
Bt.23651.1.A1_at	DDAH1	dimethylarginine dimethylaminohydrolase 1	3.120	3.96E-06
Bt.5132.1.S1_at	DDAH2	dimethylarginine dimethylaminohydrolase 2	5.513	5.10E-09
Bt.6361.1.S1_at	DDX5	DEAD (Asp-Glu-Ala-Asp) box polypeptide 5	3.700	5.99E-07
Bt.13125.1.S1_s_at	DEFB4A	defensin, beta 4A	3.036	2.57E-02
Bt.26797.1.S1_at	DENND2D	DENN/MADD domain containing 2D	-3.523	3.74E-07
Bt.23557.1.S1_at	DENND3	DENN/MADD domain containing 3	4.137	1.76E-06
Bt.19907.1.A1_at	DIO1	deiodinase, iodothyronine, type I	-3.153	4.32E-05
Bt.2506.1.S1_at	DKK3	dickkopf homolog 3 (Xenopus laevis)	17.396	2.55E-04

Bt.18571.3.A1_at	DLGAP4	discs, large (Drosophila) homolog-associated protein 4	5.843	2.40E-08
Bt.28288.1.S1_at	DNAJB1	DnaJ (Hsp40) homolog, subfamily B, member 1	-3.148	1.51E-04
Bt.20310.1.S1_at	DOCK5	dedicator of cytokinesis 5	3.117	3.24E-06
Bt.2069.2.S1_at	DOK2	docking protein 2, 56kDa	-3.401	9.39E-07
Bt.6145.1.S1_at	DPH3	DPH3, KTI11 homolog (S. cerevisiae)	3.706	3.01E-08
Bt.21419.1.S1_at	DPH5	DPH5 homolog (S. cerevisiae)	3.029	8.12E-04
Bt.161.1.S2_at	DSG1	desmoglein 1	-3.131	2.67E-05
Bt.15705.1.S2_at	DSTN	destrin (actin depolymerizing factor)	4.025	5.96E-06
Bt.1658.1.S1_at	DUSP1	dual specificity phosphatase 1	4.284	3.12E-05
Bt.20043.3.A1_at	DUSP14	Dual specificity phosphatase 14	-3.033	5.55E-06
Bt.3312.1.S1_at	DUSP7	dual specificity phosphatase 7	3.627	1.98E-08
Bt.20800.1.S1_at	DYNLT3	dynein, light chain, Tctex-type 3	3.123	7.48E-05
Bt.1752.1.A1_at	E4F1	E4F transcription factor 1	3.869	1.08E-07

Bt.11352.1.S1_at	EBNA1BP2	EBNA1 binding protein 2	3.101	1.98E-05
Bt.28366.2.S1_at	ECT2	epithelial cell transforming sequence 2 oncogene	-4.159	6.53E-04
Bt.4719.1.S1_at	EDNRA	endothelin receptor type A	-3.252	1.63E-04
Bt.11650.1.A1_at	EEF1A1	eukaryotic translation elongation factor 1 alpha 1	-3.109	1.39E-08
Bt.25624.1.A1_at	EEFSEC	eukaryotic elongation factor, selenocysteine-tRNA-specific	-3.291	4.21E-07
Bt.19329.2.S1_at	EFHD1	EF-hand domain family, member D1	-3.629	1.24E-07
Bt.19329.1.A1_at	EFHD1	EF-hand domain family, member D1	-5.450	5.23E-04
Bt.15561.1.S1_at	EFHD2	EF-hand domain family, member D2	5.779	4.65E-07
Bt.20476.1.S1_at	EFNA4	ephrin-A4	4.179	1.87E-06
Bt.3278.1.A1_at	EFNA5	ephrin-A5	3.340	3.90E-04
Bt.15704.2.S1_at	EGLN1	Egl nine homolog 1 (C. elegans)	3.457	1.35E-05
Bt.22265.1.S1_at	EGR1	early growth response 1	11.284	2.34E-04

Bt.7129.1.S1_at	EHMT2	euchromatic histone-lysine N-methyltransferase 2	-3.160	1.21E-08
Bt.21365.2.S1_at	EIF2AK4	eukaryotic translation initiation factor 2 alpha kinase 4	3.506	2.75E-08
Bt.21365.3.S1_at	EIF2AK4	eukaryotic translation initiation factor 2 alpha kinase 4	3.204	1.83E-05
Bt.18267.1.A1_at	EIF4G3	eukaryotic translation initiation factor 4 gamma, 3	3.217	1.93E-04
Bt.7704.1.S1_at	ELK1	ELK1, member of ETS oncogene family	-3.992	3.17E-06
Bt.16985.1.S1_at	ELMO1	engulfment and cell motility 1	3.103	2.56E-05
Bt.16985.2.S1_at	ELMO1	engulfment and cell motility 1	-3.665	3.37E-07
Bt.26235.1.A1_at	ELMO1	Engulfment and cell motility 1	-5.379	5.79E-07
Bt.26400.1.A1_at	ELMO2	engulfment and cell motility 2	3.095	1.07E-07
Bt.4582.1.A1_at	ELMOD3	ELMO/CED-12 domain containing 3	-3.967	1.41E-07
Bt.21772.1.A1_at	ELTD1	EGF, latrophilin and seven transmembrane domain containing 1	6.139	2.02E-05
Bt.6599.1.S1_at	EMID1	EMI domain containing 1	-4.295	4.79E-04

Bt.16265.1.S1_at	EML1	echinoderm microtubule associated protein like 1	4.665	3.07E-07
Bt.20090.1.S1_at	ENO2	enolase 2 (gamma, neuronal)	3.078	9.29E-05
Bt.1710.1.A1_at	ENSA	endosulfine alpha	-3.396	1.40E-08
Bt.4353.2.S1_at	EPAS1	endothelial PAS domain protein 1	4.582	3.37E-07
Bt.134.1.S1_at	EPB42	erythrocyte membrane protein band 4.2	-3.378	1.37E-08
Bt.21962.1.S1_at	EPDR1	ependymin related protein 1 (zebrafish)	-3.076	4.25E-05
Bt.599.1.S1_at	EPYC	epiphycan	-4.260	4.91E-07
Bt.7620.1.S1_at	ERCC6L	excision repair cross-complementing rodent repair deficiency, complementation group 6-like	-3.595	2.46E-03
Bt.25738.1.A1_at	ERP27	endoplasmic reticulum protein 27	-3.040	1.39E-05
Bt.23905.1.A1_at	ERRFI1	ERBB receptor feedback inhibitor 1	6.105	6.21E-06
Bt.23905.2.S1_at	ERRFI1	ERBB receptor feedback inhibitor 1	4.746	1.53E-05
Bt.12425.1.S1_at	ETNK2	ethanolamine kinase 2	-3.630	8.65E-05

Bt.24447.2.S1_at	F2RL2	coagulation factor II (thrombin) receptor-like 2	-5.893	9.27E-07
Bt.97.1.S1_at	FABP4	fatty acid binding protein 4, adipocyte	3.140	1.02E-03
Bt.22869.1.S1_at	FABP5	fatty acid binding protein 5 (psoriasis-associated)	8.908	1.87E-05
Bt.26318.1.S1_a_at	FAIM	Fas apoptotic inhibitory molecule	3.822	2.80E-06
Bt.7067.1.A1_at	FAM119B	family with sequence similarity 119, member B	-3.549	8.37E-07
Bt.26620.1.S1_at	FAM160A2	family with sequence similarity 160, member A2	3.102	2.13E-08
Bt.9547.1.S2_at	FAM32A	family with sequence similarity 32, member A	3.516	9.14E-07
Bt.28502.1.S1_at	FAM83D	Protein FAM83D	-3.360	2.50E-03
Bt.25036.1.A1_at	FAT1	FAT tumor suppressor homolog 1 (Drosophila)	-3.638	5.12E-08
Bt.2860.1.S1_at	FBLIM1	filamin binding LIM protein 1	3.020	5.20E-05
Bt.18456.1.S1_at	FBLN2	fibulin 2	8.719	7.09E-06
Bt.5021.1.S1_at	FBN1	fibrillin 1	3.791	1.39E-04
Bt.18639.1.A1_at	FBXO32	F-box protein 32	3.334	2.95E-04

Bt.13844.2.S1_at	FBXO33	F-box protein 33	6.154	1.80E-08
Bt.13844.3.A1_at	FBXO33	F-box protein 33	4.543	1.27E-08
Bt.986.1.A1_at	FBXO5	F-box protein 5	-3.469	4.01E-03
Bt.2941.2.S1_a_at	FERMT2	fermitin family member 2	5.355	3.83E-06
Bt.12991.1.A1_at	FGF10	fibroblast growth factor 10	-3.428	3.94E-06
Bt.279.1.S1_at	FGFBP1	fibroblast growth factor-binding protein (FGF-BP)	-3.138	3.35E-06
Bt.29696.1.A1_at	FGFR2	fibroblast growth factor receptor 2	-8.407	1.10E-07
Bt.4451.1.S1_at	FHL2	four and a half LIM domains 2	3.690	1.13E-05
Bt.6154.1.S1_at	FHL3	four and a half LIM domains 3	9.022	9.01E-08
Bt.25882.1.A1_at	FHL3	four and a half LIM domains 3	-4.658	1.92E-06
Bt.24819.1.S1_at	FHOD3	formin homology 2 domain containing 3	3.367	7.32E-05
Bt.13096.1.S1_at	FIGF	c-fos induced growth factor (vascular endothelial growth factor D)	-3.361	3.68E-07

Bt.25955.1.A1_at	FIGNL1	fidgetin-like 1	-3.029	3.37E-07
Bt.24928.1.S1_at	FILIP1L	filamin A interacting protein 1-like	5.617	2.14E-06
Bt.7646.1.S1_at	FLNB	filamin B, beta	6.630	9.26E-07
Bt.2942.1.S1_at	FLRT2	KIAA0405-like	3.285	1.70E-05
Bt.24526.1.S1_at	FLVCR2	feline leukemia virus subgroup C cellular receptor family, member 2	-3.143	4.30E-05
Bt.25090.1.A1_at	FMO2	flavin containing monooxygenase 2 (non-functional)	-4.353	9.10E-08
Bt.23418.1.S1_at	FN1	fibronectin 1	3.906	6.16E-03
Bt.19330.1.A1_s_at	FNBP1L	formin binding protein 1-like	3.726	5.30E-07
Bt.2899.1.S2_at	FOS	FBJ murine osteosarcoma viral oncogene homolog	3.701	9.08E-03
Bt.4441.1.S1_at	FOSL2	FOS-like antigen 2	11.719	1.66E-06
Bt.18887.3.A1_at	FOXO1	forkhead box O1	3.606	1.44E-04
Bt.17019.1.A1_at	FOXP1	Forkhead box P1	4.489	1.20E-04

Bt.12445.1.A1_at	FSCN1	fascin homolog 1, actin-bundling protein (Strongylocentrotus purpuratus)	4.235	1.22E-06
Bt.406.1.S2_at	FSHB	follicle stimulating hormone, beta polypeptide	-4.009	5.53E-07
Bt.4919.1.S1_at	FSHR	follicle stimulating hormone receptor	-4.395	2.35E-03
Bt.405.1.S1_at	FST	follistatin	-4.017	1.74E-03
Bt.7919.1.A1_at	FXYD7	FXYD domain containing ion transport regulator 7	-4.300	3.30E-08
Bt.2359.1.A1_at	FYN	FYN oncogene related to SRC, FGR, YES	3.213	4.65E-04
Bt.20032.1.A1_at	FYTTD1	forty-two-three domain containing 1	3.035	1.75E-04
Bt.3211.1.S1_at	GABARAPL1	GABA(A) receptor-associated protein like 1	6.221	5.98E-07
Bt.3211.1.S2_at	GABARAPL1	GABA(A) receptor-associated protein like 1	3.929	2.94E-05
Bt.6446.1.S1_at	GADD45A	growth arrest and DNA-damage-inducible, alpha	11.005	9.37E-06
Bt.176.1.S1_at	GAL	galanin prepropeptide	10.567	4.04E-07
Bt.24402.1.S1_at	GALNT13	UDP-N-acetyl-alpha-D-galactosamine:polypeptide N-acetylgalactosaminyltransferase	-3.394	1.94E-06

Bt.15184.1.A1_at	GC	group-specific component (vitamin D binding protein)	-3.403	5.43E-07
Bt.12083.2.S1_at	GCA	Grancalcin, EF-hand calcium binding protein	-3.219	3.41E-07
Bt.16068.1.A1_at	GCLC	Glutamate-cysteine ligase, catalytic subunit	-6.751	3.20E-04
Bt.521.1.S1_at	GCNT1	glucosaminyl (N-acetyl) transferase 1, core 2	-3.529	3.37E-07
Bt.26100.2.A1_at	GDPD1	glycerophosphodiester phosphodiesterase domain containing 1	3.647	7.76E-07
Bt.28062.1.S1_at	GEM	GTP binding protein overexpressed in skeletal muscle	4.399	1.03E-03
Bt.2768.3.S1_at	GEMIN8	gem (nuclear organelle) associated protein 8	3.077	2.66E-03
Bt.8781.1.S1_at	GFPT2	glutamine-fructose-6-phosphate transaminase 2	7.809	5.01E-06
Bt.1088.1.S1_a_at	GIMAP7	GTPase, IMAP family member 7	-3.874	2.90E-05
Bt.22016.1.S1_at	GIMAP8	GTPase, IMAP family member 8	7.462	2.03E-07
Bt.14379.2.S1_at	GINS3	GINS complex subunit 3 (Psf3 homolog)	-3.739	2.44E-09
Bt.12684.2.S1_at	GIT2	G protein-coupled receptor kinase interacting ArfGAP 2	4.369	1.74E-06

Bt.5599.1.S1_at	GLIPR2	GLI pathogenesis-related 2	5.578	5.73E-07
Bt.2877.1.S1_at	GLRXL	glutaredoxin (thioltransferase)-like	5.449	7.61E-09
Bt.1429.1.S1_a_at	GLTSCR2	glioma tumor suppressor candidate region gene 2	3.659	4.09E-03
Bt.4665.1.S1_at	GLYCAM1	glycosylation-dependent cell adhesion molecule 1	-3.908	1.92E-06
Bt.29838.1.A1_at	GlyT-1Ab	glycine transporter	-3.242	2.36E-08
Bt.25628.1.A1_at	GMFG	glia maturation factor, gamma	-3.004	7.09E-06
Bt.4734.1.S1_at	GNA14	guanine nucleotide binding protein (G protein), alpha 14	-3.031	2.13E-08
Bt.4586.1.S1_at	GNG2	guanine nucleotide binding protein (G protein), gamma 2	7.330	1.46E-04
Bt.23468.2.S1_at	GOLPH3L	golgi phosphoprotein 3-like	4.762	1.50E-06
Bt.29078.1.A1_at	GOSR1	golgi SNAP receptor complex member 1	-3.225	1.11E-07
Bt.27428.1.A1_at	GPM6A	Glycoprotein M6A	4.791	1.57E-06
Bt.22676.2.S1_at	GPN3	GPN-loop GTPase 3	3.215	2.32E-03

Bt.29666.1.A1_at	GPR128	G protein-coupled receptor 128	-3.574	2.57E-05
Bt.16545.1.A1_at	GPR155	G protein-coupled receptor 155	3.282	3.42E-08
Bt.25609.1.A1_at	GPR61	G protein-coupled receptor 61	-3.554	1.65E-06
Bt.26157.1.A1_at	GPR77	G protein-coupled receptor 77	-3.539	4.10E-05
Bt.29528.1.A1_at	GPRC5A	G protein-coupled receptor, family C, group 5, member A	-4.202	9.96E-07
Bt.24517.2.A1_at	GPRC5B	G protein-coupled receptor, family C, group 5, member B	3.307	7.53E-04
Bt.8998.3.S1_a_at	GPS2	G protein pathway suppressor 2	3.108	4.65E-04
Bt.6557.1.S1_at	GPSM3	G-protein signaling modulator 3 (AGS3-like, C. elegans)	-3.227	1.83E-05
Bt.9623.1.A1_at	GPT	glutamic-pyruvate transaminase (alanine aminotransferase)	-4.088	7.67E-04
Bt.29621.1.S1_at	GPX2	glutathione peroxidase 2	-5.048	1.22E-08
Bt.12916.1.S1_at	GPX3	glutathione peroxidase 3 (plasma)	-5.363	7.62E-04
Bt.12906.1.S1_at	GSN	gelsolin	5.290	4.47E-05

Bt.4852.1.S1_at	GSTM1	glutathione S-transferase M1	-3.147	1.28E-06
Bt.20425.1.A1_at	GTF2E2	general transcription factor IIE, polypeptide 2, beta 34kDa	3.298	4.30E-06
Bt.7612.1.S1_at	GTF2F2	general transcription factor IIF, polypeptide 2, 30kDa	4.389	1.53E-05
Bt.9429.1.S1_at	GTF2H4	general transcription factor IIH, polypeptide 4, 52kDa	3.291	2.20E-06
Bt.178.1.S1_at	GUCA1A	guanylate cyclase activator 1A (retina)	-3.563	1.09E-02
Bt.28070.1.S1_at	GUCA2A	guanylate cyclase activator 2A (guanylin)	-3.803	3.24E-07
Bt.590.1.S1_at	GUCY2F	guanylate cyclase 2F, retinal	-4.644	2.39E-08
Bt.20280.1.S1_at	GYLTL1B	glycosyltransferase-like 1B	-5.647	1.27E-03
Bt.1763.1.S1_at	HAND1	heart and neural crest derivatives expressed 1	-3.770	2.01E-07
Bt.18248.1.A1_at	HAUS4	HAUS augmin-like complex, subunit 4	-9.663	2.72E-08
Bt.13341.1.S1_at	HDAC5	histone deacetylase 5	3.459	1.27E-05
Bt.2919.1.S1_a_at	HDAC7	histone deacetylase 7	5.765	1.35E-06

Bt.8257.2.A1_at	HEATR1	hypothetical LOC508697	-3.788	2.89E-08
Bt.16127.1.S1_at	HECA	headcase homolog (Drosophila)	3.782	4.94E-08
Bt.24694.1.A1_at	HEG1	HEG homolog 1 (zebrafish)	-3.069	1.65E-02
Bt.27920.1.A1_at	HHEX	hematopoietically expressed homeobox	4.188	3.41E-04
Bt.17179.1.S1_at	HJURP	Holliday junction recognition protein	-5.558	3.63E-03
Bt.5928.1.S1_at	HMBOX1	homeobox containing 1	4.034	8.26E-06
Bt.22763.1.S1_at	HMGCS1	3-hydroxy-3-methylglutaryl-Coenzyme A synthase 1 (soluble)	-6.880	4.15E-06
Bt.22763.2.S1_a_at	HMGCS1	3-hydroxy-3-methylglutaryl-Coenzyme A synthase 1 (soluble)	-3.681	6.95E-04
Bt.1518.1.S1_at	HMMR	hyaluronan-mediated motility receptor (RHAMM)	-3.803	2.25E-03
Bt.12915.1.S1_at	HNRNPH3	heterogeneous nuclear ribonucleoprotein H3 (2H9)	3.174	2.08E-07
Bt.17183.1.A1_at	HOXB2	homeobox B2	-4.560	2.46E-07
Bt.8132.1.S1_at	HRG	histidine-rich glycoprotein	-4.567	1.53E-07

Bt.22879.1.S1_at	HSD17B1	hydroxysteroid (17-beta) dehydrogenase 1	-4.849	1.65E-02
Bt.23179.1.S1_at	HSP90AA1	heat shock 90kD protein 1, alpha	4.020	8.58E-03
Bt.5154.1.S1_at	HSPA1A	heat shock 70kDa protein 1A	-5.653	1.31E-06
Bt.23161.2.A1_at	HSPA1A	heat shock 70kDa protein 1A	-6.919	7.48E-07
Bt.5154.1.S1_s_at	HSPA1A	heat shock 70kDa protein 1A	-7.084	3.53E-06
Bt.27992.1.S1_at	HTR1D	5-hydroxytryptamine (serotonin) receptor 1D	-3.152	6.05E-09
Bt.27990.1.S1_at	HTR2A	5-hydroxytryptamine (serotonin) receptor 2A	-4.389	9.61E-07
Bt.12807.1.A1_at	HTR4	5-hydroxytryptamine (serotonin) receptor 4	-3.002	1.40E-06
Bt.27409.2.S1_at	HYDIN	hydrocephalus inducing homolog (mouse)	-3.310	6.88E-08
Bt.23079.2.A1_at	HYOU1	Hypoxia up-regulated 1	-3.054	1.97E-08
Bt.1730.1.A1_at	ID1	inhibitor of DNA binding 1, dominant negative helix-loop-helix protein	7.399	9.79E-07
Bt.5230.1.S1_at	ID3	inhibitor of DNA binding 3, dominant negative helix-loop-helix protein	5.787	7.47E-06

Bt.2153.1.S1_at	IER3	immediate early response 3	14.817	1.01E-08
Bt.1548.1.S1_at	IFI30	gamma-inducible protein 30	4.547	3.54E-04
Bt.24795.1.A1_at	IFIT2	interferon-induced protein with tetratricopeptide repeats 2	-4.011	1.10E-07
Bt.22978.2.S1_at	IFITM1	interferon induced transmembrane protein 1 (9-27)	4.275	1.31E-03
Bt.7671.1.S1_at	IFITM3	interferon induced transmembrane protein 3 (1-8U)	3.292	7.15E-04
Bt.12750.1.S1_at	IGF1	insulin-like growth factor 1 (somatomedin C)	-3.060	2.78E-08
Bt.7676.3.S1_at	IGF2	insulin-like growth factor 2 (somatomedin A)	5.015	1.61E-05
Bt.4977.1.S1_at	IGF2R	insulin-like growth factor 2 receptor	6.199	1.27E-08
Bt.4977.1.S2_at	IGF2R	insulin-like growth factor 2 receptor	4.428	1.60E-07
Bt.21465.1.S1_at	IGFBP5	insulin-like growth factor binding protein 5	4.334	1.24E-06
Bt.9958.1.S1_at	IGFBP6	insulin-like growth factor binding protein 6	5.064	1.98E-04
Bt.12490.1.S1_at	IGHG1	immunoglobulin heavy constant gamma 1	-3.656	1.33E-07

Bt.21368.1.S1_s_at	IGL@	immunoglobulin light chain, lambda gene cluster	-3.743	3.37E-07
Bt.22104.1.S1_at	IHH	Indian hedgehog	-7.115	2.11E-03
Bt.12566.1.S1_at	IL17RA	Interleukin 17 receptor A	3.518	1.70E-06
Bt.19753.2.A1_at	IL17RE	interleukin 17 receptor E	-5.456	5.11E-08
Bt.234.1.S1_at	IL18	interleukin 18 (interferon-gamma-inducing factor)	8.946	1.61E-04
Bt.29883.1.A1_at	IL18R1	interleukin 18 receptor 1	-4.606	1.98E-08
Bt.191.1.S2_at	IL1A	interleukin 1, alpha	-3.060	1.98E-06
Bt.29391.1.S1_at	IL20RA	interleukin 20 receptor, alpha	-3.155	1.16E-04
Bt.23583.1.S1_at	IL21	interleukin 21	-3.998	6.64E-07
Bt.12751.1.S1_at	IL7	interleukin 7	-3.635	2.36E-08
Bt.17150.1.A1_at	ILF3	interleukin enhancer binding factor 3, 90kDa	3.029	4.46E-04
Bt.4864.3.S1_a_at	ING4	inhibitor of growth family, member 4	4.407	1.26E-05
Bt.4897.1.S1_at	INHA	inhibin, alpha	-3.806	8.83E-04

Bt.12760.1.S1_at	INHBA	inhibin, beta A	-4.152	9.85E-03
Bt.24614.1.S1_at	INSIG1	insulin induced gene 1	-3.788	2.54E-04
Bt.20716.1.S1_at	INSIG2	insulin induced gene 2	5.421	8.21E-08
Bt.28097.1.S1_at	INTS7	integrator complex subunit 7	3.294	6.29E-06
Bt.11264.1.S1_at	IP6K2	inositol hexakisphosphate kinase 2	3.022	5.96E-05
Bt.25055.1.A1_at	IPMK	inositol polyphosphate multikinase	-3.225	1.58E-05
Bt.19782.2.S1_a_at	IRAK1BP1	interleukin-1 receptor-associated kinase 1 binding protein 1	4.437	1.70E-07
Bt.22275.1.A1_at	ISG20	interferon stimulated exonuclease gene 20kDa	7.828	2.07E-06
Bt.13039.1.S1_at	ITGB3	integrin, beta 3 (platelet glycoprotein IIIa, antigen CD61)	-3.135	5.20E-08
Bt.25733.1.A1_at	ITGB3	integrin, beta 3 (platelet glycoprotein IIIa, antigen CD61)	-3.227	1.87E-06
Bt.6125.1.S1_at	JAG1	jagged 1	3.645	1.54E-05
Bt.9115.1.S1_at	JAK1	Janus kinase 1	5.746	4.52E-08

Bt.19810.1.A1_at	JAKMIP1	janus kinase and microtubule interacting protein 1	-4.587	8.13E-04
Bt.649.1.S1_at	JAM3	junctional adhesion molecule 3	3.230	1.07E-06
Bt.7008.1.S1_at	JARID2	jumonji, AT rich interactive domain 2	4.894	1.47E-07
Bt.29819.1.A1_at	JSP.1	MHC Class I JSP.1	-3.188	6.09E-07
Bt.11159.1.S1_at	JUN	jun proto-oncogene	6.355	2.42E-05
Bt.9153.1.S1_at	JUN	jun proto-oncogene	3.231	9.25E-06
Bt.20542.1.S1_at	JUNB	jun B proto-oncogene	3.897	4.41E-05
Bt.7727.1.S1_at	KANK2	KN motif and ankyrin repeat domains 2	5.061	2.61E-06
Bt.27801.1.A1_at	KCNE1	potassium voltage-gated channel, Isk-related family, member 1	-3.429	5.33E-07
Bt.24395.2.S1_at	KCNIP2	Kv channel interacting protein 2	-3.050	7.52E-07
Bt.27605.2.S1_at	KCNJ15	potassium inwardly-rectifying channel, subfamily J, member 15	-3.530	1.27E-08
Bt.126.1.S2_at	KCNJ2	potassium inwardly-rectifying channel, subfamily J, member 2	3.663	7.06E-03

Bt.19643.2.S1_at	KCNMB4	potassium large conductance calcium-activated channel, subfamily M, beta member	5.655	3.48E-04
Bt.3275.1.S1_at	KCTD10	potassium channel tetramerisation domain containing 10	3.819	2.03E-07
Bt.19148.1.S1_a_at	KHDRBS1	KH domain containing, RNA binding, signal transduction associated 1	3.037	7.35E-04
Bt.26111.1.A1_at	KIAA1549	hypothetical protein LOC512679	-3.375	8.31E-06
Bt.18081.1.A1_at	KIF20A	kinesin family member 20A	-3.623	9.96E-04
Bt.13476.1.S1_at	KIF23	kinesin family member 23	-3.320	3.49E-03
Bt.1440.1.S1_at	KIFC1	kinesin family member C1	-7.189	4.10E-06
Bt.11174.1.S1_at	KIR2DL5A	killer cell immunoglobulin-like receptor, two domains, long cytoplasmic tail, 5A	-3.117	7.10E-06
Bt.5626.3.S1_at	KLC1	kinesin light chain 1	3.377	8.52E-06
Bt.10851.1.S1_at	KLC2	kinesin light chain 2	-4.265	4.52E-08
Bt.24522.3.S1_a_at	KLF6	Kruppel-like factor 6	6.482	6.34E-07
Bt.4606.1.S1_at	KLF6	Kruppel-like factor 6	4.761	5.79E-05

Bt.12613.1.S1_at	KLF6	Kruppel-like factor 6	4.363	4.37E-06
Bt.4606.2.S1_at	KLF6	Kruppel-like factor 6	4.135	4.90E-04
Bt.3191.1.A1_at	KLHL24	kelch-like 24 (Drosophila)	3.921	2.40E-04
Bt.24380.1.A1_at	KLHL28	kelch-like 28 (Drosophila)	4.868	3.30E-08
Bt.25880.1.A1_at	KLHL29	kelch-like 29 (Drosophila)	-5.184	1.68E-06
Bt.25973.1.S1_at	KLHL33	kelch-like 33 (Drosophila)	-4.567	2.40E-07
Bt.26807.1.S1_at	KLHL36	kelch-like 36 (Drosophila)	3.172	4.05E-08
Bt.19101.1.A1_at	KLK4	kallikrein-related peptidase 4	-4.092	1.75E-06
Bt.1745.1.S1_at	KRT18	keratin 18	62.395	5.98E-07
Bt.23608.1.S1_s_at	KRT8	keratin 8	53.200	2.72E-06
Bt.1742.1.S1_at	LAMB1	laminin, beta 1	12.038	7.54E-06
Bt.6509.2.S1_at	LAMC2	Laminin, gamma 2	-3.293	3.26E-07
Bt.6509.3.S1_a_at	LAMC2	laminin, gamma 2	-4.368	1.81E-04

Bt.6509.1.S1_at	LAMC2	laminin, gamma 2	-6.449	9.55E-04
Bt.27230.1.S1_at	LAMC3	Laminin, gamma 3	-3.083	3.63E-08
Bt.25379.1.A1_at	LARP7	La ribonucleoprotein domain family, member 7	3.310	9.25E-06
Bt.26847.1.S1_a_at	LAT	linker for activation of T cells	-3.306	7.01E-06
Bt.4553.1.S1_at	LDB1	LIM domain binding 1	-4.206	6.33E-08
Bt.6527.1.S1_at	LEF1	lymphoid enhancer-binding factor 1	3.417	3.29E-05
Bt.25543.1.A1_at	LEMD2	LEM domain containing 2	-3.514	1.12E-05
Bt.21031.1.S1_at	LEO1	Leo1, Paf1/RNA polymerase II complex component, homolog (S. cerevisiae)	3.268	3.63E-04
Bt.26572.1.S1_at	LEPREL1	leprecan-like 1	12.818	1.79E-06
Bt.1416.1.S1_at	LGALS3	lectin, galactoside-binding, soluble, 3	22.902	1.48E-06
Bt.21456.1.S1_at	LHFP	lipoma HMGIC fusion partner	3.641	5.57E-04
Bt.15732.1.S1_a_at	LHFPL2	lipoma HMGIC fusion partner-like 2	3.165	2.24E-03

Bt.3399.1.S1_at	LHPP	phospholysine phosphohistidine inorganic pyrophosphate phosphatase	-3.563	9.49E-08
Bt.9286.1.S1_at	LITAF	lipopolysaccharide-induced TNF factor	5.630	1.69E-06
Bt.12411.1.S1_at	LMNA	lamin A/C	3.526	1.60E-04
Bt.22081.1.S1_at	LMO7	LIM domain 7	7.889	9.62E-05
Bt.28602.1.S1_at	LMTK2	basic helix-loop-helix family, member a15-like	8.055	2.29E-08
Bt.5362.2.S1_a_at	LOC100138846	endopin 2C-like	-4.707	2.61E-06
Bt.21851.2.S1_at	LOC100140338	Similar to nuclear antigen Sp100	-3.596	7.84E-09
Bt.21142.1.S1_at	LOC100140348	hypothetical protein LOC100140348	7.188	3.15E-07
Bt.2289.1.S1_at	LOC100140430	DEAD (Asp-Glu-Ala-Asp) box polypeptide 42-like	3.552	5.61E-06
Bt.11047.2.S1_at	LOC100140853	nuclear receptor binding SET domain protein 1	-3.134	6.74E-08
Bt.18745.1.A1_at	LOC100140857	hypothetical protein LOC100140857	-4.530	7.32E-10
Bt.14198.1.S1_at	LOC100270756	hypothetical protein LOC100270756	4.440	1.11E-07

Bt.5281.3.S1_at	LOC100271850	hypothetical protein LOC100271850	3.080	4.98E-07
Bt.6669.1.A1_at	LOC100295338	similar to homeo box D9	-3.219	5.66E-05
Bt.17085.1.A1_at	LOC100297608	hypothetical protein LOC100297608	-5.337	6.26E-06
Bt.19866.1.A1_at	LOC100299267	similar to pyruvate dehydrogenase phosphatase	-3.939	5.08E-06
Bt.15677.1.A1_at	LOC100299693	hypothetical protein LOC100299693	7.087	1.37E-05
Bt.16994.2.A1_at	LOC100300349	similar to scaffold attachment factor B2	3.464	3.66E-03
Bt.25509.1.A1_at	LOC100300920	hypothetical protein LOC100300920	-4.164	2.39E-07
Bt.21252.2.S1_at	LOC100335170	hypothetical protein LOC100335170	-3.056	6.56E-07
Bt.28465.1.S1_at	LOC100335318	envelope glycoprotein-like	6.243	2.53E-05
Bt.28735.1.A1_at	LOC100335318	envelope glycoprotein-like	4.355	1.29E-02
Bt.26650.1.S1_at	LOC100335863	RNA binding motif protein 24-like	4.620	8.76E-04
Bt.5889.1.S1_at	LOC100335863	RNA binding motif protein 24-like	3.995	5.79E-03
Bt.6983.1.S1_at	LOC100336166	transcription factor SOX9-like	8.399	1.93E-05

Bt.17040.1.A1_at	LOC100337079	prolactin-inducible protein homolog-like	-3.007	1.88E-06
Bt.24413.1.S1_at	LOC100337258	transmembrane and tetratricopeptide repeat containing 2-like	3.858	1.25E-05
Bt.4389.1.S1_at	LOC404103	spleen trypsin inhibitor	-34.148	2.44E-09
Bt.21222.1.S1_at	LOC505156	transmembrane protein 131-like	3.168	6.10E-06
Bt.29113.1.A1_at	LOC505468	similar to cytochrome P450 2C92	-5.221	3.93E-07
Bt.24742.1.S1_at	LOC505683	similar to Tubulin polyglutamylase complex subunit 1 (PGs1)	3.022	3.99E-03
Bt.9049.1.S1_at	LOC505709	erbB-2-like	3.219	1.96E-04
Bt.13896.2.S1_at	LOC506185	hypothetical LOC506185	3.033	1.22E-06
Bt.28180.2.S1_at	LOC506707	similar to complement component 4 binding protein, alpha chain	-4.547	1.80E-08
Bt.5007.1.A1_at	LOC508098	hypothetical LOC508098	6.712	1.84E-04
Bt.25111.1.A1_at	LOC508347	interferon-induced protein 44-like	-3.790	7.53E-04
Bt.25996.1.A1_at	LOC509089	similar to hCG1785352	-3.253	3.53E-09

Bt.11386.2.S1_at	LOC510844	LIM homeobox transcription factor 1, beta-like	-6.312	1.98E-08
Bt.24920.1.S1_at	LOC512005	hypothetical protein	-3.245	9.74E-08
Bt.18718.1.S1_at	LOC514189	hypothetical LOC514189	-3.398	1.77E-06
Bt.11593.1.S1_at	LOC514704	similar to KIAA0232 gene product	4.445	2.17E-07
Bt.7967.1.S1_at	LOC515718	Vav 2 protein	5.570	4.06E-07
Bt.19527.1.S1_at	LOC523963	similar to interferon regulatory factor 2 binding protein 1	-3.150	2.24E-08
Bt.24788.1.A1_at	LOC524159	similar to F11C1.5a	-4.146	1.88E-08
Bt.26470.1.S1_at	LOC524593	similar to opposite strand transcription unit to Stag3	3.383	3.71E-08
Bt.28530.1.A1_at	LOC529462	hypothetical LOC529462	3.380	5.51E-05
Bt.29118.1.A1_at	LOC529746	similar to Uncharacterized protein C14orf145	-3.257	1.45E-05
Bt.5043.1.S1_at	LOC531757	Similar to Aminopeptidase O (AP-O)	3.780	6.27E-06
Bt.13882.1.S1_at	LOC532603	hypothetical protein LOC532603	4.381	3.89E-04

Bt.15812.1.A1_at	LOC533324	similar to Protein FAM126B	4.362	9.48E-06
Bt.20071.2.S1_at	LOC536128	similar to Notch homolog 4 (Drosophila)	-3.369	2.19E-07
Bt.17834.1.A1_at	LOC538060	similar to Aldo-keto reductase family 1, member C3 (3-alpha hydroxysteroid dehydrogenase	-3.540	3.33E-07
Bt.12405.1.S1_at	LOC538276	similar to Janus kinase 3	-5.097	1.73E-03
Bt.26041.1.A1_at	LOC538782	hypothetical LOC538782	3.079	1.33E-04
Bt.18834.1.A1_at	LOC538993	similar to Uncharacterized protein KIAA0748 homolog	-5.416	3.97E-08
Bt.23056.1.S1_s_at	LOC540077	ariadne homolog 2-like	3.964	7.52E-06
Bt.16582.1.A1_at	LOC540627	similar to Cytochrome P450, family 2, subfamily C, polypeptide 87	-3.377	2.12E-08
Bt.3422.1.S1_at	LOC613747	similar to RIKEN cDNA 9130404D14 gene	3.297	5.31E-06
Bt.24110.1.A1_at	LOC614478	hypothetical LOC614478	-5.900	1.39E-07
Bt.28220.1.S1_at	LOC615263	hypothetical protein LOC615263	3.499	2.83E-03
Bt.13798.1.S1_at	LOC615685	UPF0452 protein C7orf41 homolog	4.925	5.15E-07

Bt.13798.2.S1_at	LOC615685	UPF0452 protein C7orf41 homolog	3.553	1.39E-05
Bt.16436.3.A1_at	LOC615685	UPF0452 protein C7orf41 homolog	3.378	1.05E-06
Bt.28732.1.S1_s_at	LOC618831	similar to TRD@ protein	-4.026	1.18E-05
Bt.23094.3.S1_s_at	LOC782061	aldo-keto reductase family 1, member C1-like	-5.673	3.76E-07
Bt.124.1.S1_at	LOC785870	PDYN protein-like	-3.991	1.39E-06
Bt.23205.1.S1_s_at	LOC787239	DKFZP459P193 protein-like	8.460	9.59E-07
Bt.20232.3.S1_at	LOC788113	SWI/SNF-related matrix-associated actin-dependent regulator of chromatin a5-like	-4.309	3.83E-09
Bt.1436.1.S1_at	LOC788843	pregnancy-associated glycoprotein 11-like	-3.050	7.47E-03
Bt.18089.1.S1_at	LOC789240 /// TES	testin	3.664	4.47E-05
Bt.26364.1.A1_at	LOC790155	hypothetical LOC790155	3.558	3.69E-05
Bt.12297.1.S1_at	LOXL4	lysyl oxidase-like 4	6.498	1.87E-05
Bt.5387.1.S1_at	LPL	lipoprotein lipase	-4.639	1.34E-03

Bt.4784.1.S1_at	LPO	lactoperoxidase	-3.854	7.17E-08
Bt.10704.1.S1_at	LPPR2	lipid phosphate phosphatase-related protein type 2	-3.529	5.01E-05
Bt.16499.1.A1_at	LRCH1	Leucine-rich repeats and calponin homology (CH) domain containing 1	3.768	5.47E-07
Bt.4108.1.S1_at	LRRFIP1	leucine rich repeat (in FLII) interacting protein 1	4.714	6.54E-06
Bt.9800.1.A1_a_at	LSG1	large subunit GTPase 1 homolog (S. cerevisiae)	3.589	3.42E-07
Bt.17801.1.A1_at	LSS	Lanosterol synthase (2,3-oxidosqualene-lanosterol cyclase)	-3.029	1.83E-06
Bt.27067.1.A1_at	LTA	lymphotoxin alpha (TNF superfamily, member 1)	-4.774	2.24E-08
Bt.4874.2.S1_a_at	LUC7L3	LUC7-like 3 (S. cerevisiae)	8.856	3.26E-06
Bt.2452.1.S1_at	LUM	lumican	6.110	4.08E-05
Bt.9737.1.S1_at	LY6G6C	lymphocyte antigen 6 complex, locus G6C	-3.071	1.67E-07
Bt.28439.1.S1_a_at	LY9	lymphocyte antigen 9	-3.189	2.61E-06
Bt.209.3.S1_at	LYZ	lysozyme	3.046	4.27E-05

Bt.12986.1.S1_at	MAD2	mitotic checkpoint component Mad2	-3.116	5.63E-03
Bt.19578.1.S1_at	MAFK	v-maf musculoaponeurotic fibrosarcoma oncogene homolog K (avian)	3.328	3.23E-06
Bt.27901.1.S1_at	MAGI1	membrane associated guanylate kinase, WW and PDZ domain containing 1	5.962	3.71E-05
Bt.22647.2.S1_a_at	MAGI3	Membrane associated guanylate kinase, WW and PDZ domain containing 3	4.579	2.44E-07
Bt.28686.1.S1_at	MAL2	mal, T-cell differentiation protein 2	-4.592	1.70E-05
Bt.3751.1.S1_at	MAP1B	microtubule-associated protein 1B	3.999	9.95E-06
Bt.3000.2.S1_a_at	MAP1LC3C	microtubule-associated protein 1 light chain 3 gamma	16.653	1.54E-05
Bt.13769.1.S1_at	MAP2	Microtubule-associated protein 2	4.547	4.94E-06
Bt.22071.1.S1_at	MAP2K3	mitogen-activated protein kinase kinase 3	3.095	2.39E-07
Bt.25690.1.S1_at	MAP3K7IP3	mitogen-activated protein kinase kinase kinase 7 interacting protein 3	-3.598	1.98E-08
Bt.12710.2.S1_at	MAP4K1	mitogen-activated protein kinase kinase kinase kinase 1	-4.009	2.71E-07

Bt.20458.1.S1_at	MAP7D1	MAP7 domain containing 1	3.946	8.97E-07
Bt.17898.1.A1_at	MAP9	microtubule-associated protein 9	3.223	2.71E-05
Bt.29852.1.S1_at	MAPT	microtubule-associated protein tau	-3.330	4.81E-07
Bt.5605.2.S1_at	MARCKS	myristoylated alanine-rich protein kinase C substrate	3.695	2.84E-07
Bt.3880.1.S1_at	MARCKSL1	MARCKS-like 1	3.227	2.75E-05
Bt.23307.1.S1_at	MCAM	melanoma cell adhesion molecule	-4.007	8.96E-05
Bt.16556.1.A1_at	MCM10	Minichromosome maintenance complex component 10	-3.290	2.77E-03
Bt.6463.2.A1_at	MDC1	mediator of DNA-damage checkpoint 1	-4.473	8.96E-08
Bt.21528.1.A1_at	MDK	midkine (neurite growth-promoting factor 2)	9.949	1.47E-06
Bt.22026.1.S1_at	MERTK	c-mer proto-oncogene tyrosine kinase	4.102	2.73E-07
Bt.11035.1.S1_at	MESDC1	mesoderm development candidate 1	3.190	1.89E-07
Bt.24275.1.S1_at	METRNL	meteorin, glial cell differentiation regulator-like	3.104	2.52E-06
Bt.22464.2.S1_at	METTL2B	methyltransferase like 2B	-3.429	4.85E-07

Bt.17581.2.S1_at	METTL7B	similar to DILV594	-3.704	6.39E-08
Bt.24589.2.S1_at	MEX3C	mex-3 homolog C (C. elegans)	-4.985	2.95E-05
Bt.28223.1.S1_at	MGC127133	20-beta-hydroxysteroid dehydrogenase-like	-3.224	1.05E-05
Bt.13235.1.S1_at	MGC148992	similar to RGC-32	5.094	1.69E-05
Bt.11622.1.A1_at	MGC160048	Uncharacterized protein ENSP00000360166 homolog	-3.535	1.52E-06
Bt.3595.1.S1_at	MGP	matrix Gla protein	9.541	1.90E-04
Bt.21183.1.S1_at	MGST2	microsomal glutathione S-transferase 2	3.421	5.17E-06
Bt.27718.1.S1_at	MIA3	melanoma inhibitory activity family, member 3	4.611	1.34E-06
Bt.9416.1.S1_at	MICAL1	microtubule associated monoxygenase, calponin and LIM domain containing 1	8.900	1.11E-05
Bt.28443.1.S1_at	MICAL2	microtubule associated monoxygenase, calponin and LIM domain containing 2	3.135	9.87E-04
Bt.28275.1.S1_at	MICALL2	MICAL-like 2	6.124	5.29E-06
Bt.29684.1.A1_at	MICB	MHC class I polypeptide-related sequence B	-3.637	1.24E-06

Bt.11109.1.S1_at	MIR221	microRNA mir-221	5.134	5.30E-07
Bt.27239.1.A1_at	MLKL	mixed lineage kinase domain-like	-3.271	5.47E-07
Bt.18271.1.A1_at	MLL3	myeloid/lymphoid or mixed-lineage leukemia 4-like	4.691	1.64E-05
Bt.3735.2.S1_at	MLLT11	myeloid/lymphoid or mixed-lineage leukemia (trithorax homolog, Drosophila); tran	3.338	2.44E-03
Bt.13703.1.S1_at	MMP11	matrix metallopeptidase 11 (stromelysin 3)	4.402	4.54E-08
Bt.7179.1.S1_a_at	MMP23B	matrix metallopeptidase 23B	7.698	5.31E-06
Bt.7179.3.S1_a_at	MMP23B	matrix metallopeptidase 23B	3.326	7.29E-06
Bt.18504.2.S1_a_at	MMP3	matrix metallopeptidase 3 (stromelysin 1, progelatinase)	-3.030	1.22E-08
Bt.13092.1.S1_at	MMP7	matrix metallopeptidase 7 (matrilysin, uterine)	-3.448	3.23E-07
Bt.17377.1.S1_at	MOBKL2B	MOB1, Mps One Binder kinase activator-like 2B (yeast)	-4.391	5.10E-04
Bt.26405.1.A1_at	MORF4L2	Mortality factor 4 like 2	3.332	3.06E-05
Bt.25048.1.A1_at	MPDZ	multiple PDZ domain protein	-4.043	6.12E-07

Bt.9506.1.S1_at	MPHOSPH8	M-phase phosphoprotein 8	3.378	8.58E-05
Bt.19581.2.S1_at	MRPS28	mitochondrial ribosomal protein S28	3.627	3.47E-04
Bt.3289.2.S1_at	MTA3	metastasis associated 1 family, member 3	3.936	3.98E-04
Bt.11327.2.A1_at	MTHFSD	Methenyltetrahydrofolate synthetase domain containing	-5.441	1.08E-07
Bt.15922.1.S1_at	MTMR3	myotubularin related protein 3	3.808	2.63E-07
Bt.647.1.S1_at	MTPN	myotrophin	4.646	1.50E-03
Bt.8319.3.S1_a_at	MTUS1	microtubule associated tumor suppressor 1	-3.438	5.32E-05
Bt.11217.2.S1_a_at	MUC15	mucin 15, cell surface associated	-5.735	1.21E-07
Bt.5390.1.S1_at	MVP	major vault protein	3.683	4.21E-07
Bt.20431.1.A1_at	MXI1	MAX interactor 1	4.858	1.97E-06
Bt.20431.3.S1_at	MXI1	MAX interactor 1	3.503	6.16E-06
Bt.25236.1.A1_at	MXRA5	Matrix-remodelling associated 5	5.324	6.25E-04

Bt.4872.1.S1_at	MXRA8	matrix-remodelling associated 8	5.804	4.01E-04
Bt.17630.1.S1_at	MYCBP2	MYC binding protein 2	4.631	5.79E-08
Bt.5283.1.S1_at	MYLK	myosin light chain kinase	6.774	2.48E-05
Bt.29309.1.A1_at	MYO1D	myosin ID	-4.308	2.77E-06
Bt.16088.1.A1_at	MYO5C	myosin VC	-3.036	7.19E-06
Bt.4387.1.S1_at	NAGK	N-acetylglucosamine kinase	3.423	1.94E-08
Bt.6688.3.A1_at	NARS	Asparaginyl-tRNA synthetase	-3.326	1.38E-07
Bt.28346.1.A1_at	NCAPH	non-SMC condensin I complex, subunit H	-3.176	6.66E-03
Bt.26515.2.S1_at	NCOA6	nuclear receptor coactivator 6	-3.206	8.02E-07
Bt.3307.1.A1_at	NDP	Norrie disease (pseudoglioma)	7.588	8.32E-05
Bt.1537.1.S1_at	NDRG1	N-myc downstream regulated 1	8.978	7.86E-08
Bt.2881.1.S1_at	NDRG4	NDRG family member 4	5.556	1.49E-05
Bt.25670.1.A1_at	NFE2L2	Nuclear factor (erythroid-derived 2)-like 2	-3.648	7.61E-09

Bt.22699.3.S1_at	NFIA	Nuclear factor I/A	-3.264	1.50E-05
Bt.27854.1.S1_at	NFIL3	nuclear factor, interleukin 3 regulated	4.980	9.02E-07
Bt.9027.1.S1_at	NFKBIA	nuclear factor of kappa light polypeptide gene enhancer in B-cells inhibitor, al	3.189	1.05E-06
Bt.10065.1.S1_at	NGFR	nerve growth factor receptor	-3.198	7.84E-07
Bt.16087.1.S1_at	NID2	nidogen 2 (osteonidogen)	18.473	5.17E-06
Bt.12285.3.S1_a_at	NMI	N-myc (and STAT) interactor	5.740	9.08E-09
Bt.12285.2.S1_a_at	NMI	N-myc (and STAT) interactor	4.812	6.87E-07
Bt.5129.1.S1_a_at	NNAT	neuronatin	7.807	1.89E-08
Bt.23126.1.S1_at	NOS2	nitric oxide synthase 2, inducible	-3.859	3.41E-04
Bt.8479.1.A1_at	NOS2	nitric oxide synthase 2, inducible	-8.427	7.73E-04
Bt.23268.1.S1_at	NPC2	Niemann-Pick disease, type C2	3.015	2.87E-07
Bt.9754.1.S1_at	NQO1	NAD(P)H dehydrogenase, quinone 1	-3.551	1.92E-04

Bt.543.1.S1_at	NR2F1	nuclear receptor subfamily 2, group F, member 1	4.129	1.23E-04
Bt.10060.1.S1_at	NR4A2	nuclear receptor subfamily 4, group A, member 2	3.516	1.06E-05
Bt.23195.2.S1_at	NR5A1	Nuclear receptor subfamily 5, group A, member 1	-3.165	1.89E-06
Bt.5515.1.S1_at	NT5E	5'-nucleotidase, ecto (CD73)	3.750	3.01E-04
Bt.26919.1.S1_at	NTN4	netrin 4	7.318	1.13E-06
Bt.26074.1.A1_at	NTRK3	neurotrophic tyrosine kinase, receptor, type 3	-4.111	5.52E-08
Bt.26971.1.S1_at	NUAK1	NUAK family, SNF1-like kinase, 1	12.914	4.32E-07
Bt.25412.1.A1_at	NUSAP1	nucleolar and spindle associated protein 1	-4.309	8.88E-03
Bt.560.1.S1_at	NXPH2	neurexophilin 2	-5.368	1.12E-07
Bt.27720.1.A1_at	OASL	2'-5'-oligoadenylate synthetase-like	-5.064	7.12E-08
Bt.9673.1.S1_at	OCIAD2	OCIA domain containing 2	3.471	4.77E-05
Bt.29544.1.A1_at	ODF2L	outer dense fiber of sperm tails 2-like	5.810	2.49E-06
Bt.27143.1.A1_at	ODF2L	Outer dense fiber of sperm tails 2-like	4.215	1.13E-05

Bt.27225.1.A1_at	ODZ3	odz, odd Oz/ten-m homolog 3 (Drosophila)	-3.720	1.88E-08
Bt.28826.1.A1_at	OIP5	Opa interacting protein 5	-3.247	5.38E-03
Bt.25836.1.A1_at	OLFML1	olfactomedin-like 1	-3.005	9.06E-06
Bt.367.1.S1_at	OLR1	oxidized low density lipoprotein (lectin-like) receptor 1	11.732	4.15E-04
Bt.24.1.S1_at	OPCML	opioid binding protein/cell adhesion molecule-like	-3.384	1.69E-07
Bt.7217.1.S1_at	OPN1LW	opsin 1 (cone pigments), long-wave-sensitive	-3.163	3.21E-08
Bt.242.1.S1_at	OPRM1	opioid receptor, mu 1	-4.199	3.02E-06
Bt.26506.1.S1_at	ORF1	ORF1 protein	-3.579	4.15E-06
Bt.2888.1.S1_at	OSTF1	osteoclast stimulating factor 1	3.172	1.13E-05
Bt.16016.1.S1_at	P2RX5	purinergic receptor P2X, ligand-gated ion channel, 5	-3.233	2.43E-06
Bt.9163.2.S1_at	P2RY10	purinergic receptor P2Y, G-protein coupled, 10	-3.180	2.90E-08
Bt.29292.1.A1_at	P4HA2	prolyl 4-hydroxylase, alpha polypeptide II	-3.417	5.80E-08

Bt.26548.1.S1_at	PA1	PAXIP1-associated protein 1	-3.071	3.85E-06
Bt.20356.2.A1_at	PACAP	plasma cell-induced ER protein 1	-10.090	2.23E-08
Bt.18776.1.S1_at	PAF	KIAA0101 protein	-4.597	1.01E-02
Bt.12063.1.S1_at	PCDH7	protocadherin 7	6.994	6.94E-06
Bt.3804.1.S1_at	PCOLCE2	procollagen C-endopeptidase enhancer 2	4.571	1.13E-05
Bt.14155.2.S1_a_at	PDCD10	programmed cell death 10	3.148	8.70E-05
Bt.4770.1.S1_at	PDCD4	programmed cell death 4 (neoplastic transformation inhibitor)	3.892	6.26E-04
Bt.26295.1.A1_at	PDE4D	phosphodiesterase 4D, cAMP-specific	-4.090	2.32E-08
Bt.4147.1.S1_at	PDE6A	phosphodiesterase 6A, cGMP-specific, rod, alpha	-4.438	2.40E-06
Bt.54.1.S1_at	PDE6G	phosphodiesterase 6G, cGMP-specific, rod, gamma	-3.335	7.61E-09
Bt.9423.1.S1_at	PDGFRA	platelet-derived growth factor receptor, alpha polypeptide	26.503	2.30E-06
Bt.13330.1.S1_at	PDK4	pyruvate dehydrogenase kinase, isozyme 4	9.694	7.64E-05

Bt.23505.1.S1_at	PDK4	pyruvate dehydrogenase kinase, isozyme 4	5.586	1.07E-04
Bt.13330.2.A1_at	PDK4	pyruvate dehydrogenase kinase, isozyme 4	3.990	4.02E-04
Bt.1358.1.S1_at	PDLIM1	PDZ and LIM domain 1	3.945	5.87E-04
Bt.23294.1.A1_at	PDLIM3	PDZ and LIM domain 3	3.833	1.61E-04
Bt.10813.1.S1_at	PDLIM4	PDZ and LIM domain 4	4.578	1.15E-04
Bt.12295.1.S1_at	PDPN	podoplanin	4.743	4.92E-05
Bt.7310.1.S1_at	PEAR1	platelet endothelial aggregation receptor 1	-4.092	4.64E-07
Bt.4653.1.S1_at	PECAM1	platelet/endothelial cell adhesion molecule	-3.119	4.11E-06
Bt.5027.1.A1_at	PEG3	paternally expressed 3	3.911	1.03E-03
Bt.6490.2.S1_at	PELI1	pellino homolog 1 (Drosophila)	4.707	5.76E-07
Bt.8473.1.S1_at	PEX11B	peroxisomal biogenesis factor 11 beta	-3.083	4.91E-07
Bt.4216.1.S1_at	PGF	placental growth factor	-4.173	7.89E-03
Bt.21939.2.S1_at	PHACTR1	phosphatase and actin regulator 1	-3.105	8.19E-07

Bt.18551.1.S1_at	PHF20L1	PHD finger protein 20-like 1	4.282	5.65E-06
Bt.10212.1.S1_at	PHLDA1	Pleckstrin homology-like domain, family A, member 1	4.701	2.36E-06
Bt.14658.1.A1_at	PIGT	phosphatidylinositol glycan anchor biosynthesis, class T	4.291	3.83E-09
Bt.447.1.S1_at	PIK3CA	phosphoinositide-3-kinase, catalytic, alpha polypeptide	4.269	9.48E-07
Bt.13689.1.A1_at	PIK3CD	Phosphoinositide-3-kinase, catalytic, delta polypeptide	3.104	1.92E-05
Bt.1457.1.A1_at	PILRA	paired immunoglobin-like type 2 receptor alpha	-3.282	2.38E-06
Bt.272.1.S1_at	PIM1	pim-1 oncogene	4.025	1.73E-06
Bt.24730.1.S1_at	PIPOX	pipecolic acid oxidase	-3.584	3.80E-07
Bt.17496.2.A1_at	PJA2	Praja ring finger 2	-3.264	2.51E-06
Bt.28799.1.S1_at	PKIB	protein kinase (cAMP-dependent, catalytic) inhibitor beta	6.532	4.55E-05
Bt.21835.1.S1_at	PKIG	protein kinase (cAMP-dependent, catalytic) inhibitor gamma	3.608	9.54E-07
Bt.4439.1.S1_at	PLA2G1B	phospholipase A2, group IB (pancreas)	-4.512	1.71E-03

Bt.22208.1.S1_at	PLA2G2D1	calcium-dependent phospholipase A2 PLA2G2D1	-3.096	2.25E-05
Bt.12302.1.S1_at	PLAT	plasminogen activator, tissue	26.494	7.40E-07
Bt.1942.1.S1_at	PLAUR	plasminogen activator, urokinase receptor	6.741	9.20E-05
Bt.27012.1.A1_at	PLCL2	phospholipase C-like 2	3.032	1.04E-03
Bt.15713.1.A1_at	PLEK	Pleckstrin	-3.011	1.67E-02
Bt.15713.2.S1_at	PLEK	pleckstrin	-3.961	9.38E-06
Bt.8690.2.S1_at	PLEKHB2	pleckstrin homology domain containing, family B (evectins) member 2	3.583	3.95E-07
Bt.8690.1.S1_at	PLEKHB2	pleckstrin homology domain containing, family B (evectins) member 2	3.264	1.62E-04
Bt.16173.1.A1_at	PLEKHF2	pleckstrin homology domain containing, family F (with FYVE domain) member 2	-3.796	8.15E-11
Bt.7484.1.S1_at	PLEKHG2	pleckstrin homology domain containing, family G (with RhoGef domain) member 2	3.320	1.14E-07
Bt.20245.1.S1_at	PLEKHO1	pleckstrin homology domain containing, family O member 1	4.816	9.22E-08

Bt.2568.1.S1_at	PLIN2	perilipin 2	6.554	2.56E-06
Bt.13265.1.A1_at	PLLP	plasmolipin	-3.189	4.90E-07
Bt.5293.2.A1_at	PLP1	proteolipid protein 1	-4.640	3.58E-07
Bt.28704.1.S1_at	PLSCR4	phospholipid scramblase 4	5.399	2.81E-06
Bt.25703.1.A1_at	PLXDC2	plexin domain containing 2	9.101	1.00E-06
Bt.3323.2.S1_at	PLXNB2	plexin B2	3.822	6.62E-04
Bt.16053.1.S1_at	PLXND1	plexin D1	3.569	1.28E-06
Bt.27517.1.S1_at	PNLIP	pancreatic lipase	-3.372	6.75E-06
Bt.25452.1.A1_at	PNLIPRP2	pancreatic lipase-related protein 2	-3.485	3.02E-06
Bt.451.1.S1_at	PNMT	phenylethanolamine N-methyltransferase	4.114	3.06E-05
Bt.20991.1.S1_at	POLR3D	polymerase (RNA) III (DNA directed) polypeptide D, 44kDa	-4.007	6.78E-08
Bt.13028.1.S1_at	POSTN	periostin, osteoblast specific factor	9.907	7.88E-05

Bt.180.1.S1_at	POU1F1	POU class 1 homeobox 1	-3.056	1.16E-06
Bt.10179.1.S1_at	PPFIBP1	PTPRF interacting protein, binding protein 1 (liprin beta 1)	3.298	1.69E-04
Bt.20311.1.S1_a_at	PPIE	peptidylprolyl isomerase E (cyclophilin E)	3.111	1.24E-06
Bt.2191.1.A1_at	PPP3CC	Protein phosphatase 3 (formerly 2B), catalytic subunit, gamma isoform	-4.466	9.10E-08
Bt.28700.1.S1_at	PQLC3	PQ loop repeat containing 3	3.872	3.46E-04
Bt.20428.2.S1_a_at	PRC1	protein regulator of cytokinesis 1	-3.294	1.19E-02
Bt.5156.2.S1_at	PRCC	papillary renal cell carcinoma (translocation-associated)	-3.533	4.70E-08
Bt.3202.1.A1_at	PRELP	proline/arginine-rich end leucine-rich repeat protein	-4.482	7.40E-06
Bt.12688.1.A1_at	PRICKLE1	prickle homolog 1 (Drosophila)	8.173	8.42E-07
Bt.13229.1.S1_at	PRKAG2	protein kinase, AMP-activated, gamma 2 non-catalytic subunit	18.044	2.78E-08
Bt.29351.1.A1_at	PRSS22	protease, serine, 22	-4.358	3.57E-06
Bt.20330.1.S1_at	PRSS23	protease, serine, 23	10.864	1.16E-06

Bt.3624.1.S1_at	PSMB10	proteasome (prosome, macropain) subunit, beta type, 10	3.524	1.72E-06
Bt.22634.3.S1_at	PTGFRN	prostaglandin F2 receptor negative regulator	-4.504	5.00E-05
Bt.28518.1.S1_at	PTI	pancreatic trypsin inhibitor	-58.753	2.44E-09
Bt.21812.1.S1_at	PTPN21	protein tyrosine phosphatase, non-receptor type 21	3.159	3.96E-06
Bt.19636.1.A1_at	PTPN5	protein tyrosine phosphatase, non-receptor type 5 (striatum-enriched)	4.596	1.15E-06
Bt.7319.1.S1_at	PTPRK	protein tyrosine phosphatase, receptor type, K	3.578	6.03E-06
Bt.7538.1.S1_at	PTPRN2	protein tyrosine phosphatase, receptor type, N polypeptide 2	3.234	2.47E-03
Bt.3756.1.A1_at	PTTG1	pituitary tumor-transforming 1	-4.012	7.05E-03
Bt.10398.1.S1_at	PTX3	pentraxin 3, long	24.148	1.37E-08
Bt.4943.3.S1_a_at	PVRL2	poliovirus receptor-related 2 (herpesvirus entry mediator B)	3.794	1.20E-04
Bt.1770.2.A1_at	PXDN	peroxidasin homolog (Drosophila)	4.178	9.64E-07
Bt.23682.1.S1_at	PYGL	phosphorylase, glycogen, liver	4.890	7.06E-04

Bt.17714.1.A1_at	PYGL	Phosphorylase, glycogen, liver	3.437	5.05E-03
Bt.23098.1.S1_at	QSOX1	quiescin Q6 sulfhydryl oxidase 1	5.879	9.72E-07
Bt.9652.1.S1_at	RAB22A	RAB22A, member RAS oncogene family	3.287	5.42E-07
Bt.22771.1.S1_at	RAB31	RAB31, member RAS oncogene family	3.243	7.50E-08
Bt.2741.1.A1_at	RAB7A	RAB7A, member RAS oncogene family	3.122	1.27E-05
Bt.26479.1.S1_at	RABEP1	rabaptin, RAB GTPase binding effector protein 1	3.896	5.60E-07
Bt.29005.1.A1_at	RAD51AP1	RAD51 associated protein 1	-4.462	2.04E-03
Bt.20948.1.S1_at	RAD52	RAD52 homolog (S. cerevisiae)	3.505	2.72E-06
Bt.20439.1.S1_at	RALA	v-ral simian leukemia viral oncogene homolog A (ras related)	3.873	7.57E-05
Bt.26628.1.S1_at	RAP2C	RAP2C, member of RAS oncogene family	4.011	1.93E-07
Bt.21170.1.A1_at	RASA2	RAS p21 protein activator 2	4.073	7.77E-06
Bt.9921.1.A1_at	RASAL2	RAS protein activator like 2	4.151	5.26E-05

Bt.6858.1.S1_at	RASGEF1A	RasGEF domain family, member 1A	-3.042	5.40E-07
Bt.28512.1.S1_at	RASGRP4	RAS guanyl releasing protein 4	-4.152	1.20E-08
Bt.8138.1.S1_at	RASSF2	Ras association (RalGDS/AF-6) domain family member 2	-3.188	2.08E-07
Bt.27316.2.A1_at	RBBP8	retinoblastoma binding protein 8	-3.094	2.81E-04
Bt.3013.1.S1_a_at	RBFOX2	RNA binding protein, fox-1 homolog (C. elegans) 2	6.472	8.45E-07
Bt.3013.2.S1_a_at	RBFOX2	RNA binding protein, fox-1 homolog (C. elegans) 2	5.920	4.83E-07
Bt.5821.1.A1_at	RBFOX2	RNA binding protein, fox-1 homolog (C. elegans) 2	3.687	1.30E-07
Bt.13335.1.S1_at	RBKS	ribokinase	3.089	1.61E-04
Bt.10245.2.S1_at	RBL2	retinoblastoma-like 2 (p130)	-3.224	1.41E-07
Bt.28160.2.S1_a_at	RBM17	RNA binding motif protein 17	4.530	4.32E-06
Bt.17614.2.A1_at	RBM25	RNA binding motif protein 25	3.920	2.26E-05
Bt.9571.2.S1_at	RBM3	RNA binding motif (RNP1, RRM) protein 3	3.804	6.05E-05

Bt.24935.2.S1_at	RBMS2	RNA binding motif, single stranded interacting protein 2	6.347	2.50E-07
Bt.17810.1.S1_a_at	RBP1	retinol binding protein 1, cellular	3.429	4.86E-06
Bt.8643.2.S1_at	RCN2	reticulocalbin 2, EF-hand calcium binding domain	4.394	1.40E-05
Bt.4078.2.S1_a_at	RCN3	reticulocalbin 3, EF-hand calcium binding domain	3.675	9.58E-04
Bt.25868.1.A1_at	RCN3	reticulocalbin 3, EF-hand calcium binding domain	-4.499	9.32E-07
Bt.14565.1.A1_at	RELN	reelin	7.706	5.38E-05
Bt.1760.1.S1_at	REXO2	REX2, RNA exonuclease 2 homolog (S. cerevisiae)	4.002	2.39E-05
Bt.22646.2.S1_at	RFX5	regulatory factor X, 5 (influences HLA class II expression)	-3.448	1.22E-08
Bt.10855.1.S1_at	RGS2	regulator of G-protein signaling 2, 24kDa	7.185	5.83E-04
Bt.8030.2.S1_at	RGS3	regulator of G-protein signaling 3	-3.309	3.36E-08
Bt.8856.1.S1_at	RHBG	Rh family, B glycoprotein (gene/pseudogene)	-3.953	8.85E-06
Bt.12753.1.S1_at	RHO	rhodopsin	-5.770	1.98E-08

Bt.12600.1.S1_at	RIC3	Resistance to inhibitors of cholinesterase 3 homolog (C. elegans)	-3.795	6.18E-04
Bt.4884.1.S1_at	RILPL2	Rab interacting lysosomal protein-like 2	3.989	5.14E-05
Bt.19348.1.A1_at	RIOK2	RIO kinase 2 (yeast)	3.189	2.12E-05
Bt.27723.2.A1_a_at	RIPK3	receptor-interacting serine-threonine kinase 3	-4.751	2.78E-09
Bt.1546.1.S1_at	RND3	Rho family GTPase 3	35.684	2.79E-07
Bt.6014.2.S1_at	RNF168	ring finger protein 168	-3.581	7.00E-08
Bt.10397.2.S1_at	RNF20	ring finger protein 20	3.173	8.34E-05
Bt.6578.3.S1_at	RNF8	ring finger protein 8	3.053	6.49E-04
Bt.16125.1.A1_s_at	RNFT1	ring finger protein, transmembrane 1	-3.033	1.06E-06
Bt.16125.1.A1_at	RNFT1	ring finger protein, transmembrane 1	-3.131	3.99E-07
Bt.16125.3.S1_at	RNFT1	ring finger protein, transmembrane 1	-3.455	1.21E-03
Bt.120.2.S1_at	ROCK1	Rho-associated, coiled-coil containing protein kinase 1	3.321	8.10E-04

Bt.120.1.S1_at	ROCK2	Rho-associated, coiled-coil containing protein kinase 2	3.815	2.33E-04
Bt.20216.2.S1_at	RPIA	ribose 5-phosphate isomerase A	4.748	7.04E-09
Bt.20216.3.S1_a_at	RPIA	ribose 5-phosphate isomerase A	4.229	3.30E-08
Bt.18696.1.S1_at	RPS6KA4	ribosomal protein S6 kinase, 90kDa, polypeptide 4	-3.339	1.71E-06
Bt.24467.1.S1_at	RSAD2	radical S-adenosyl methionine domain containing 2	-3.147	6.37E-03
Bt.24455.1.S1_at	RSPH10B	radial spoke head 10 homolog B (Chlamydomonas)	-3.163	2.39E-07
Bt.21401.1.S1_at	RSPH9	Radial spoke head 9 homolog (Chlamydomonas)	-3.652	2.94E-08
Bt.24212.1.S1_at	RSPO3	R-spondin 3 homolog (Xenopus laevis)	7.086	9.31E-06
Bt.22064.2.S1_at	RSRC2	arginine/serine-rich coiled-coil 2	7.130	2.19E-05
Bt.3750.1.S1_at	S100A11	S100 calcium binding protein A11	3.317	5.86E-05
Bt.7594.1.S1_at	SAT1	spermidine/spermine N1-acetyltransferase 1	15.194	1.48E-05
Bt.11651.1.A1_at	SCAND1	SCAN domain containing 1	3.477	1.48E-07
Bt.11666.1.S1_at	SCAP	SREBF chaperone	3.442	3.93E-07

Bt.4798.1.S2_at	SCD	stearoyl-CoA desaturase (delta-9-desaturase)	-5.552	2.20E-04
Bt.5427.1.A1_at	SCG2	secretogranin II	24.384	5.71E-06
Bt.19470.1.A1_at	SCG3	secretogranin III	3.974	2.96E-03
Bt.1580.1.S1_at	SCG5	secretogranin V (7B2 protein)	3.103	1.69E-03
Bt.9562.1.S1_at	SCN5A	sodium channel, voltage-gated, type V, alpha subunit	3.918	3.34E-09
Bt.2494.2.S1_a_at	SDC2	Syndecan 2	8.992	1.62E-06
Bt.11462.1.S1_at	SDC2	syndecan 2	4.760	3.53E-06
Bt.14031.1.S1_at	SDC4	syndecan 4	3.427	1.05E-05
Bt.2314.1.S1_at	SELL	selectin L	-3.944	3.11E-06
Bt.4342.1.S1_at	SELP	selectin P	4.445	3.47E-03
Bt.2220.2.A1_a_at	SEPP1	selenoprotein P, plasma, 1	3.732	8.62E-04
Bt.14150.1.S1_at	SEPT4	septin 4	-3.112	8.91E-03
Bt.13073.1.S1_at	SERPINB2	serpin peptidase inhibitor, clade B (ovalbumin),	3.090	8.67E-03

		member 2		
Bt.29678.1.S1_at	SERPINB6	serpin peptidase inhibitor, clade B (ovalbumin), member 6	-4.340	5.98E-07
Bt.20432.1.S1_at	SERPINB8	serpin peptidase inhibitor, clade B (ovalbumin), member 8	7.407	3.83E-09
Bt.5229.1.S1_at	SERPINE1	serpin peptidase inhibitor, clade E (nexin, plasminogen activator inhibitor type	19.299	8.50E-08
Bt.25587.1.A1_at	SERPINI2	serpin peptidase inhibitor, clade I (pancpin), member 2	-3.134	6.99E-07
Bt.5828.1.S1_at	SERTAD1	SERTA domain containing 1	4.275	2.93E-07
Bt.16234.2.S1_at	SFRS18	splicing factor, arginine/serine-rich 18	3.195	5.48E-04
Bt.16123.1.S1_at	SGK1	serum/glucocorticoid regulated kinase 1	31.894	5.32E-06
Bt.16123.2.S1_a_at	SGK1	serum/glucocorticoid regulated kinase 1	27.492	2.01E-05
Bt.28541.1.S1_at	SGOL1	shugoshin-like 1 (S. pombe)	-3.782	4.43E-03
Bt.20680.1.S1_at	SH3BP5	SH3-domain binding protein 5 (BTK-associated)	7.475	2.01E-06
Bt.2215.1.S1_at	SH3GL2	SH3-domain GRB2-like 2	-3.130	7.96E-06

Bt.22362.1.S1_at	SH3KBP1	SH3-domain kinase binding protein 1	7.391	5.34E-05
Bt.9720.1.S1_at	SH3RF1	SH3 domain containing ring finger 1	4.036	1.81E-05
Bt.9863.1.S1_at	SHCBP1	SHC SH2-domain binding protein 1	-3.620	1.77E-02
Bt.20287.1.S1_at	SHF	Src homology 2 domain containing F	3.888	8.40E-07
Bt.22389.1.S1_at	SHISA2	shisa homolog 2 (Xenopus laevis)	3.909	2.77E-03
Bt.26844.1.A1_at	SKA3	spindle and kinetochore associated complex subunit 3	-3.029	3.80E-03
Bt.26844.2.S1_at	SKA3	spindle and kinetochore associated complex subunit 3	-3.877	4.65E-04
Bt.20853.2.S1_at	SKAP1	src kinase associated phosphoprotein 1	-4.455	2.31E-06
Bt.28307.1.S1_at	SLC10A2	solute carrier family 10 (sodium/bile acid cotransporter family), member 2	-7.376	1.93E-06
Bt.24743.1.S1_at	SLC12A8	solute carrier family 12 (potassium/chloride transporters), member 8	-4.047	9.08E-09
Bt.28525.1.S1_at	SLC13A2	solute carrier family 13 (sodium-dependent dicarboxylate transporter), member 2	-3.028	5.81E-06
Bt.15616.1.S1_at	SLC16A3	solute carrier family 16, member 3 (monocarboxylic acid transporter 4)	-4.080	9.31E-04

Bt.26942.1.S1_at	SLC17A5	solute carrier family 17 (anion/sugar transporter), member 5	9.013	4.62E-05
Bt.19.2.A1_at	SLC24A1	solute carrier family 24 (sodium/potassium/calcium exchanger), member 1	-3.982	3.58E-07
Bt.10808.1.S1_at	SLC25A17	solute carrier family 25 (mitochondrial carrier; peroxisomal membrane protein, 3	3.407	4.30E-08
Bt.20016.1.S1_at	SLC25A29	solute carrier family 25, member 29	4.229	1.39E-04
Bt.9855.1.S1_at	SLC25A34	solute carrier family 25, member 34	-3.136	2.41E-06
Bt.1828.1.A1_at	SLC27A3	solute carrier family 27 (fatty acid transporter), member 3	-5.480	2.53E-04
Bt.9598.1.S1_at	SLC37A2	solute carrier family 37 (glycerol-3-phosphate transporter), member 2	-3.045	9.47E-07
Bt.25744.1.A1_at	SLC38A11	solute carrier family 38, member 11	-3.019	3.96E-08
Bt.22697.1.A1_at	SLC39A8	solute carrier family 39 (zinc transporter), member 8	5.170	4.01E-04
Bt.22759.1.S1_at	SLC40A1	solute carrier family 40 (iron-regulated transporter), member 1	7.245	2.79E-04
Bt.29774.1.A1_at	SLC4A2	solute carrier family 4, anion exchanger, member 2 (erythrocyte membrane protein	-4.521	1.65E-07

Bt.10949.1.S1_at	SLC7A1	Solute carrier family 7 (cationic amino acid transporter, y+ system), member 1	-3.491	4.28E-05
Bt.29772.1.A1_at	SLC8A1	solute carrier family 8 (sodium/calcium exchanger), member 1	-3.389	2.54E-06
Bt.12849.1.S1_at	SLCO2B1	solute carrier organic anion transporter family, member 2B1	-5.086	4.99E-08
Bt.17460.1.A1_at	SLITRK2	SLIT and NTRK-like family, member 2	4.282	8.99E-04
Bt.3523.1.A1_at	SMAD3	SMAD family member 3	4.308	6.17E-08
Bt.6544.1.S1_at	SMAGP	small cell adhesion glycoprotein	-3.517	1.22E-08
Bt.15979.1.S1_at	SMN1	survival motor neuron	-5.374	1.18E-07
Bt.14326.1.S1_at	SMS	spermine synthase	3.831	2.13E-08
Bt.4565.1.S1_at	SNAI2	snail homolog 2 (Drosophila)	5.732	3.72E-06
Bt.9947.1.S1_at	SNN	Stannin	3.181	4.80E-06
Bt.7486.1.S1_at	SNRNP200	small nuclear ribonucleoprotein 200kDa (U5)	3.176	7.64E-07
Bt.13371.1.S1_at	SNTB2	syntrophin, beta 2 (dystrophin-associated protein A1, 59kDa, basic component 2)	3.963	2.04E-06

Bt.5507.2.S1_at	SNX9	sorting nexin 9	3.014	4.30E-06
Bt.7839.1.A1_at	SON	SON DNA binding protein	4.137	2.12E-04
Bt.11587.3.A1_a_at	SPAG5	sperm associated antigen 5	-3.182	2.00E-02
Bt.6768.1.S1_at	SPARC	secreted protein, acidic, cysteine-rich (osteonectin)	3.606	7.07E-05
Bt.14116.1.A1_at	SPATS2L	spermatogenesis associated, serine-rich 2-like	3.878	7.38E-04
Bt.8721.1.A1_at	SPEF1	sperm flagellar 1	-5.264	8.90E-08
Bt.2520.1.S1_at	SPOCK2	sparc/osteonectin, cwcv and kazal-like domains proteoglycan (testican) 2	23.750	3.92E-07
Bt.18280.1.S1_at	SPON2	spondin 2, extracellular matrix protein	3.488	1.63E-04
Bt.2632.1.S1_at	SPP1	secreted phosphoprotein 1	14.111	1.01E-03
Bt.25648.1.A1_at	SPPL2B	signal peptide peptidase-like 2B	-3.207	7.44E-07
Bt.16504.1.A1_at	SR140	U2-associated SR140 protein	-3.049	1.18E-05
Bt.16048.1.S1_at	SRGN	serglycin	-4.032	2.02E-03

Bt.2219.1.S1_at	SRP14	signal recognition particle 14kDa (homologous Alu RNA binding protein)	4.735	1.64E-07
Bt.29787.1.S1_s_at	ST6GAL1	ST6 beta-galactosamide alpha-2,6-sialyltranferase 1	-3.075	6.15E-08
Bt.299.1.S1_at	ST6GAL1	ST6 beta-galactosamide alpha-2,6-sialyltranferase 1	-3.214	5.25E-08
Bt.3547.1.A1_at	STAC	SH3 and cysteine rich domain	-5.618	6.26E-07
Bt.27816.1.S1_at	STAC3	SH3 and cysteine rich domain 3	-6.797	9.50E-07
Bt.4709.1.S1_at	STAR	steroidogenic acute regulatory protein	22.511	5.81E-05
Bt.27269.2.S1_at	STARD3NL	STARD3 N-terminal like	3.731	1.58E-07
Bt.15334.1.S1_at	STAT3	signal transducer and activator of transcription 3 (acute-phase response factor)	4.278	2.22E-06
Bt.26373.1.A1_at	STAU1	Staufen, RNA binding protein, homolog 1 (Drosophila)	4.131	8.39E-08
Bt.10272.1.S1_at	STC1	stanniocalcin 1	49.114	3.34E-09
Bt.16925.1.A1_at	STEAP1	six transmembrane epithelial antigen of the prostate 1	4.031	1.01E-03
Bt.10214.1.S1_at	STK17A	serine/threonine kinase 17a	4.960	1.19E-04

Bt.21548.1.A1_at	STK38L	serine/threonine kinase 38 like	3.099	7.91E-06
Bt.3196.1.S1_at	STRA6	stimulated by retinoic acid gene 6 homolog (mouse)	-3.584	1.48E-03
Bt.3537.1.S1_at	SULT1A1	sulfotransferase family, cytosolic, 1A, phenol-preferring, member 1	3.076	1.34E-04
Bt.4329.1.S1_at	SVIL	supervillin	6.138	3.94E-08
Bt.15774.1.S1_at	SWAP70	SWAP switching B-cell complex 70kDa subunit	11.859	5.10E-09
Bt.8054.2.S1_a_at	SYAP1	synapse associated protein 1	3.109	3.00E-06
Bt.9438.1.S1_at	SYNCRIP	synaptotagmin binding, cytoplasmic RNA interacting protein	3.634	6.17E-04
Bt.10565.1.A1_at	SYNE1	spectrin repeat containing, nuclear envelope 1	7.630	1.40E-06
Bt.27471.1.S1_at	SYNGR3	synaptogyrin 3	-3.299	1.20E-08
Bt.6698.1.S1_at	SYNPO	synaptopodin	-3.570	1.33E-10
Bt.21873.1.S1_at	SYT11	synaptotagmin XI	3.167	3.08E-07
Bt.29029.1.S1_at	TACC2	transforming, acidic coiled-coil containing protein 2	4.789	4.92E-07

Bt.16055.2.S1_a_at	TAGLN	transgelin	10.810	3.22E-06
Bt.23135.1.S1_at	TAGLN2	transgelin 2	4.265	1.71E-03
Bt.4863.1.S1_at	TAGLN3	transgelin 3	-3.443	9.01E-08
Bt.12261.2.S1_a_at	TASP1	taspase, threonine aspartase, 1	-3.172	1.98E-06
Bt.1987.1.S1_at	TAX1BP3	Tax1 (human T-cell leukemia virus type I) binding protein 3	4.796	3.30E-08
Bt.25408.2.S1_at	TBKBP1	TBK1 binding protein 1	-6.189	1.40E-08
Bt.8326.1.A1_at	TCF3	transcription factor 3 (E2A immunoglobulin enhancer binding factors E12/E47)	3.263	5.43E-07
Bt.1303.1.S1_at	TCF7L2	transcription factor 7-like 2 (T-cell specific, HMG-box)	5.841	1.25E-07
Bt.3704.2.S1_a_at	TCN2	transcobalamin II	3.411	1.49E-07
Bt.16875.1.A1_at	TCP11L2	t-complex 11 (mouse)-like 2	3.159	5.94E-03
Bt.4289.1.S1_at	TCRA	T cell receptor, alpha	3.011	1.85E-07
Bt.13003.6.A1_at	TCRA	T cell receptor, alpha	-3.348	1.36E-06

Bt.13003.4.A1_at	TCRA	T cell receptor, alpha	-3.357	2.25E-07
Bt.13003.15.A1_x_at	TCRA	T cell receptor, alpha	-3.996	3.85E-07
Bt.13003.10.A1_at	TCRA	T cell receptor, alpha	-6.411	1.02E-06
Bt.13003.15.A1_at	TCRA	T cell receptor, alpha	-9.420	1.95E-08
Bt.25103.1.S1_at	TDRD7	tudor domain containing 7	4.334	2.81E-07
Bt.2750.1.S1_at	TFAP2A	transcription factor AP-2 alpha (activating enhancer binding protein 2 alpha)	-3.115	1.83E-06
Bt.24154.1.A1_at	TFDP2	transcription factor Dp-2 (E2F dimerization partner 2)	3.133	1.73E-07
Bt.24864.2.A1_at	TFPI	tissue factor pathway inhibitor (lipoprotein-associated coagulation inhibitor)	-5.086	2.44E-09
Bt.1789.1.S1_at	TGFBR2	transforming growth factor, beta receptor II (70/80kDa)	4.984	2.67E-06
Bt.20778.3.A1_at	TGIF1	TGFB-induced factor homeobox 1	3.399	1.15E-04
Bt.5522.1.S1_at	THBS2	thrombospondin 2	23.905	9.13E-08
Bt.632.1.S1_s_at	TIMP1	TIMP metallopeptidase inhibitor 1	68.933	2.27E-07

Bt.632.1.S1_at	TIMP1	TIMP metallopeptidase inhibitor 1	17.587	2.78E-06
Bt.435.1.S1_at	TIMP2	TIMP metallopeptidase inhibitor 2	3.267	1.04E-03
Bt.20601.1.A1_at	TJP2	tight junction protein 2 (zona occludens 2)	4.859	1.37E-06
Bt.20601.2.S1_at	TJP2	tight junction protein 2 (zona occludens 2)	4.381	1.40E-08
Bt.29711.1.A1_at	TJP2	tight junction protein 2 (zona occludens 2)	-3.590	5.92E-08
Bt.4560.1.S1_at	TKDP1	trophoblast Kunitz domain protein 1	-3.620	2.30E-07
Bt.3589.2.A1_at	TLE1	transducin-like enhancer of split 1 (E(sp1) homolog, Drosophila)	5.895	6.81E-08
Bt.26586.1.S1_at	TLE1	transducin-like enhancer of split 1 (E(sp1) homolog, Drosophila)	3.135	1.71E-06
Bt.3589.1.S1_at	TLE1	transducin-like enhancer of split 1 (E(sp1) homolog, Drosophila)	-4.502	2.78E-08
Bt.22710.1.A1_at	TLN1	talin 1	4.058	6.74E-08
Bt.628.1.S1_at	TMED6	transmembrane emp24 protein transport domain containing 6	-3.414	1.23E-06
Bt.10529.2.S1_a_at	TMEM138	transmembrane protein 138	-4.493	8.86E-06

Bt.8222.1.S1_at	TMEM139	transmembrane protein 139	-3.632	1.18E-07
Bt.11176.2.S1_at	TMEM14A	transmembrane protein 14A	5.857	2.24E-08
Bt.15565.1.S1_at	TMEM176A	transmembrane protein 176A	5.447	1.96E-05
Bt.21250.1.S1_a_at	TMEM176B	transmembrane protein 176B	3.123	2.21E-05
Bt.29031.1.A1_at	TMEM20	transmembrane protein 20	-4.717	3.68E-04
Bt.2357.1.S1_at	TMEM20	transmembrane protein 20	-5.097	7.81E-05
Bt.11403.1.A1_at	TMEM216	transmembrane protein 216	-4.039	3.92E-08
Bt.3525.1.S1_at	TMEM49	transmembrane protein 49	5.315	3.30E-08
Bt.7923.1.A1_at	TMIGD2	transmembrane and immunoglobulin domain containing 2	-4.474	1.40E-06
Bt.958.1.A1_at	TNFAIP6	tumor necrosis factor, alpha-induced protein 6	7.130	1.34E-03
Bt.20111.1.S1_at	TNFRSF12A	tumor necrosis factor receptor superfamily, member 12A	10.352	5.64E-07
Bt.3890.1.S1_at	TNFRSF1A	tumor necrosis factor receptor superfamily, member 1A	3.196	2.56E-06

Bt.2656.1.S1_at	TNFRSF25	tumor necrosis factor receptor superfamily, member 25	3.380	5.10E-09
Bt.27361.1.A1_at	TNFRSF6B	tumor necrosis factor receptor superfamily, member 6b, decoy	5.922	7.03E-08
Bt.26998.1.A1_s_at	TNNC1	troponin C type 1 (slow)	-3.166	5.98E-06
Bt.14438.2.A1_a_at	TNPO1	transportin 1	-3.150	4.42E-03
Bt.14438.1.A1_at	TNPO1	transportin 1	-3.663	1.62E-05
Bt.22975.1.S1_at	TNPO1	transportin 1	-5.977	9.59E-06
Bt.17070.1.S1_at	TOP1	topoisomerase (DNA) I	5.081	2.54E-05
Bt.16786.1.A1_at	TOP2A	topoisomerase (DNA) II alpha 170kDa	-3.358	1.47E-02
Bt.20277.1.S1_at	TOP2A	topoisomerase (DNA) II alpha 170kDa	-6.264	1.45E-03
Bt.15703.2.S1_at	TOPORS	topoisomerase I binding, arginine/serine-rich, E3 ubiquitin protein ligase	5.671	3.22E-06
Bt.15703.2.S1_a_at	TOPORS	topoisomerase I binding, arginine/serine-rich, E3 ubiquitin protein ligase	3.526	3.74E-05
Bt.23456.1.A1_s_at	TP53INP1	tumor protein p53 inducible nuclear protein 1	8.917	4.72E-07

Bt.28741.1.A1_at	TPX2	TPX2, microtubule-associated, homolog (Xenopus laevis)	-3.321	8.79E-03
Bt.28741.2.S1_at	TPX2	TPX2, microtubule-associated, homolog (Xenopus laevis)	-3.783	2.64E-03
Bt.19823.1.A1_at	TRAM2	translocation associated membrane protein 2	-3.553	2.61E-06
Bt.20300.1.A1_at	TRANK1	tetratricopeptide repeat and ankyrin repeat containing 1	3.623	1.11E-03
Bt.29764.1.S1_at	TRB@	T cell receptor, beta cluster	-4.202	9.60E-08
Bt.29693.1.A1_at	TRGV7-1	T cell receptor gamma variable 7-1	-3.814	3.84E-06
Bt.15828.2.S1_at	TRIB1	tribbles homolog 1 (Drosophila)	30.587	3.03E-07
Bt.18822.1.A1_at	TRIB2	tribbles homolog 2 (Drosophila)	-3.994	4.35E-04
Bt.22857.1.S2_at	TRIB2	tribbles homolog 2 (Drosophila)	-4.768	5.10E-04
Bt.22857.1.S1_at	TRIB2	tribbles homolog 2 (Drosophila)	-6.512	9.62E-04
Bt.9660.1.A1_s_at	TRIM8	tripartite motif-containing 8	3.011	5.74E-07
Bt.19750.1.A1_at	TRNAU1AP	TRNA selenocysteine 1 associated protein 1	-3.300	5.06E-06

Bt.22366.1.S1_at	TROAP	trophinin associated protein (tastin)	-3.520	4.59E-04
Bt.19959.1.S1_at	TRPA1	transient receptor potential cation channel, subfamily A, member 1	-6.117	4.94E-08
Bt.6392.1.S1_at	TRPM6	transient receptor potential cation channel, subfamily M, member 6	-3.150	9.39E-07
Bt.4870.1.S1_at	TSC22D1	TSC22 domain family, member 1	3.736	2.06E-05
Bt.28390.1.S1_at	TSC22D3	TSC22 domain family, member 3	3.435	2.32E-04
Bt.475.1.S1_at	TSHB	thyroid stimulating hormone, beta	-3.109	1.11E-07
Bt.3988.1.S1_at	TSPO	translocator protein (18kDa)	5.728	6.37E-05
Bt.7619.1.S1_at	TTYH1	tweety homolog 1 (Drosophila)	-4.812	4.34E-06
Bt.5445.3.A1_at	TUBB6	tubulin, beta 6	5.077	4.59E-04
Bt.729.1.S1_at	TYRO3	TYRO3 protein tyrosine kinase	3.078	1.88E-04
Bt.221.1.S1_at	TYRP1	tyrosinase-related protein 1	-4.309	1.98E-08
Bt.12811.1.S1_a_at	UACA	uveal autoantigen with coiled-coil domains and ankyrin repeats	3.231	1.81E-05

Bt.13063.1.S1_at	UBE2A	ubiquitin-conjugating enzyme E2A (RAD6 homolog)	3.478	1.75E-05
Bt.2725.1.S1_at	UBE2C	ubiquitin-conjugating enzyme E2C	-3.112	2.35E-02
Bt.23071.1.A1_at	UBN2	ubinuclein 2	-3.600	2.06E-06
Bt.14324.1.A1_at	UGT2B11	UDP glucuronosyltransferase 2 family, polypeptide B11	-5.887	5.16E-08
Bt.28379.1.S1_at	UHRF1	ubiquitin-like with PHD and ring finger domains 1	-3.549	2.44E-03
Bt.337.1.S1_at	UPK1A	uroplakin 1A	-3.080	4.16E-06
Bt.28717.1.S1_at	USF1	upstream transcription factor 1	6.848	1.51E-08
Bt.24505.1.S1_at	USP28	ubiquitin specific peptidase 28	-3.772	1.27E-08
Bt.21924.1.A1_at	UST	uronyl-2-sulfotransferase	3.083	1.11E-05
Bt.22842.2.S1_at	VAT1	vesicle amine transport protein 1 homolog (T. californica)	6.787	4.84E-07
Bt.20821.1.A1_at	VCL	vinculin	4.308	6.71E-05
Bt.4138.2.S1_at	VEGFA	vascular endothelial growth factor A	-5.814	7.41E-04

Bt.27824.2.S1_at	VGLL1	vestigial like 1 (Drosophila)	-3.795	3.53E-09
Bt.28243.1.S1_a_at	VNN1	vanin 1	15.097	2.18E-03
Bt.28243.2.S1_at	VNN1	vanin 1	9.277	7.30E-04
Bt.21277.1.A1_at	VPS13B	Vacuolar protein sorting 13 homolog B (yeast)	4.127	1.13E-06
Bt.25610.1.A1_at	VPS52	vacuolar protein sorting 52 homolog (S. cerevisiae)	-3.243	1.13E-06
Bt.5555.1.S1_at	VSX1	visual system homeobox 1	-3.979	1.28E-07
Bt.28699.1.S1_at	WASL	Wiskott-Aldrich syndrome-like	4.290	3.88E-07
Bt.26722.1.A1_a_at	WDFY4	WDFY family member 4	11.084	6.49E-08
Bt.216.1.S1_at	WDR44	WD repeat domain 44	5.013	1.84E-07
Bt.4534.3.S1_at	WDR46	WD repeat domain 46	-3.253	2.32E-07
Bt.20187.2.S1_at	WDR76	WD repeat domain 76	-4.394	1.20E-08
Bt.18127.1.A1_at	WDR87	WD repeat domain 87	-3.268	2.27E-06
Bt.24284.1.A1_at	WHSC1L1	Wolf-Hirschhorn syndrome candidate 1-like 1	4.519	3.26E-04

Bt.8144.1.S1_at	XCL2	chemokine (C motif) ligand 2	16.748	3.03E-04
Bt.7568.1.S1_at	YBX2	Y box binding protein 2	-4.936	4.63E-07
Bt.5748.1.S1_at	YPEL5	Yippee-like 5 (Drosophila)	3.875	3.88E-07
Bt.19746.2.A1_at	ZBTB4	zinc finger and BTB domain containing 4	-3.067	3.01E-05
Bt.29569.2.S1_at	ZC3H8	zinc finger CCCH-type containing 8	3.185	4.58E-08
Bt.29569.1.A1_at	ZC3H8	zinc finger CCCH-type containing 8	3.162	7.84E-06
Bt.5998.1.S1_at	ZDHHC23	Zinc finger, DHHC-type containing 23	4.695	6.09E-08
Bt.9629.1.S1_at	ZFP36L1	zinc finger protein 36, C3H type-like 1	3.942	7.37E-06
Bt.9629.2.S1_at	ZFP36L1	zinc finger protein 36, C3H type-like 1	3.518	2.42E-05
Bt.17771.2.A1_at	ZFYVE1	zinc finger, FYVE domain containing 1	3.313	7.96E-06
Bt.4755.1.S1_at	ZMIZ2	zinc finger, MIZ-type containing 2	3.206	7.63E-08
Bt.21669.3.S1_at	ZNF274	Zinc finger protein 274	-3.005	6.03E-07
Bt.23631.1.S1_at	ZNF281	zinc finger protein 281	3.840	9.87E-08

Bt.27394.1.A1_at	ZNF292	zinc finger protein 292	5.671	1.14E-04
Bt.17868.1.A1_at	ZNF317	zinc finger protein 317	4.221	1.03E-05
Bt.25611.1.A1_at	ZNF385A	zinc finger protein 385A	-4.366	1.46E-07
Bt.8699.1.S1_at	ZNF385A	zinc finger protein 385A	-4.548	1.39E-07
Bt.22489.2.A1_at	ZNF398	Zinc finger protein 398	4.152	1.67E-07
Bt.26259.1.A1_at	ZNF462	zinc finger protein 462	7.544	1.65E-07
Bt.20242.1.S1_at	ZNF521	zinc finger protein 521	4.614	6.08E-07
Bt.18479.1.A1_at	ZNF608	Zinc finger protein 608	4.891	6.00E-05
Bt.7032.1.S1_at	ZNHIT3	zinc finger, HIT-type containing 3	3.123	6.11E-08
Bt.19978.1.A1_at	---	---	80.090	1.52E-06
Bt.20605.1.S1_at	---	---	46.252	2.52E-06
Bt.3100.1.A1_at	---	---	38.329	9.74E-08
Bt.17034.1.A1_at	---	---	28.466	1.42E-05

Bt.13289.2.S1_at	---	---	26.803	6.54E-06
Bt.9841.1.S1_at	---	---	23.013	1.39E-06
Bt.13203.1.S1_at	---	---	18.476	6.56E-06
Bt.1929.1.S1_at	---	---	17.612	1.58E-07
Bt.11001.1.S1_at	---	---	15.133	1.41E-08
Bt.4374.1.S1_at	---	---	14.947	4.39E-06
Bt.21929.1.S1_at	---	---	14.873	3.48E-07
Bt.2234.1.S1_at	---	---	13.732	1.72E-07
Bt.10092.1.S1_at	---	---	12.727	4.91E-05
Bt.20591.2.A1_at	---	---	12.524	2.01E-06
Bt.15290.1.A1_at	---	---	12.463	9.65E-09
Bt.12440.1.A1_at	---	---	11.646	2.63E-06
Bt.21866.1.S1_at	---	---	11.600	4.72E-06

Bt.7128.1.S1_at	---	---	10.796	5.57E-08
Bt.22349.2.S1_at	---	---	10.558	4.48E-05
Bt.28569.1.S1_at	---	---	10.000	2.13E-04
Bt.20605.2.S1_at	---	---	9.994	2.50E-04
Bt.24695.1.S1_at	---	---	9.733	5.20E-08
Bt.13306.1.S1_at	---	---	9.528	4.95E-07
Bt.5682.1.S1_at	---	---	9.507	7.65E-08
Bt.15968.1.S1_at	---	---	9.206	1.78E-05
Bt.6312.1.S1_at	---	---	9.184	5.26E-08
Bt.7467.1.S1_at	---	---	8.903	3.98E-05
Bt.11778.1.A1_at	---	---	8.730	2.23E-05
Bt.9966.1.S1_at	---	---	8.288	3.94E-05
Bt.28642.1.A1_at	---	---	7.941	5.52E-07

Bt.26316.1.A1_at	---	---	7.615	8.48E-06
Bt.22132.1.S1_at	---	---	7.510	2.52E-07
Bt.12453.1.A1_a_at	---	---	7.488	2.05E-04
Bt.20507.1.S1_at	---	---	7.132	1.91E-03
Bt.13985.1.S1_at	---	---	6.998	2.81E-07
Bt.20308.1.S1_at	---	---	6.936	7.94E-07
Bt.10994.1.S1_at	---	---	6.862	1.64E-05
Bt.26926.1.S1_at	---	---	6.811	1.64E-06
Bt.25445.1.A1_at	---	---	6.624	3.05E-08
Bt.28314.1.S1_at	---	---	6.510	2.23E-07
Bt.18914.1.S1_at	---	---	6.488	4.84E-04
Bt.20639.1.A1_at	---	---	6.427	4.94E-06
Bt.3051.1.S1_at	---	---	6.425	1.33E-07

Bt.18420.2.A1_at	---	---	6.378	4.30E-06
Bt.9844.1.S1_at	---	---	6.276	8.13E-08
Bt.17288.1.A1_at	---	---	6.232	3.69E-04
Bt.9781.1.S1_at	---	---	6.167	1.53E-08
Bt.27019.1.S1_at	---	---	6.107	4.86E-06
Bt.17713.2.A1_s_at	---	---	6.105	5.56E-06
Bt.24600.1.S1_at	---	---	6.055	2.17E-07
Bt.16181.1.A1_at	---	---	6.028	4.90E-07
Bt.13829.1.S1_at	---	---	5.991	7.50E-03
Bt.22498.1.A1_at	---	---	5.907	1.14E-05
Bt.12907.2.S1_a_at	---	---	5.863	1.25E-06
Bt.21203.1.S1_at	---	---	5.822	2.17E-07
Bt.16063.1.A1_at	---	---	5.704	1.23E-07

Bt.20501.1.S1_at	---	---	5.676	1.98E-05
Bt.28193.1.S1_at	---	---	5.663	3.79E-05
Bt.12453.1.A1_at	---	---	5.455	1.08E-03
Bt.22349.1.A1_at	---	---	5.443	2.61E-04
Bt.12466.1.A1_at	---	---	5.340	1.60E-06
Bt.15798.1.S1_at	---	---	5.335	7.44E-05
Bt.18843.1.A1_at	---	---	5.286	1.24E-05
Bt.13289.1.S1_at	---	---	5.243	1.48E-03
Bt.11323.1.A1_at	---	---	5.234	1.04E-05
Bt.668.1.S1_at	---	---	5.221	5.65E-06
Bt.22459.1.S1_at	---	---	5.214	2.18E-04
Bt.27281.1.S1_at	---	---	5.176	6.39E-06
Bt.12294.1.S1_at	---	---	5.122	3.09E-05

Bt.21018.1.S1_at	---	---	5.105	4.76E-08
Bt.24145.1.A1_at	---	---	5.098	4.80E-04
Bt.13489.1.S1_at	---	---	5.072	5.21E-08
Bt.22413.1.A1_at	---	---	5.071	2.55E-06
Bt.22443.1.A1_at	---	---	5.055	4.60E-05
Bt.5851.1.A1_at	---	---	5.032	1.31E-05
Bt.1309.1.A1_at	---	---	4.972	3.64E-06
Bt.22797.1.A1_at	---	---	4.907	1.92E-05
Bt.12142.1.S1_at	---	---	4.879	1.66E-07
Bt.20696.1.A1_at	---	---	4.869	6.39E-08
Bt.21668.1.S1_at	---	---	4.864	9.53E-08
Bt.24417.1.A1_at	---	---	4.843	5.34E-07
Bt.17530.1.A1_at	---	---	4.663	1.22E-06

Bt.25364.1.A1_at	---	---	4.623	4.89E-05
Bt.12112.1.S1_at	---	---	4.575	4.06E-07
Bt.4726.2.S1_at	---	---	4.538	1.82E-05
Bt.8718.1.S1_at	---	---	4.506	1.24E-08
Bt.19382.1.S1_at	---	---	4.400	5.10E-06
Bt.7647.1.A1_at	---	---	4.386	5.81E-07
Bt.2318.1.A1_at	---	---	4.381	9.50E-07
Bt.6121.1.A1_at	---	---	4.373	1.68E-07
Bt.18643.1.A1_at	---	---	4.370	2.70E-06
Bt.18581.1.A1_at	---	---	4.346	2.26E-04
Bt.19809.2.A1_at	---	---	4.313	7.93E-07
Bt.27140.1.S1_at	---	---	4.311	1.41E-04
Bt.5788.1.S1_at	---	---	4.310	2.14E-04

Bt.2147.1.S1_at	---	---	4.281	8.23E-07
Bt.28702.1.S1_at	---	---	4.271	9.68E-06
Bt.24279.1.S1_s_at	---	---	4.229	9.96E-06
Bt.17161.1.S1_at	---	---	4.224	2.67E-05
Bt.21284.1.A1_at	---	---	4.220	8.11E-05
Bt.21813.1.S1_at	---	---	4.207	1.24E-04
Bt.24936.1.A1_at	---	---	4.166	1.77E-04
Bt.18409.1.A1_at	---	---	4.128	1.94E-02
Bt.10354.1.S1_at	---	---	4.121	2.10E-04
Bt.24536.1.S1_at	---	---	4.112	1.91E-06
Bt.20591.1.S1_at	---	---	4.081	5.02E-06
Bt.16628.2.A1_at	---	---	4.074	2.19E-02
Bt.16063.2.S1_at	---	---	4.021	5.66E-07

Bt.1268.1.A1_at	---	---	3.991	2.49E-05
Bt.1343.2.A1_at	---	---	3.990	2.22E-07
Bt.15442.1.A1_at	---	---	3.974	6.28E-04
Bt.24208.1.S1_at	---	---	3.972	1.67E-09
Bt.10388.2.S1_at	---	---	3.931	2.82E-07
Bt.19510.2.A1_at	---	---	3.848	6.15E-07
Bt.7553.1.S1_at	---	---	3.826	1.24E-04
Bt.8782.1.S1_at	---	---	3.821	1.10E-03
Bt.23148.1.S1_at	---	---	3.792	3.22E-04
Bt.18227.1.S1_at	---	---	3.781	1.74E-07
Bt.15577.1.A1_at	---	---	3.768	7.69E-08
Bt.6117.1.S1_at	---	---	3.751	1.13E-07
Bt.7307.2.A1_a_at	---	---	3.741	9.31E-06

Bt.27263.1.A1_at	---	---	3.732	1.90E-06
Bt.21736.1.A1_at	---	---	3.730	4.89E-04
Bt.28267.1.S1_at	---	---	3.729	4.38E-09
Bt.1775.1.A1_at	---	---	3.725	2.70E-06
Bt.27814.1.S1_at	---	---	3.721	7.34E-05
Bt.24919.1.S1_at	---	---	3.705	2.63E-04
Bt.24324.1.A1_at	---	---	3.700	1.33E-04
Bt.24446.1.A1_at	---	---	3.675	4.44E-05
Bt.20089.1.A1_s_at	---	---	3.644	7.09E-07
Bt.19100.3.A1_at	---	---	3.608	1.61E-06
Bt.24247.1.S1_at	---	---	3.601	2.53E-05
Bt.25147.1.A1_at	---	---	3.600	2.04E-05
Bt.20226.1.A1_at	---	---	3.596	1.30E-04

Bt.6890.1.S1_at	---	---	3.583	1.96E-05
Bt.2123.1.S1_at	---	---	3.568	3.16E-05
Bt.1807.1.A1_at	---	---	3.557	7.40E-05
Bt.9907.1.S1_at	---	---	3.556	7.23E-05
Bt.18184.1.A1_at	---	---	3.536	1.44E-06
Bt.5705.1.S1_at	---	---	3.501	3.90E-07
Bt.17584.1.S1_at	---	---	3.455	4.90E-05
Bt.10922.1.A1_at	---	---	3.443	2.14E-04
Bt.24222.1.S1_at	---	---	3.440	2.80E-04
Bt.3405.1.S1_at	---	---	3.432	7.31E-03
Bt.15887.2.S1_a_at	---	---	3.432	3.63E-08
Bt.16764.1.A1_at	---	---	3.430	4.00E-06
Bt.20773.1.S1_at	---	---	3.428	1.10E-07

Bt.11168.1.S1_at	---	---	3.406	5.74E-04
Bt.23227.1.S1_at	---	---	3.404	4.43E-07
Bt.9037.1.S1_at	---	---	3.390	8.17E-04
Bt.10623.1.S1_at	---	---	3.375	6.20E-08
Bt.22470.1.S1_at	---	---	3.373	5.86E-05
Bt.20966.1.A1_at	---	---	3.332	4.24E-05
Bt.17497.1.S1_at	---	---	3.325	4.59E-06
Bt.26415.1.A1_at	---	---	3.318	3.21E-05
Bt.27639.2.A1_at	---	---	3.311	2.03E-03
Bt.23576.1.A1_at	---	---	3.310	6.39E-07
Bt.16311.2.S1_at	---	---	3.308	2.71E-05
Bt.19602.1.A1_at	---	---	3.300	2.57E-07
Bt.24260.1.S1_at	---	---	3.293	1.52E-06

Bt.2369.1.S1_at	---	---	3.286	9.69E-07
Bt.17330.1.A1_at	---	---	3.284	6.75E-06
Bt.24538.1.S1_at	---	---	3.276	4.24E-06
Bt.27933.1.A1_at	---	---	3.276	4.57E-05
Bt.24316.1.A1_at	---	---	3.272	2.74E-04
Bt.13744.1.A1_at	---	---	3.272	2.66E-05
Bt.4484.1.S1_at	---	---	3.265	3.54E-06
Bt.5732.1.S1_at	---	---	3.254	2.71E-05
Bt.13755.1.S1_at	---	---	3.239	4.20E-06
Bt.7459.1.S1_at	---	---	3.227	5.67E-04
Bt.28621.1.S1_at	---	---	3.224	2.54E-05
Bt.5911.2.S1_at	---	---	3.223	3.38E-06
Bt.18576.1.S1_at	---	---	3.218	2.82E-07

Bt.24468.1.S1_at	---	---	3.206	6.02E-06
Bt.24701.1.A1_at	---	---	3.187	2.49E-04
Bt.11974.1.S1_at	---	---	3.176	1.03E-05
Bt.13492.1.S1_at	---	---	3.176	1.61E-04
Bt.19513.1.A1_at	---	---	3.166	2.86E-03
Bt.21288.1.A1_at	---	---	3.166	1.33E-06
Bt.20910.1.S1_at	---	---	3.155	6.64E-07
Bt.20910.2.S1_at	---	---	3.137	1.47E-06
Bt.10777.1.S1_at	---	---	3.133	1.37E-05
Bt.16311.1.A1_at	---	---	3.133	2.53E-05
Bt.11679.1.S1_at	---	---	3.131	6.06E-06
Bt.22452.1.S1_at	---	---	3.118	8.42E-05
Bt.22303.1.S1_at	---	---	3.106	5.56E-03

Bt.24509.1.S1_at	---	---	3.106	1.58E-03
Bt.12381.1.A1_at	---	---	3.102	1.84E-06
Bt.24689.1.S1_at	---	---	3.098	8.10E-07
Bt.19809.1.S1_at	---	---	3.096	1.19E-06
Bt.20506.1.S1_at	---	---	3.094	1.61E-06
Bt.5736.1.S1_at	---	---	3.093	3.05E-05
Bt.8260.1.A1_at	---	---	3.086	2.14E-04
Bt.14249.1.A1_at	---	---	3.083	3.03E-07
Bt.15623.1.S1_at	---	---	3.073	1.58E-04
Bt.26915.1.S1_at	---	---	3.068	1.08E-05
Bt.26433.1.A1_at	---	---	3.067	9.48E-05
Bt.8443.1.S1_at	---	---	3.066	1.87E-06
Bt.22362.2.S1_at	---	---	3.056	5.17E-04

Bt.17387.1.A1_at	---	---	3.055	2.12E-04
Bt.20404.1.S1_at	---	---	3.053	8.46E-03
Bt.2600.1.A1_at	---	---	3.049	2.19E-07
Bt.11500.1.A1_at	---	---	3.044	1.59E-07
Bt.16687.1.A1_at	---	---	3.043	4.81E-03
Bt.6530.1.S1_at	---	---	3.039	1.59E-04
Bt.9538.1.S1_at	---	---	3.031	1.19E-03
Bt.2194.1.S1_at	---	---	3.028	1.80E-07
Bt.25255.1.A1_at	---	---	3.020	4.15E-05
Bt.26144.1.A1_at	---	---	-3.004	1.57E-07
Bt.8633.1.A1_at	---	---	-3.008	2.96E-02
Bt.29550.1.A1_at	---	---	-3.010	1.45E-05
Bt.25976.1.A1_at	---	---	-3.015	2.91E-07

Bt.27480.1.A1_at	---	---	-3.017	1.61E-05
Bt.16509.1.A1_at	---	---	-3.025	2.91E-06
Bt.29012.1.A1_at	---	---	-3.030	3.22E-05
Bt.19741.1.A1_at	---	---	-3.032	6.15E-07
Bt.29037.1.A1_at	---	---	-3.033	3.77E-07
Bt.25765.1.A1_at	---	---	-3.035	7.17E-08
Bt.17822.1.A1_at	---	---	-3.040	2.13E-08
Bt.19350.1.A1_at	---	---	-3.043	5.22E-08
Bt.28165.1.A1_at	---	---	-3.045	6.29E-07
Bt.19361.1.A1_at	---	---	-3.058	1.48E-05
Bt.28880.1.A1_at	---	---	-3.058	1.23E-07
Bt.16100.2.S1_at	---	---	-3.059	2.11E-03
Bt.2738.1.A1_at	---	---	-3.064	1.80E-08

Bt.25808.1.A1_at	---	---	-3.069	5.88E-05
Bt.27183.1.S1_at	---	---	-3.069	1.71E-06
Bt.29722.1.S1_at	---	---	-3.071	1.25E-06
Bt.25387.1.A1_at	---	---	-3.073	3.95E-05
Bt.27490.1.A1_at	---	---	-3.077	3.58E-07
Bt.2229.1.S1_at	---	---	-3.082	1.24E-07
Bt.17797.1.A1_at	---	---	-3.082	1.30E-05
Bt.6800.1.A1_at	---	---	-3.084	4.90E-04
Bt.16621.1.A1_at	---	---	-3.084	3.90E-08
Bt.29685.1.A1_at	---	---	-3.088	8.46E-07
Bt.10441.1.A1_at	---	---	-3.095	6.05E-06
Bt.4033.1.S1_at	---	---	-3.098	2.41E-06
Bt.16669.1.A1_at	---	---	-3.107	8.33E-07

Bt.2112.1.S1_at	---	---	-3.113	7.13E-03
Bt.10785.1.A1_at	---	---	-3.113	2.58E-04
Bt.11730.1.A1_at	---	---	-3.118	3.83E-06
Bt.18732.2.A1_at	---	---	-3.120	3.76E-07
Bt.28564.1.S1_at	---	---	-3.120	1.32E-06
Bt.23997.1.A1_at	---	---	-3.121	2.67E-07
AFFX-Bt-AF323980-1_at	---	---	-3.124	1.13E-05
Bt.20837.1.S1_at	---	---	-3.125	1.58E-07
Bt.18523.1.A1_at	---	---	-3.130	1.05E-05
Bt.29776.1.S1_at	---	---	-3.131	4.66E-06
Bt.17959.1.A1_at	---	---	-3.132	2.16E-06
Bt.26954.1.S1_at	---	---	-3.135	2.90E-08

Bt.1476.1.S1_at	---	---	-3.139	8.48E-06
Bt.22146.2.S1_at	---	---	-3.143	3.12E-06
Bt.28152.1.S1_at	---	---	-3.148	6.92E-07
Bt.20295.1.A1_at	---	---	-3.148	4.35E-03
Bt.25549.1.A1_at	---	---	-3.158	2.13E-05
Bt.28708.1.S1_at	---	---	-3.161	3.22E-08
Bt.29729.1.A1_at	---	---	-3.161	1.32E-07
Bt.19214.1.S1_at	---	---	-3.162	5.30E-07
Bt.29632.1.A1_at	---	---	-3.165	3.88E-07
Bt.28848.1.A1_at	---	---	-3.168	8.04E-07
Bt.1571.1.S1_at	---	---	-3.169	5.81E-06
Bt.16852.1.A1_at	---	---	-3.176	3.34E-09
Bt.17103.1.A1_at	---	---	-3.183	6.74E-05

Bt.28933.1.A1_at	---	---	-3.186	2.77E-06
Bt.27768.1.A1_at	---	---	-3.186	1.46E-05
Bt.25678.1.A1_at	---	---	-3.187	6.85E-07
Bt.5882.1.S1_at	---	---	-3.189	3.85E-06
Bt.17568.1.A1_at	---	---	-3.190	2.66E-08
Bt.5712.1.S1_at	---	---	-3.190	2.90E-07
Bt.9722.1.S1_at	---	---	-3.192	1.40E-06
Bt.29811.1.A1_at	---	---	-3.195	5.99E-07
Bt.25987.1.A1_at	---	---	-3.199	1.22E-07
Bt.26078.1.A1_at	---	---	-3.203	2.27E-07
Bt.18483.1.A1_at	---	---	-3.206	6.09E-07
Bt.15311.1.A1_at	---	---	-3.208	6.15E-08
Bt.24000.1.A1_at	---	---	-3.216	4.98E-06

Bt.16839.2.A1_at	---	---	-3.220	4.08E-09
Bt.13003.2.S1_at	---	---	-3.222	1.11E-06
Bt.9822.1.S1_at	---	---	-3.231	1.44E-05
Bt.27667.1.A1_at	---	---	-3.237	2.01E-07
Bt.29002.1.A1_at	---	---	-3.237	1.27E-08
Bt.13522.1.S1_at	---	---	-3.239	2.16E-06
Bt.17375.1.A1_at	---	---	-3.243	6.09E-07
Bt.19455.1.A1_at	---	---	-3.247	9.04E-07
Bt.12554.1.S1_at	---	---	-3.248	3.37E-07
Bt.25534.1.A1_at	---	---	-3.248	3.90E-07
Bt.28803.1.S1_at	---	---	-3.249	1.46E-06
Bt.24798.1.A1_at	---	---	-3.250	5.46E-08
Bt.16192.1.A1_at	---	---	-3.251	5.10E-09

AFFX-Bt-K01486-1_at	---	---	-3.256	2.07E-07
Bt.8878.1.S1_at	---	---	-3.261	3.46E-09
Bt.18950.1.A1_at	---	---	-3.268	5.36E-05
Bt.27700.1.A1_at	---	---	-3.270	2.25E-06
Bt.17501.1.A1_at	---	---	-3.279	7.88E-08
Bt.23714.1.A1_at	---	---	-3.281	1.79E-06
Bt.27593.1.A1_at	---	---	-3.285	2.81E-05
Bt.25542.1.A1_at	---	---	-3.287	7.17E-06
Bt.1739.3.S1_at	---	---	-3.293	2.78E-09
Bt.18373.1.A1_at	---	---	-3.310	6.15E-07
Bt.29517.1.A1_at	---	---	-3.314	2.10E-06
Bt.25566.1.A1_at	---	---	-3.319	8.61E-08

Bt.25461.1.A1_at	---	---	-3.320	5.84E-06
Bt.24144.1.A1_at	---	---	-3.322	5.04E-06
Bt.28847.1.S1_at	---	---	-3.332	1.24E-07
Bt.27644.1.A1_at	---	---	-3.335	2.62E-06
Bt.17258.1.A1_at	---	---	-3.340	6.42E-08
Bt.11600.1.A1_at	---	---	-3.342	1.19E-06
Bt.18724.3.A1_at	---	---	-3.343	3.21E-08
Bt.26305.1.A1_at	---	---	-3.346	4.22E-07
Bt.13569.1.A1_at	---	---	-3.347	7.16E-06
Bt.19785.1.A1_at	---	---	-3.349	7.33E-06
Bt.5856.1.S1_at	---	---	-3.364	3.04E-07
Bt.28925.1.A1_at	---	---	-3.365	2.40E-06
Bt.25972.1.A1_at	---	---	-3.365	3.44E-07

Bt.19900.1.A1_at	---	---	-3.369	2.41E-08
Bt.24030.1.A1_at	---	---	-3.370	3.37E-07
Bt.17663.1.A1_at	---	---	-3.377	4.22E-07
Bt.25639.1.A1_at	---	---	-3.379	9.29E-06
Bt.17577.1.A1_at	---	---	-3.382	2.78E-04
Bt.27209.1.A1_at	---	---	-3.382	4.41E-08
Bt.29333.1.A1_at	---	---	-3.386	1.22E-08
Bt.29699.1.A1_at	---	---	-3.388	7.86E-08
Bt.26251.1.A1_at	---	---	-3.402	2.07E-06
Bt.26331.1.A1_at	---	---	-3.403	6.68E-08
Bt.18051.1.A1_at	---	---	-3.407	7.76E-08
Bt.19354.1.A1_at	---	---	-3.408	9.27E-05
Bt.5934.1.S1_at	---	---	-3.412	7.94E-03

Bt.1190.1.A1_at	---	---	-3.420	2.71E-03
Bt.12587.1.A1_at	---	---	-3.430	4.36E-06
Bt.5820.1.S1_at	---	---	-3.434	6.17E-08
Bt.17590.1.A1_at	---	---	-3.443	8.93E-07
Bt.29091.1.A1_at	---	---	-3.449	6.32E-07
Bt.29047.1.A1_at	---	---	-3.456	5.81E-07
Bt.1296.1.S1_at	---	---	-3.466	5.79E-03
Bt.16893.1.A1_at	---	---	-3.467	1.24E-07
Bt.28776.1.A1_at	---	---	-3.474	9.37E-08
Bt.17470.1.A1_at	---	---	-3.484	6.76E-05
Bt.9601.1.S1_at	---	---	-3.486	1.16E-04
Bt.8677.1.S1_at	---	---	-3.488	3.51E-07
Bt.2199.1.A1_at	---	---	-3.494	2.94E-09

Bt.20340.1.A1_at	---	---	-3.496	5.54E-08
Bt.19630.1.A1_at	---	---	-3.512	7.74E-07
Bt.18000.1.S1_at	---	---	-3.517	1.20E-07
Bt.26166.1.A1_at	---	---	-3.522	1.24E-07
Bt.7784.1.S1_at	---	---	-3.530	2.74E-05
Bt.29707.1.A1_at	---	---	-3.531	7.47E-06
Bt.27679.1.A1_at	---	---	-3.539	3.37E-07
Bt.29832.1.S1_at	---	---	-3.549	1.15E-05
Bt.18505.1.S1_at	---	---	-3.556	4.30E-07
Bt.13407.1.S1_at	---	---	-3.562	2.13E-08
Bt.23967.1.A1_at	---	---	-3.569	7.85E-08
Bt.16724.1.A1_at	---	---	-3.573	2.44E-09
Bt.19124.1.A1_at	---	---	-3.575	5.40E-09

Bt.10590.1.A1_at	---	---	-3.592	1.19E-07
Bt.29476.1.A1_at	---	---	-3.594	3.96E-06
Bt.29652.1.A1_at	---	---	-3.603	1.39E-07
Bt.29361.1.A1_at	---	---	-3.603	5.51E-07
Bt.18967.1.A1_at	---	---	-3.605	7.47E-06
Bt.28840.1.S1_at	---	---	-3.610	5.45E-07
Bt.15568.1.A1_at	---	---	-3.610	5.35E-05
Bt.8875.1.S1_at	---	---	-3.618	3.95E-08
Bt.13753.1.A1_at	---	---	-3.619	7.39E-05
Bt.8932.1.S1_at	---	---	-3.629	2.03E-07
Bt.26071.2.S1_at	---	---	-3.637	3.51E-06
Bt.15359.1.A1_at	---	---	-3.653	3.59E-07
Bt.29597.1.A1_at	---	---	-3.678	2.33E-08

Bt.20534.1.S1_at	---	---	-3.684	6.17E-08
Bt.18620.1.A1_at	---	---	-3.686	4.13E-07
Bt.19378.1.A1_at	---	---	-3.700	1.27E-08
Bt.26467.1.A1_at	---	---	-3.704	8.50E-08
Bt.11618.1.A1_at	---	---	-3.719	1.73E-05
Bt.21297.1.S1_at	---	---	-3.723	5.06E-07
Bt.29402.1.A1_at	---	---	-3.738	1.19E-07
Bt.26744.1.A1_at	---	---	-3.740	1.49E-06
Bt.6315.1.A1_at	---	---	-3.741	1.18E-06
Bt.6636.1.S1_at	---	---	-3.746	3.95E-08
Bt.16997.1.A1_at	---	---	-3.746	2.18E-06
Bt.23872.1.A1_at	---	---	-3.748	8.14E-07
Bt.28056.1.S1_at	---	---	-3.748	4.60E-07

Bt.6901.1.S1_at	---	---	-3.752	4.92E-07
Bt.9680.1.S1_at	---	---	-3.753	6.11E-08
Bt.28855.1.A1_at	---	---	-3.785	1.11E-03
Bt.25866.1.A1_at	---	---	-3.803	4.63E-06
Bt.17917.2.A1_at	---	---	-3.807	4.46E-06
Bt.28195.1.S1_at	---	---	-3.811	1.21E-07
Bt.19815.1.A1_at	---	---	-3.827	1.38E-07
Bt.28773.1.A1_at	---	---	-3.827	2.13E-08
Bt.2810.1.A1_at	---	---	-3.836	9.56E-05
Bt.28405.1.S1_at	---	---	-3.844	2.81E-07
Bt.29701.1.A1_at	---	---	-3.868	3.97E-08
Bt.6015.1.S1_at	---	---	-3.884	4.30E-08
Bt.17675.1.A1_at	---	---	-3.887	8.50E-07

Bt.15422.1.A1_at	---	---	-3.897	2.19E-06
Bt.17988.1.S1_at	---	---	-3.903	2.29E-06
Bt.20272.1.S1_at	---	---	-3.903	1.63E-04
Bt.25938.1.A1_at	---	---	-3.914	2.22E-07
Bt.15333.1.A1_at	---	---	-3.915	1.01E-05
Bt.21847.1.A1_at	---	---	-3.936	7.78E-06
Bt.25643.1.A1_at	---	---	-3.980	6.46E-08
Bt.15389.1.A1_at	---	---	-3.985	1.42E-06
Bt.18701.1.A1_at	---	---	-3.990	1.58E-06
Bt.29205.1.A1_at	---	---	-3.992	1.90E-08
Bt.24038.1.A1_at	---	---	-3.995	2.06E-08
Bt.25886.1.A1_at	---	---	-4.009	8.93E-07
Bt.26988.1.A1_at	---	---	-4.021	1.18E-08

Bt.19008.1.A1_at	---	---	-4.036	4.36E-07
Bt.24133.1.A1_at	---	---	-4.049	6.92E-06
Bt.29133.1.A1_at	---	---	-4.099	7.12E-08
Bt.26169.1.A1_at	---	---	-4.120	6.81E-09
Bt.19579.1.A1_at	---	---	-4.129	2.44E-08
Bt.18319.1.A1_at	---	---	-4.138	2.21E-06
Bt.23743.1.A1_at	---	---	-4.146	9.99E-08
Bt.18632.1.A1_at	---	---	-4.151	2.71E-05
Bt.29808.1.A1_at	---	---	-4.162	4.64E-07
Bt.29845.1.S1_at	---	---	-4.176	1.85E-05
Bt.27153.1.A1_at	---	---	-4.178	2.50E-07
Bt.28696.1.S1_at	---	---	-4.209	3.58E-07
Bt.27084.1.S1_at	---	---	-4.213	7.76E-05

Bt.19451.1.A1_at	---	---	-4.213	1.12E-07
Bt.29896.1.S1_at	---	---	-4.225	5.78E-09
Bt.23927.1.A1_at	---	---	-4.234	5.43E-07
Bt.19744.1.A1_at	---	---	-4.302	2.06E-08
Bt.29441.1.A1_at	---	---	-4.303	6.11E-07
Bt.23296.1.S1_at	---	---	-4.314	1.96E-07
Bt.23889.1.A1_at	---	---	-4.343	3.92E-05
Bt.20391.2.S1_at	---	---	-4.361	6.51E-08
Bt.27936.2.A1_at	---	---	-4.362	2.75E-08
Bt.28556.2.A1_at	---	---	-4.363	1.67E-07
Bt.16954.1.A1_at	---	---	-4.375	5.45E-07
Bt.29733.1.A1_at	---	---	-4.377	4.17E-07
Bt.9843.1.S1_at	---	---	-4.396	5.52E-08

Bt.20804.1.A1_at	---	---	-4.431	7.63E-08
Bt.15430.1.A1_at	---	---	-4.453	5.49E-07
Bt.29275.1.A1_at	---	---	-4.478	1.22E-08
Bt.25446.1.S1_at	---	---	-4.480	8.10E-09
Bt.25762.1.A1_at	---	---	-4.505	2.20E-10
Bt.29470.1.S1_at	---	---	-4.508	1.40E-08
Bt.5915.1.A1_at	---	---	-4.537	1.69E-07
Bt.26263.1.A1_at	---	---	-4.544	9.31E-07
Bt.12236.1.S1_at	---	---	-4.561	1.82E-03
Bt.12403.2.A1_at	---	---	-4.721	2.38E-06
Bt.29685.1.S1_at	---	---	-4.736	6.06E-08
Bt.18161.1.S1_at	---	---	-4.753	1.00E-06
Bt.6295.1.S1_at	---	---	-4.806	2.80E-08

Bt.29494.1.A1_at	---	---	-4.844	3.38E-06
Bt.28003.1.A1_at	---	---	-4.851	1.88E-08
Bt.16388.1.A1_at	---	---	-4.901	1.94E-08
Bt.6887.1.S1_at	---	---	-4.929	7.38E-07
Bt.643.1.S1_at	---	---	-4.995	7.99E-03
Bt.25360.1.A1_at	---	---	-5.024	4.64E-07
Bt.28936.1.S1_at	---	---	-5.041	2.44E-09
Bt.29775.1.S1_at	---	---	-5.078	2.90E-08
Bt.19499.1.S1_at	---	---	-5.090	2.30E-04
Bt.8796.1.S1_at	---	---	-5.101	1.05E-05
Bt.27507.1.A1_at	---	---	-5.150	4.92E-07
Bt.24367.1.S1_at	---	---	-5.158	1.89E-07
Bt.16976.3.A1_at	---	---	-5.171	6.26E-07

Bt.8875.1.A1_at	---	---	-5.191	8.27E-07
Bt.18617.1.A1_at	---	---	-5.219	8.15E-09
Bt.25196.1.A1_at	---	---	-5.277	5.14E-05
Bt.25293.1.A1_at	---	---	-5.284	3.75E-08
Bt.16404.1.A1_at	---	---	-5.296	2.37E-07
Bt.29155.1.S1_at	---	---	-5.300	2.94E-09
Bt.29502.1.A1_at	---	---	-5.330	6.89E-07
Bt.23849.1.A1_at	---	---	-5.388	1.91E-07
Bt.29593.1.A1_at	---	---	-5.394	4.70E-07
Bt.17921.1.S1_at	---	---	-5.463	9.74E-08
Bt.25558.1.A1_at	---	---	-5.534	2.94E-09
Bt.24759.2.S1_at	---	---	-5.625	1.17E-07
Bt.18351.1.A1_at	---	---	-5.627	4.10E-08

Bt.28079.1.A1_at	---	---	-5.693	1.88E-08
Bt.28430.1.S1_at	---	---	-5.745	1.84E-04
Bt.3475.1.A1_at	---	---	-5.778	2.09E-08
Bt.25740.1.A1_at	---	---	-5.827	1.24E-08
Bt.25356.1.A1_at	---	---	-5.861	1.85E-07
Bt.23823.1.A1_at	---	---	-6.176	1.94E-08
Bt.25717.1.A1_at	---	---	-6.197	1.40E-08
Bt.15455.1.A1_at	---	---	-6.198	4.98E-08
Bt.29794.1.A1_at	---	---	-6.477	2.87E-08
Bt.16333.1.A1_at	---	---	-6.726	1.00E-06
Bt.18448.1.A1_at	---	---	-6.872	1.67E-07
Bt.24426.1.A1_at	---	---	-6.929	7.07E-04
Bt.15390.1.A1_at	---	---	-7.134	2.34E-10

Bt.29752.1.A1_at	---	---	-7.206	3.49E-06
Bt.19123.1.A1_at	---	---	-7.263	8.38E-08
Bt.17853.1.A1_at	---	---	-7.559	1.24E-06
Bt.24940.1.A1_at	---	---	-7.862	1.96E-03
Bt.9122.1.A1_at	---	---	-8.290	8.86E-06
Bt.27687.1.A1_at	---	---	-9.657	2.47E-07
Bt.28767.1.A1_at	---	---	-10.229	1.31E-07
Bt.26322.1.A1_at	---	---	-10.598	4.52E-08
Bt.23779.1.A1_at	---	---	-11.775	3.73E-04
Bt.29726.1.A1_at	---	---	-22.509	1.14E-07
