# Supplementary material for: Transcriptome profiling of granulosa cells from bovine ovarian follicles during atresia
Source: BMC Genomics. 2014 Jan 18;15:40. doi: 10.1186/1471-2164-15-40 (PMC3898078; doi:10.1186/1471-2164-15-40)
Supplement: Additional file 2: Figure S1 — The canonical TGFβ signalling pathway as presented in IPA showing genes which were 4 fold differentially expressed with a FDR P < 0.005 between atretic and healthy follicles from our dataset. Genes which were up regulated in atretic follicles are indicated in red, and those which were down regulated are green, with the degree of fold difference commensurate with the colour intensity. [file 1471-2164-15-40-S2.pdf]

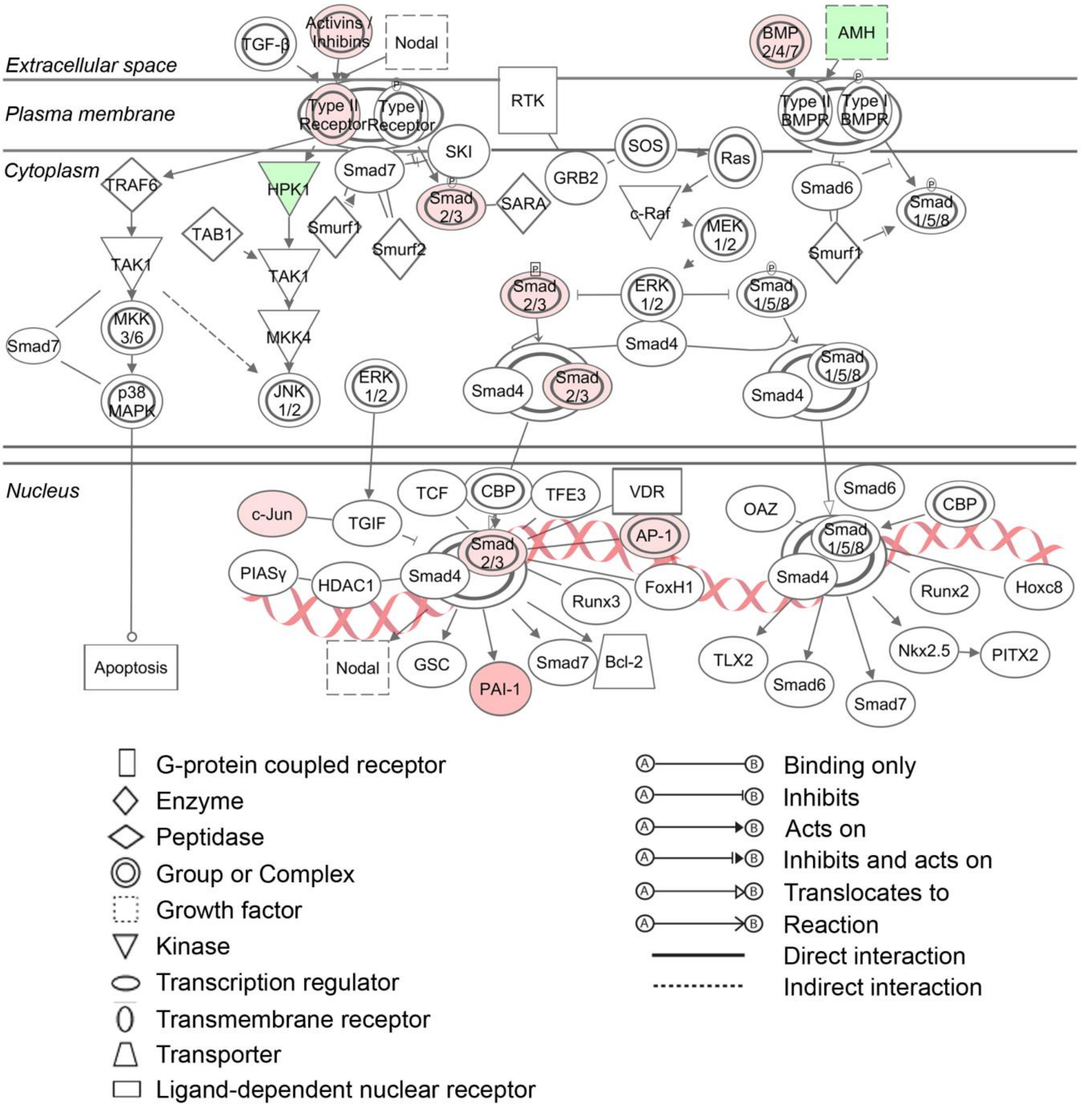

Extracellular space
Plasma membrane
Cytoplasm
Nucleus
TGF-β
Activins / Inhibins
Nodal
BMP 2/4/7
AMH
Type II Receptor
Type I Receptor
RTK
Type II BMPR
Type I BMPR
SKI
SOS
Ras
GRB2
Smad7
TRAF6
HPK1
Smad 2/3
SARA
c-Raf
MEK 1/2
Smad6
Smad 1/5/8
Smurf1
Smurf2
TAB1
TAK1
MKK 3/6
MKK4
ERK 1/2
Smad4
p38 MAPK
JNK 1/2
TCF
CBP
TFE3
VDR
OAZ
c-Jun
TGIF
AP-1
PIASγ
HDAC1
FoxH1
Runx3
Runx2
Hoxc8
Apoptosis
GSC
PAI-1
Bcl-2
TLX2
Nkx2.5
PITX2
G-protein coupled receptor
Enzyme
Peptidase
Group or Complex
Growth factor
Kinase
Transcription regulator
Transmembrane receptor
Transporter
Ligand-dependent nuclear receptor
Binding only
Inhibits
Acts on
Inhibits and acts on
Translocates to
Reaction
Direct interaction
Indirect interaction
