# Supplementary material for: Transcriptome profiling of granulosa cells from bovine ovarian follicles during atresia
Source: BMC Genomics. 2014 Jan 18;15:40. doi: 10.1186/1471-2164-15-40 (PMC3898078; doi:10.1186/1471-2164-15-40)
Supplement: Additional file 3: Table S2 — Genes which were differentially expressed (4 fold, P < 0.005) in atretic follicles with respect to healthy follicles and associated with significant GO terms after enrichment analysis by GOEAST. [file 1471-2164-15-40-S3.pdf]

| GO biological process | *P*-value | Genes |
|---|---|---|
| GO:0001568 blood vessel development | 0.022 | COL3A1, COL1A2, VEGFA, HHEX, IL18, ANXA2, COL18A1, CITED2, CYR61, CTGF, SCG2, CAV1, EPAS1, FGFR2 |
| GO:0007155 cell adhesion | 0.022 | CD9, CDH1, CLDN1, CLDN11, COL18A1, COL3A1, CPXM2, CTGF, CYR61, LAMC2, NID2, OLR1, PCDH7, PDPN, POSTN, SELP, SPP1, THBS2, TNFAIP6, TTYH1, VCL, VNN1 |
| GO:0050794 regulation of cellular process | 0.035 | ACTN1, AGRN, ALB, ANKRD1, ANXA1, APBB1IP, ARHGEF11, ARHGEF2, ARHGEF3, ARPC1B, ATF3, AXL, BAMBI, BMP2, CACNB3, CAPG, CAV1, CD9, CDH1, CDKN1C, CEBPD, CFLAR, CITED2, CLIC4, COL18A1, COL1A2, COL3A1, COL3A1, CRYAB, CTGF, CUL7, CYR61, DKK3, DSTN, ECT2, EGR1, ELMO1, ELTD1, EPAS1, F2RL2, FGFR2, FOXP1, FSHR, FST, GADD45A, GAL, GCLC, GEM, GNG2, GPRC5A, GSN, HHEX, HMBOX1, HOXB2, HSPA1A /// HSPA1B, HTR2A, ID1, ID3, IFI30, IGF2, IGFBP5, IGFBP6, IL18, IL18R1, ING4, IRAK1BP1, JAK1, JUN, KRT18, KRT8, LITAF, LOC510844, LOC515718, LOC534218, LOC538276, LPL, MAP2, MAP4K1, MGC127538, MIA3, MYCBP2, NDP, NFIL3, NNAT, NOS2, NR2F1, NTRK3, OPRM1, PDE4D, PDE6A, PDGFRA, PDK4, PDK4, PDPN, PELI1, PIK3CA, PIM1, PKIB, PLA2G1B, PLAT, PTX3, QSOX1, RAP2C, RASA2, RASAL2, RASGRP4, RBM9, RGS2, RHO, RND3, RSPO3, SAT1, SCG2, SERPINE1, SH3RF1, SPOCK2, SPP1, SRGN, SRP14, STAT3, STK17A, TAX1BP3, TCF7L2, TRIB1, TRIB2, TRIB2, TUBB6, VCL, VEGFA, VNN1, YBX2, ZNF317 |
