# Supplementary figures and images for: Transcriptome profiling of granulosa cells from bovine ovarian follicles during atresia
Source: BMC Genomics. 2014 Jan 18;15:40. doi: 10.1186/1471-2164-15-40 (PMC3898078; doi:10.1186/1471-2164-15-40)

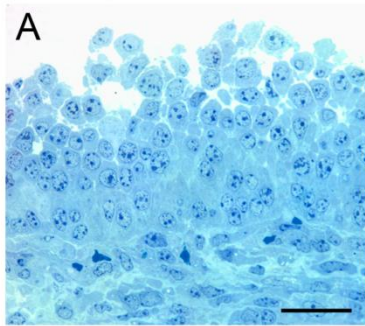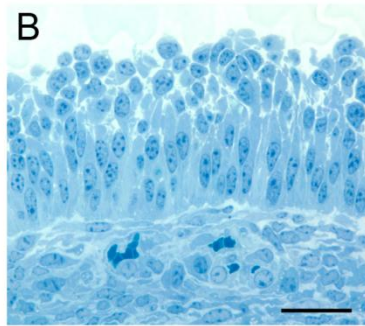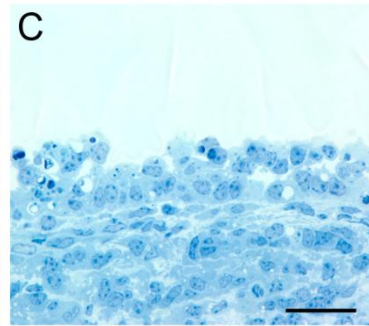

Supplement: Additional file 4: Figure S2 — Histological classification of small antral follicles. Methylene blue stained semi-thin sections of (A) healthy rounded, (B) healthy columnar and (C) atretic small antral follicles. Bar = 50 μm. [file 1471-2164-15-40-S4.pdf]
