# Supplementary material for: Transcriptome profiling of granulosa cells from bovine ovarian follicles during atresia
Source: BMC Genomics. 2014 Jan 18;15:40. doi: 10.1186/1471-2164-15-40 (PMC3898078; doi:10.1186/1471-2164-15-40)
Supplement: Additional file 5: Table S3 — Primary antibodies, secondary antibodies, labelling and fixation conditions used for immunohistochemistry for each antigen. Secondary antibodies used were either biotin-SP-conjugated AffiniPure donkey-anti-mouse IgG (Cat. no. 715-066-151), followed by Cy3-conjugated streptavidin (SA-Cy3, Cat. # 016-160-084) or fluorescein/DTAF-conjugated streptavidin (SA-DTAF, Cat. # 016-010-084), or Cy3-conjugated AffiniPure donkey-anti-rabbit IgG (Cat. # 711-166-152) or anti-mouse (Cat. # 715-166-151) or FITC-conjugated AffiniPure donkey-anti-rat IgG (Cat. # 712-096-153). All secondary antibodies and conjugated streptavidins were purchased from Jackson ImmunoResearch Laboratories Inc. (West Grove, PA, USA) and used at 1:100 dilutions. [file 1471-2164-15-40-S5.pdf]

Antigen (Species)	Primary Antibody				Secondary Antibody	Conjugates*	Fixation
	Host Species	Code or Clone number	Source or Reference	Concentration or Dilution			
Collagen type I (bovine)	Mouse	ab6308, clone COL-1	Abcam[1]	7 µg/ml	Biotin-SP-conjugated AffiniPure donkey anti-mouse	Fluorescein/ DTAF-conjugated streptavidin	100% ethanol
Nidogen 2 (mouse)	Rabbit	ab14513	Abcam[1]	1:200	Cy3-conjugated AffiniPure donkey anti-rabbit	none	100% ethanol
Perlecan (Mouse)	Rat	MAB 1948, clone A7L6	Millipore[2]	10 µg/ml	FITC-conjugated AffiniPure donkey anti-rat	none	10% buffered formaldehyde solution (BFS)
E-cadherin (Human)	Mouse	610181, clone 36	BD Biosciences[3]	2.5 µg/ml	Biotin-SP-conjugated AffiniPure donkey anti-mouse	Cy3-conjugated streptavidin	10% BFS

[1] distributed by Sapphire Bioscience Pty Ltd, Waterloo, NSW, Australia; [2] Millipore Australia Pty Ltd, Kilsyth, VIC, Australia; [3]BD Biosciences Australia, North Ryde, NSW, Australia.
